# Smc5/6-Mms21 Prevents and Eliminates Inappropriate Recombination Intermediates in Meiosis

**DOI:** 10.1371/journal.pgen.1004067

**Published:** 2013-12-26

**Authors:** Martin Xaver, Lingzhi Huang, Doris Chen, Franz Klein

**Affiliations:** Max Perutz Laboratories, Chromosome Biology, University of Vienna, Vienna Biocenter, Vienna, Austria; National Cancer Institute, United States of America

## Abstract

Repairing broken chromosomes via joint molecule (JM) intermediates is hazardous and therefore strictly controlled in most organisms. Also in budding yeast meiosis, where production of enough crossovers via JMs is imperative, only a subset of DNA breaks are repaired via JMs, closely regulated by the ZMM pathway. The other breaks are repaired to non-crossovers, avoiding JM formation, through pathways that require the BLM/Sgs1 helicase. “Rogue” JMs that escape the ZMM pathway and BLM/Sgs1 are eliminated before metaphase by resolvases like Mus81-Mms4 to prevent chromosome nondisjunction. Here, we report the requirement of Smc5/6-Mms21 for antagonizing rogue JMs via two mechanisms; destabilizing early intermediates and resolving JMs. Elimination of the Mms21 SUMO E3-ligase domain leads to transient JM accumulation, depending on Mus81-Mms4 for resolution. Absence of Smc6 leads to persistent rogue JMs accumulation, preventing chromatin separation. We propose that the Smc5/6-Mms21 complex antagonizes toxic JMs by coordinating helicases and resolvases at D-Loops and HJs, respectively.

## Author Summary

Homologous recombination allows repair of DNA breaks from intact templates of identical sequence by a “copy-and-paste” like mechanism. However, the double Holliday Junction (dHJ) is a hazardous intermediate that can form during homologous recombination, if single stranded DNA from both ends of a lesion pair with the template. Once the primary lesion is eliminated, the dHJ connects the chromosomes stably and if unresolved can prevent segregation during cell division. In order to prevent chromosome non-disjunction, resolvases will cut any HJ before division. However, genomes contain many multi-copy DNA sequences as transposons or repetitive elements. If dHJs form between such non-allelic loci, cleavage by resolvases can result in chromosome translocations and deletions. Therefore, stabilization of dHJs is sought to be avoided in the first instance by anti-recombinogenic helicases on early intermediates. Analysis of Smc5/6-Mms21 mutants in meiosis revealed that it antagonizes unregulated dHJs both by prevention and resolution. Elimination of the Mms21 SUMO E3-ligase domain leads to inappropriate dHJ accumulation still resolved by Mus81-Mms4. Disruption of the whole complex results in persistent dHJ accumulation and dysfunction of resolvases, preventing chromatin segregation. These results provide a first unified view on the function of Smc5/6-Mms21 as an antagonist of dangerous dHJs.

## Introduction

Sexual reproduction in eukaryotes relies on the generation of haploid gametes from diploid somatic cells by a process called meiosis. Meiosis achieves the required reduction in ploidy by executing a single round of DNA replication followed by two consecutive rounds of chromosome segregation. The correct segregation of homologous chromosomes depends on the formation of chiasmata, which are crossovers (COs) held in place by distal sister chromatid cohesion [1], [2]. Crossovers, and in many organisms also the identification and pairing of homologous chromosomes, require the repair of programmed meiotic DNA double-strand breaks (DSBs) in prophase I at sites that have completed pre-meiotic DNA replication [3]. As a consequence of the repair of DSBs, stable recombination intermediates called double Holliday Junctions (dHJs) can arise, and can be resolved to generate crossovers (COs) or non-crossovers (NCOs).

Sophisticated mechanisms controlling CO numbers and distribution ensure that each bivalent (two paired homologous chromosomes) receives at least its obligate CO. In budding yeast, the ZMM pathway (an acronym for the involved proteins Zip1-4, Mer3, Msh4/5 [4]) is a key part of this control. It guides a subset of DSBs to become allelic COs between homologs by allowing these breaks to form dHJs specifically resolved to COs depending on Exo1-Mlh1/3 [5]. A sophisticated machinery, including the Synaptonemal Complex (SC), regulates the progression of recombination intermediates in the ZMM pathway. The SC is a tripartite proteinaceous structure connecting bivalents along their whole length at a distance of 100 nm in the pachytene stage of meiosis. After completion of synapsis, Polo-like kinase Cdc5 activation triggers the resolution of dHJs shortly before cells become committed to enter the first meiotic division [6], [7].

Non-ZMM DSBs are not destined to become COs and follow another main route, involving fast and minimal-risk repair by Synthesis Dependent Strand Annealing (SDSA). In SDSA the invasion of one broken DNA terminus into an intact template allows DNA repair synthesis beyond the break. Importantly, interaction remains limited by the rapid displacement of the invading strand. This pathway does not produce COs but may nevertheless facilitate homolog recognition. Recently, the RecQ helicase BLM/Sgs1 [8] has been shown to be a central player in both primary pathways of meiotic recombination [5], [9]–[12]. In the SDSA pathway, Sgs1 promotes strand displacement thereby preventing stabilization of the invasion. In the ZMM pathway, Sgs1 delays repair until the formation of stable Single End Invasion (SEI) and dHJ intermediates is appropriate, presumably after the cell has accumulated some information about the correctness of the invaded target. In budding yeast, the ZMM and SDSA pathways together accomplish the large majority of meiotic recombination events [5].

If recombination intermediates escape the two pathways described above, unregulated, mitotic like Joint Molecules (JMs) can arise, such as dHJs not associated with the proper ZMM machinery. We will refer to these intermediates as “rogue”, in the sense that they are “unprincipled, unreliable and with potentially destructive properties” which could result in non-allelic COs after escaping the two canonical, safe pathways. Although these rogue intermediates constitute only a minor fraction of recombination events in wild type meiosis, they can block chromosome segregation if unresolved. Resolution of such JMs is mediated by the overlapping activity of the three HJ resolvases Mus81-Mms4, Slx1-Slx4, and Yen1 [5], [7], [9]. Cdc5/Plk1 also controls the activation of Mus81-Mms4 and Slx1-Slx4.

dHJs represent potential danger for their inability to elicit a DNA damage checkpoint response [10]. Due to their stability, they may cause chromosome nondisjunction or even block segregation if not resolved. Conversely, resolution of HJ intermediates between non-allelic positions can result in deletions or translocations, even in dicentric chromosomes. This explains attempts of the cell to avoid the formation of such stable recombination intermediates right away, mediated by the action of helicases such as Srs2 and BLM/Sgs1 that can destabilize Rad51 filaments and nascent invasions [13]–[17]. Even if dHJs formed, the cell may be able to unwind them conservatively through the action of Sgs1-Rmi1-Top3 [18], [19], a process termed dHJ dissolution. Ultimately, once the cell has passed the DNA damage checkpoint and is committed for division, resolvases will be given priority for timely removal of linking Holliday Junctions (HJs) to avoid chromosome nondisjunction events. While a key set of DNA metabolizing activities have recently been described, important questions concerning the chromosomal context remain unanswered. Coordination of local recruitment, regulation and orientation of anti-JM helicases and resolvases in the *in vivo* context remain completely obscure to date.

In this study, we provide evidence that the Smc5/6-Mms21 complex mediates such functions. SMC complexes are evolutionarily conserved from gram-negative bacteria to mammals, serving critical functions in chromosome metabolism, thereby helping to preserve chromosomal and genomic integrity. Eukaryotes have three distinct, essential ring- shaped SMC complexes at their disposal; the cohesin complex (Smc1-Smc3), linking sister chromatids until the metaphase/anaphase transition, condensin (Smc2-Smc4), thought to regulate higher order chromosome structure and finally the Smc5/6-Mms21 complex, involved in recombinational DNA repair.

All SMC ring complexes exhibit the ability to associate with DNA and the hinge region of several SMC complexes, including condensin, has been shown to bind to DNA [20]. At least for cohesin and condensin there is direct evidence that DNA is topologically entrapped inside the ring [21]–[24].

While this has not yet been tested for the Smc5-Smc6 complex, it was shown that both Smc5 and Smc6 individually bind stably to DNA with a strong preference for single stranded DNA [25], [26]. Taking into consideration the high similarity in structure and size to the other SMCs, it is not unlikely that Smc5/6-Mms21 also encloses DNA strands topologically.

The SMC proteins consist of two extended domains that fold back on themselves at their central hinge region into an anti-parallel coiled-coil structure. This brings the two terminal Walker A/B motifs in close proximity to form ABC-like ATPases. Two SMC proteins connect via their hinge regions, while the kleisin subunit closes the ring by linking the terminal SMC ATPase heads. Different SMC complexes contain characteristic non-SMC subunits that form integral components of the functional complex.

**Figure 1:**
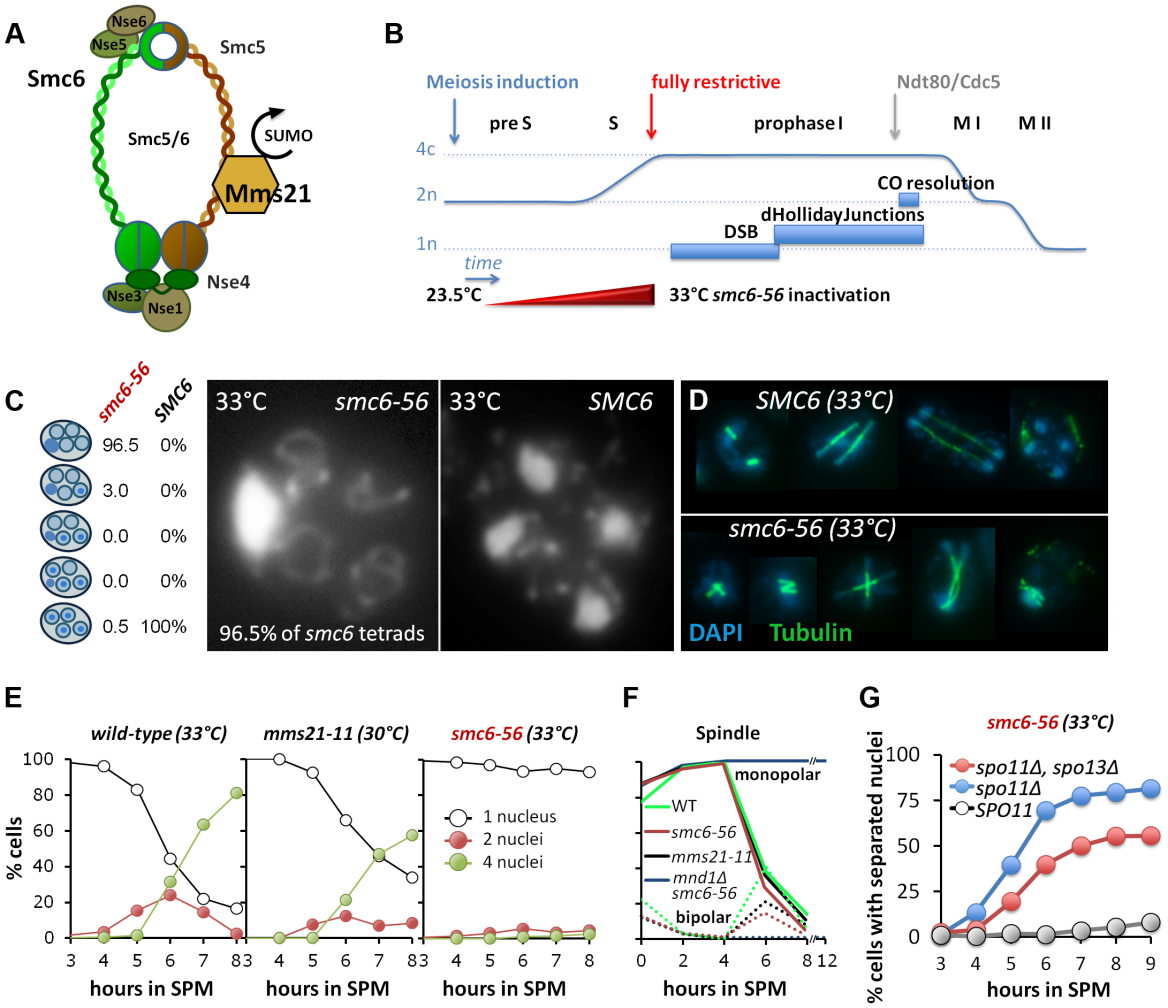
Smc6, but not Mms21 SUMO ligase activity is required for chromosome segregation after meiotic recombination. (A) Schematic representation of the Smc5/6-Mms21 subunits. (B) Schematic of the temperature shift experiments. To avoid mitosis and premeiotic S-phase a temperature shift from 23.5 to 33°C was carried out in increments upon induction of meiosis (see experimental procedures) reaching 33°C by 2.5 hours in SPM when cells are past bulk DNA replication. This scheme eliminates Smc6 at the point when the earliest DSBs become detectable and interference with mitosis and pre-meiotic S-phase is little. (C) DNA staining with DAPI after spore formation. The right micrograph shows the presence of 4 nuclei and mitochondrial DNA in a wild type tetrad. The left micrograph shows the nuclear DNA outside the four spores, pressed against the ascus wall, while only the mitochondrial DNA had segregated into the spores. Left table: Percent of asci with 0,1,2,3,4 spores containing nuclear DNA (n = 200 tetrads). (D) Spindle staining by anti-tubulin. The upper panel shows examples of spindle morphologies post anaphase I in wild type. The lower panel shows aberrant spindles of the *smc6-56* mutant, consistent with physical impediment of DNA separation. (E) Meiotic nuclear divisions are blocked in *smc6-56* mutants at 33°C. Left panel: wild type (33°C), middle: *mms21-11* (30°C), right: *smc6-56* (33°C). Empty black circles: cells containing one DAPI stained nucleus (1n), filled red circles: cells with 2 nuclei (2n), filled green circles: cells with 4 nuclei (4n). (F) Meiotic progression is normal in *smc6-56* (33°C): Meiotic progression was followed by spindle morphology by tubulin labeling in an *in situ* staining procedure. Monopolar and bi-polar spindles were plotted separately against hours in SPM. n = 200 cells per experiment, continuous lines: monopolar spindles, dotted lines: bipolar spindles. Green: Wild type (30°C), black: *mms21-11* (30°C), red: *smc6-56* (33°C), blue: *mnd1Δ smc6-56* (33°C). (G) *spo11Δ* suppresses the chromatin separation defect of *smc6-56*. Cells containing at least 2 separated nuclei plotted against the time in sporulation. Grey filled circles: *smc6-56 SPO11*, blue filled circles: *smc6-56 spo11Δ*, red filled circles: *smc6-56 spo11Δ spo13Δ*.

In budding and fission yeast the Smc5/6-Mms21 complex comprises six Non-SMC Elements (NSEs), Nse1 to Nse6, of which Nse4 is the kleisin (Figure 1A). Beside Smc5 and 6, at least Nse1 to Nse4 are conserved from yeast to man [27]–[29].

A unique feature of the Smc5/6-Mms21 complex, compared to cohesin and condensin, is its SUMO E3 ligase subunit Mms21/Nse2 [27], conferring the ability to post-translationally modify target proteins. The SUMOylation substrates of Mms21 remain poorly defined to date, however, candidates include Smc5, Scc1, the kleisin subunit of cohesin, as well as telomeric proteins [30],[31].

Mms21 is stably bound through an extensive N-terminal coiled-coil interface to the coiled coil domain of Smc5 [32], [33] while the E3 SPL RING structure that recruits the SUMO E2 ligase Ubc9 resides at its C-terminus [32]. Mms21 is an essential subunit of the Smc5/6-Mms21 complex in *Saccharomyces cerevisiae* and the mere disruption of its interaction with Smc5 is lethal [32], [34]. Notably, elimination of Mms21's SUMO E3 ligase activity alone is not lethal, but sensitizes the cell to genotoxic agents [27].

Yeast cells mutated in the Smc5/6-Mms21 complex become hypersensitive to genotoxic agents including hydroxyurea, MMS, ionizing irradiation and UV [27], [35], [36], and DNA regions vulnerable to homologous recombination (HR) like rDNA and telomeres are particularly affected [37]. Accordingly, Smc5/6 was also found to accumulate at such sites prone to recombinogenic damage [38], [39]. Accumulation of X-shaped DNA intermediates upon challenge and aberrant processing of stalled replication forks have been reported in Smc5/6 mutants [40] and roles for the Smc5/6-Mms21 complex in multiple repair pathways, including homologous recombination, have been suggested [41]. Complete elimination of functional Smc5/6-Mms21 complex from vegetative cells leads to heterogeneous defects, including cells arresting in metaphase, chromosome missegregation and eventually lethality [42].

In meiosis, phenotypes of Smc5/6 mutants in *S. pombe* and *S. cerevisiae* represent exacerbated manifestations of those observed during mitosis, including catastrophic failures in meiotic divisions [43], [44]. In a synapsis mutant (*zip1Δ*) of *S. cerevisiae*, the homologous chromosomes tended to become more attached to each other and chromosomal entanglements seemed to increase. These defects were partly caused during premeiotic S-phase, as they were not fully dependent on initiating meiotic recombination [44]. Another study in *S. pombe* found accumulation of X-shaped DNA molecules in *nse6* mutants, originating from meiotic recombination [43].

Here we study the role of Smc6 in meiotic recombination by eliminating Smc6 after premeiotic S-phase is largely complete. This leads to an accumulation of unresolved JMs at meiotic recombination hotspots and a corresponding uniform block in nuclear divisions. We observe strong overlaps of Smc6-chromatin binding with that of Sgs1 at a substantial number of meiotic DSB-hotspots. In chromosome spreads, Smc5/6 foci co-localize with Rad51/Dmc1 foci side by side. We further show that the E3 ligase deficient *mms21-11* allele restores dHJ and CO formation and improves spore viability in the robust ZMM mutant *zip3Δ*, implying an important role of Mms21 in the prevention of dHJ formation in this background. Resolution of the JMs in the *mms21-11* mutant depends on the Mus81-Mms4 resolvase. The Smc5/6-Mms21 complex is also required for HJ resolvase activity responsible for eliminating rogue HJ intermediates. A dramatic accumulation of unresolved JMs and a pronounced reduction in COs and NCOs was observed in the *smc6-56 sgs1* double mutant, in which all meiotic recombination follows an aberrant non-ZMM, non-SDSA pathway, while COs form at near normal levels in the *smc6-56 SGS1*. We conclude that Smc5/6-Mms21 collaborates with helicases and resolvases to both prevent and eliminate JMs that arise outside the ZMM recombination pathway.

## Results

### Smc6, but not Mms21 SUMO ligase activity is required for chromosome segregation after meiotic recombination

In order to characterize the role of the Smc5/6-Mms21 complex in the context of meiotic recombination, we utilized two previously described mutants with distinctive phenotypes. *smc6-56* is a temperature sensitive allele, carrying three missense mutations in the N-terminus proximal coiled-coil region (A287V, H379R, I421T) conferring lethality at restrictive temperature [45]. The *mms21-11* allele terminates after Thr183 and lacks the C-terminal SPL-RING domain, thus depriving Mms21 of its SUMO E3 ligase activity by abolishing its interaction with the SUMO E2 enzyme Ubc9 [27], [46].

To distinguish the role of the Smc5/6-Mms21 complex in meiotic DSB repair from that of mitosis and premeiotic S-phase we inactivated the *smc6-56* allele by shifting the temperature of the synchronized cultures gradually to restrictive conditions at 33°C (Figure 1B). Cells are allowed to exit mitosis and undergo most of meiotic DNA replication under permissive or semi-permissive conditions (Figure S1A, B). Fully restrictive conditions were applied from 2.5 hrs post induction of meiosis, when most cells are in late S-phase and the earliest meiotic DSBs become detectable [47]. The constitutive *mms21-11* mutant was analyzed at 30°C.

Under these conditions, *smc6-56* causes neither a delay in meiotic progression and spore formation, nor does it produce evident defects in meiotic DSB formation, repair, or chromosome axis architecture. Chromosome synapsis is flawless with normal timing (Figure S1C), meiosis I and meiosis II spindles form with normal kinetics, and a *mnd1Δ smc6-56* double mutant arrests indistinguishably from *mnd1Δ* which prevents DSB repair at the strand invasion step [48]–[50] in prophase1 indicating a functional DNA damage checkpoint (Figure 1F), suggesting efficient DSB-turnover in *smc6-56* comparable to wild type.

However, 92–99% of cells with inactivated Smc6 failed to segregate the chromatin during both meiotic divisions and thus produced a single nucleus outside of four empty spores (Figure 1C, E). Unable to separate the chromatin, anaphase I and II spindles ultimately collapse, resulting in aberrant spindle morphologies from Meiosis II onwards (Figure 1D). The timing of spore formation and the number of cells forming spores was as in wild-type, however, the number of aberrant asci containing one, two or three spores was increased at the expense of complete tetrads (21–27%, less than half of wild type). In 88–95% of the tetrads, all four spores were empty and only 0.5–4.5% of tetrads had DNA in all four spores. Most empty spores ultimately collapse and partially lyse by 24 hours. Notably, those few tetrads that managed to receive DNA in all 4 spores and survive zymolyase digestion show a fairly high spore viability (53.75%, n = 80 spores/20 tetrads).

To test whether the observed meiotic catastrophe depended on meiotic recombination, DSB formation was abolished by introducing the *spo11Δ* mutation in the *smc6-56* background. Indeed, meiotic divisions were largely restored with 80% of the cells completing at least one division and 60% of the cells finishing both meiotic divisions (Figure 1G). To demonstrate that also the sister chromatids can separate, the triple mutant with *spo13Δ* was analyzed. We found that almost 60% of the cells underwent the single division with normal kinetics (Figure 1G). Thus, the applied regime of conditional inactivation of Smc6 separates most of the defects related to mitosis and meiotic S-phase from those in meiotic recombination. We conclude that the Smc5/6-Mms21 complex is essential for allowing chromosome disjunction upon initiation of meiotic recombination.

In contrast to the *smc6-56* mutant, *mms21-11* showed almost normal kinetics of meiotic divisions, forming tetra-nucleated cells with only a slight delay (Figure 1E). Synapsis and sporulation are efficient (Figure S1C) and the resulting tetrads exhibited high spore viability (88.75%; n = 400 spores of 100 tetrads). Only a small fraction of tetrads (3–3.25%) were missing DNA in one or more spores. Consequently, the SUMO E3 ligase activity of the Smc5/6-Mms21 complex is not essential to prevent massive chromosome non-disjunction.

### The Smc5/6-Mms21 complex binds early to meiotic chromatin and associates with sites of DSB repair

To characterize the binding of Smc5/6-Mms21 to meiotic chromatin in the context of meiotic recombination we used the epitope tagged *SMC6-myc13* construct and prepared meiotic nuclear spreads from synchronized cultures at various time points for cytology. Smc6-myc13 does not show any obvious defects and co-localizes with Smc5-HA3 on chromosome spreads, suggesting it is a valid representation of the complex (Figure S2A).

**Figure 2:**
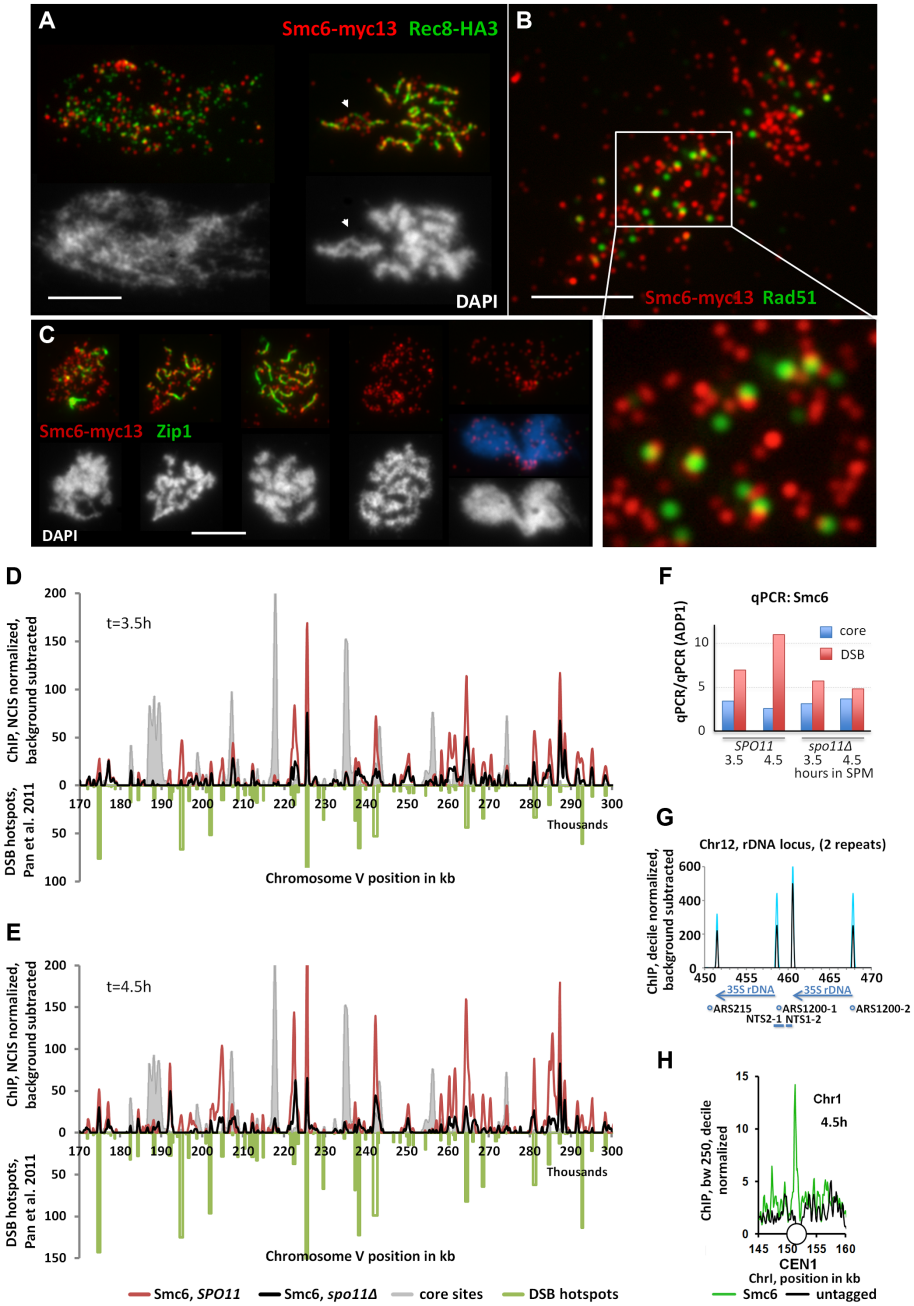
The Smc5/6-Mms21 complex binds early to meiotic chromatin and associates with sites of DSB repair. (A) Co-immuno labeling for Smc6-myc13 and cohesin subunit Rec8-HA3 of a spread, leptotene nucleus and pachytene nucleus. Red: Smc6-myc13, Green: Rec8-HA, white bar: 5 µm. (B) Co-immuno labeling for Smc6-myc13 and recombinase Rad51 of a spread meiotic prophase I nucleus. Red: Smc6-myc13, Green: Rad51, white bar: 5 µm. White rectangle indicates position of magnified sub region. (C) Co-immuno labeling for Smc6-myc13 and synapsis specific Zip1 protein of several meiotic stages. (Figure S3 displays a complete series of double stained nuclei, staged according to Zip1 and DNA morphology): Red: Smc6-myc13, Green: Zip1, White (and blue) figures below the immunostained nuclei represent the corresponding nuclear DNA stained with DAPI. White bars: 5 µm. Stages correspond to (from left to right): Early zygotene, late zygotene, pachytene, diplotene, late anaphase I. (D) Smc6-myc13 (t = 3.5 hours in SPM) localizes at DSB sites. ChIP-seq signals on a 130 kb region of chromosome V are shown after smoothing (bandwidth: 500 bp), NCIS normalization and background subtraction as described in Materials and Methods. Red: Smc6-myc13, black: Smc6-myc13, *spo11Δ*, filled grey profile: Mer2-HAint. (t = 4h) to illustrate core site signals. DSB hotspots defined in [53] were plotted in green on the negative scale to indicate their positions and relative intensities. The diagram on the right shows the results of qPCR at three positions on chromosome III from the same experiment: a DSB site (ca. at 211k, YCR047C), a core site (ca. at 219k) and a cold spot (ca. at 136, ADP1). To correct for possible differences in the efficiencies of the IPs across the different strains, the enrichment of core and DSB qPCR signals relative to the ADP1 signal per ChIP is plotted for the indicated genotypes and time points. Core/ADP1 shown in blue, DSB/ADP1 in red. (E) Same as (D) but Smc6-myc13 analyzed at t = 4.5 hours in SPM. (F) qPCR on chromosome III from the same experiment as in (D,E), a DSB site (ca. at 211k, YCR047C), a core site (ca. at 219k) and a cold spot (ca. at 136, ADP1). To correct for possible differences in the efficiencies of the IPs across the different strains, the enrichment of core and DSB qPCR signals relative to the ADP1 signal is plotted for the indicated genotypes and time points, Core/ADP1 shown in blue, DSB/ADP1 in red. (G) Representation of the rDNA locus on chromosome XII as provided by the Saccharomyces Genome Database (SGD) showing two (of approximately 200) rDNA repeats. Profiles for Smc6-myc13 at 4.5 hours in SPM are shown (blue: WT, black: spo11Δ; smoothed at bandwidth 250bp, decile normalized and background subtracted (minus untagged)). Sharp Smc6 signals flank the 35S rDNA transcriptional unit independent of Spo11. Positions of replication origins (ARS), nontranscribed spacers (NTS) and RDN37 repeats (blue arrows) are indicated below. (H) Centromeres display a Smc6 signal. As in (G) but without background subtraction for a small region around CEN I (white circle). Green: Smc6-myc13, t = 4.5, black: untagged.

Smc6-myc13 localizes in individual foci which appear early in meiosis at approximately the same time as Rec8 (Figure S2B) and soon accumulate to considerable numbers until earliest prophase 1 (Figure S3). We used antibody-staining against the synapsis specific Zip1 protein for staging (from isolated Zip1 foci up to full SCs). In nuclei engaged in meiotic recombination (Zip1 positive) an average of 118±18 (n = 21 nuclei scored) Smc6 foci were scored and this number did not change significantly in different stages of prophase 1. After prophase 1, the intensity of foci decreased slightly, but numbers remained high until Metaphase II/Anaphase II transition (Figure 2C, S3). A particular enrichment was observed for the rDNA region on chromosome 12, which remains unsynapsed in the course of meiotic recombination (Figure 2A white arrows, S3).

The preferred binding sites of Smc6 in wild-type meiosis differ markedly from those of the related cohesin complex, which binds specifically to chromosome regions that constitute the chromosome axis upon condensation [1], [51], [52]. On meiotic chromatin, foci of Smc6-myc13 and Rec8-HA3 (a tagged version of the meiotic kleisin-subunit of cohesin) generally exclude each other (Figure 2A, S2B). When chromosomes condense, Smc6 signal often protrudes from the Rec8-axes, suggesting preferential binding of the Smc5/6-Mms21 complex to DNA regions not associated with the axis (Figure 2A).

In order to determine the chromosomal binding sites of Smc5/6-Mms21 complex, ChIP-Seq (Chromatin-Immuno Precipitation followed by next generation sequencing) of Smc6-myc13 was performed. Synchronous meiotic cultures were crosslinked with formaldehyde, subjected to ChIP and the precipitated DNA deep sequenced on an Illumina platform.

Figure 2D, E show a 130 kb representative region of Chromosome V with about 55 medium to weak DSB hotspots mapped previously [53]. Figure 2D, E demonstrate that the majority of Smc6 peaks localize precisely to these hotspots. In the example shown, the 3.5 hour Smc6 peaks fall into 45 (82%) hotspots, while 49 (89%) hotspots coincide with a 4.5 hour Smc6 peak. The vast majority of peaks are precisely on top of the hotspots (up to 80% genome wide), however, we note that the intensities of the co-localizing Smc6 peaks are often not proportional to the intensity of the break site, resulting in the relatively low genome wide Pearson correlations despite precise co-localization.

To address whether Smc6 localization depended on the formation of DSBs by Spo11, the experiment was repeated in a *spo11Δ* mutant. A genome wide reduction of Smc6 at DSB sites was observed, however, surprisingly, Smc6 still accumulated at the majority of hotspots (Figure 2D, E, S4). The genome wide ChIP experiments were accompanied by qChip at a DSB hotspot (YCR047, 211k), a core site (219k) and a cold region (ADP1, 136k) all on chromosome III (see Figure S4). qChIP confirmed the results on the corresponding positions of the ChIP-Seq profiles (Figure 2F). The ChIP-Seq experiments were repeated confirming the reported results (not shown). The qPCR of the biological repeat confirmed the Spo11 improved enrichment of Smc6 at the hotspot precisely (Figure S4C). Not all binding sites of Smc6 are DSB-sites. For instance, sharp Smc6-peaks mark all the centromeres (Figure 2H, S4A,B). Furthermore, confirming our cytological observation of abundant rDNA localization of Smc6 foci, Spo11 independent Smc6 signals flank the 35S rDNA transcriptional units (Figure 2G). Binding of Smc6 to the rDNA region is in agreement with the crucial anti-recombination role that Smc5/6-Mms21 plays at this repetitive DNA locus [37]. For the remaining Smc6 signal we often observe overlaps with meiotic chromosome axis sites as defined by Mer2 and Hop1 [51]. Such axis specific enrichment for Smc6-myc13 is, however, rather low (Figure 2, S4). Similar conclusions have been drawn in the study of Copsey and coworkers (accompanying manuscript) [54], although more prominent binding of Smc5 at core sites is found than in our study. The quantitative discrepancies in the recovery for Smc5 and Smc6 may reflect different biological properties of the complex. For instance, it was reported for the related cohesin complex, that different cohesin populations exist regarding stability of chromatin binding [55]. Further, we observed previously that the choice of a tag can influence the balance between core and DSB site residency [51], [56]. Differences can also arise due to the different subunits analyzed, the different platforms and resolutions used and the highly stringent background subtraction that we use. Importantly, both studies observe signals consistent with a role of Smc5/6 at sites of meiotic recombination.

Due to observed recruitment of Smc6 to DSB hotspots, we asked whether Rad51, the eukaryotic strand exchange protein which assembles along the resected DSB-ends and facilitates the strand invasion step in HR repair, co-localizes with Smc6-myc13. The number of Smc6-myc13 foci far exceeded (about 4-fold) the Rad51 foci, consistent with DSB independent loading of Smc6 to chromatin. Strikingly, we found almost no on-top co-localization of Smc6 with Rad51 (Figure 2B). Counting foci on 6 meiotic nuclei confirmed this impression. Only 0–10% of the Rad51 foci directly co-localized with Smc6 (on average 6.5±4%). However, a total of 85±4% (n = 6 nuclei scored) Rad51 foci were in a side-by-side configuration with one or two Smc6 foci, even on nuclei with strongly spread chromatin (Figure 2B). A similar relation of Smc6-myc13 with the ZMM-DSB marker protein Zip4-myc9 (Figure S2C) was observed.

In summary, we identify a precise and sensitive association of Smc6 with meiotic recombination hotspots and enrichment of Smc6 to DSB hotspots upon actual DSB formation. The frequent side-by-side co-localization of Smc6 and Rad51 recombinase foci is indicative of a primarily spatial separation with juxtaposed positioning of the two complexes.

### The Smc5/6-Mms21 complex is required to prevent the accumulation of toxic Joint Molecules

Unresolved Holliday Junctions (HJs) stably connect chromosomes that had engaged in homologous recombination and thus may result in chromosome nondisjunction. A sufficiently large number of such unresolved recombination intermediates prevent nuclear divisions resulting in mitotic or meiotic catastrophe [7], [10], [12]. Since previous studies reported an accumulation of repair intermediates in mutants of the Smc5/6-Mms21 complex [37], [57], [58], we asked whether an accumulation of unresolved recombination intermediates in the *smc6-56* mutant might explain the Spo11 dependent failure in nuclear divisions.

**Figure 3:**
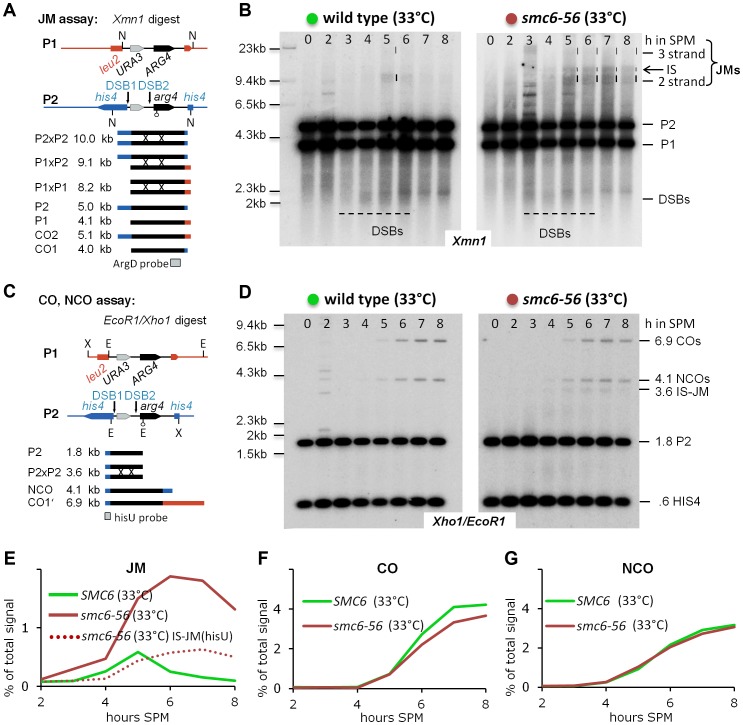
The Smc5/6-Mms21 complex is required to prevent the accumulation of toxic Joint Molecules. (A) Schematic representation of the genetic loci, restriction sites and the probe used to demonstrate joint molecule formation (modified from [6]). (B) Joint molecules accumulate and persist in *smc6-56*. Southern blotting of samples taken from synchronous meiotic time courses at restrictive temperature with DNA extracted and digested with Xmn1 under conditions preserving JMs and using the probe indicated in (A). Lane number indicates hours in SPM. Size markers are provided to the left of the blots while the identity of the labeled species is indicated to the right. The bracket indicates the region where various JMs (2 strand (IH and IS), 3 strand or 4 strand JMs) migrate to. P1, P2: parental fragments. Dashed lines highlight the regions on the blot where DSB signals or JM signals appear. Left Panel: Wild type, right panel: *smc6-56*. (C) Schematic representation of the genetic loci, restriction sites and the probe used to detect and quantify IS-JMs, COs and NCOs (modified from [6]). (D) As in (B) from the same time course experiment but genomic DNA digested with Xho1 and EcoR1 and probe corresponding to (C). Note: hisU probe detects only one parental fragment (P2). (E) JMs accumulate and persist in *smc6-56* at restrictive temperature. Total JM signals were quantified, subtracted from background and plotted as % of total signal as a function of time in SPM. Green: Wild type (33°C), red: *smc6-56* (33°C), red dotted line: Inter sister JM from the blot shown in (D). (F) Near normal levels of CO in *smc6-56*: Representation as in (E) but quantification of CO product. (G) Normal levels of NCO in *smc6-56*: Representation as in (E) but quantification of NCO product.

Meiotic recombination was followed in a physical assay at the *URA3-arg4* locus under conditions that preserve HJ intermediates [59]. This Southern based assay allows quantification of the key intermediates of meiotic recombination: DSBs, dHJ intermediates, as well as crossover and non-crossover recombination products due to restriction polymorphisms between the parental chromosomes (Figure 3A). Using this recombination reporter system, wild type and mutant strains were analyzed under restrictive conditions for *smc6-56* in a time course experiment. Joint molecule levels were measured by probing against *ARG4* DNA on genomic XmnI restriction-fragments. No differences were noted in the transient appearance of DSBs.

However, while joint molecules appear transiently in wild type with low steady state levels and a clear peak at 5 hours when cells are in the pachytene stage of meiosis, the *smc6-56* mutant accumulated high molecular weight recombination intermediates that failed to be resolved (Figure 3B). The observed persistent joint molecules in *smc6-56* amount to more than twice that of the JM peak in wild type, exceeding the amount of stable joint molecules reported to block chromosome segregation (for example in the *mms4-mn yen1Δ* double mutant [9]). In addition, while joint molecules rarely form between sister chromatids in wild type (IS:IH≤0.2), in the *smc6-56* mutant a substantial amount of inter sister JMs (IS-mom:IH 0.32–0.38, by two different assays; XmnI and XhoI/EcoRI) contribute to the overall persistent JMs (Figure 3B, D). Using a *HIS4* fragment to probe for COs and NCOs after a different digest (XhoI/EcoRI) revealed that the levels of both these recombination products were not significantly altered in *smc6-56* (Figure 3C, D, F, G). Consequently, the sum of COs, NCOs and JM-intermediates in the mutant exceeds corresponding numbers in the wild type, uncovering a role of Smc5/6-Mms21 in early destabilization of intermediates to prevent stable JM formation.

In summary, the Smc5/6-Mms21 complex is required to prevent the accumulation of aberrant, unresolved recombination intermediates. The results further suggest that most of the stabilized JMs arise from recombination events not resulting in CO or NCO products in wild type, such as inter sister SDSA or dissolution of rogue dHJs. In particular, this suggests that the CO specific ZMM-pathway is not affected by the defect in *smc6-56*.

### Mms21 SUMO E3 ligase activity prevents tight homolog associations in *zip3Δ* mutants

If the *smc6-56* mutant confers persistent joint molecules to the cell that impede nuclear divisions, then there are two feasible explanations as to why the *mms21-11* mutant does not. Either *mms21-11* represents a plain Smc5/6-Mms21 hypomorph in meiosis with remaining activity at a level such that formation of aberrant joint molecules is negligible. Alternatively, *mms21-11* may represent a separation of function mutant of the complex in which prevention of aberrant JM formation and the resolution of such by the Smc5/6-Mms21 complex had become separated. If the latter is true, it should be possible to detect inappropriate joint molecule formation as well as their removal in *mms21-11*.

To test if the *mms21-11* mutant indeed fails to prevent the formation of additional and atypical joint molecules in considerable amounts, we assayed on surface spread meiotic nuclei for the presence of excess axial associations in the background of the ZMM mutant *zip3Δ*. In mutants of the ZMM pathway chromosome synapsis is defective, the repair of ZMM-breaks is inhibited through activity of the Sgs1 helicase [11], and dHJ and CO formation are impaired while Non-ZMM breaks are repaired by SDSA [4]. Axial associations as first described in the *zip1Δ* mutant are the cytological manifestation of stable recombination intermediates between the homologs, approximating dHJs and COs numbers in wild type [60]. In the SK1 strain background, *zip3Δ* confers one of the most severe ZMM phenotypes, exhibiting a strong reduction in JMs and a robust prophase I arrest [4].

**Figure 4:**
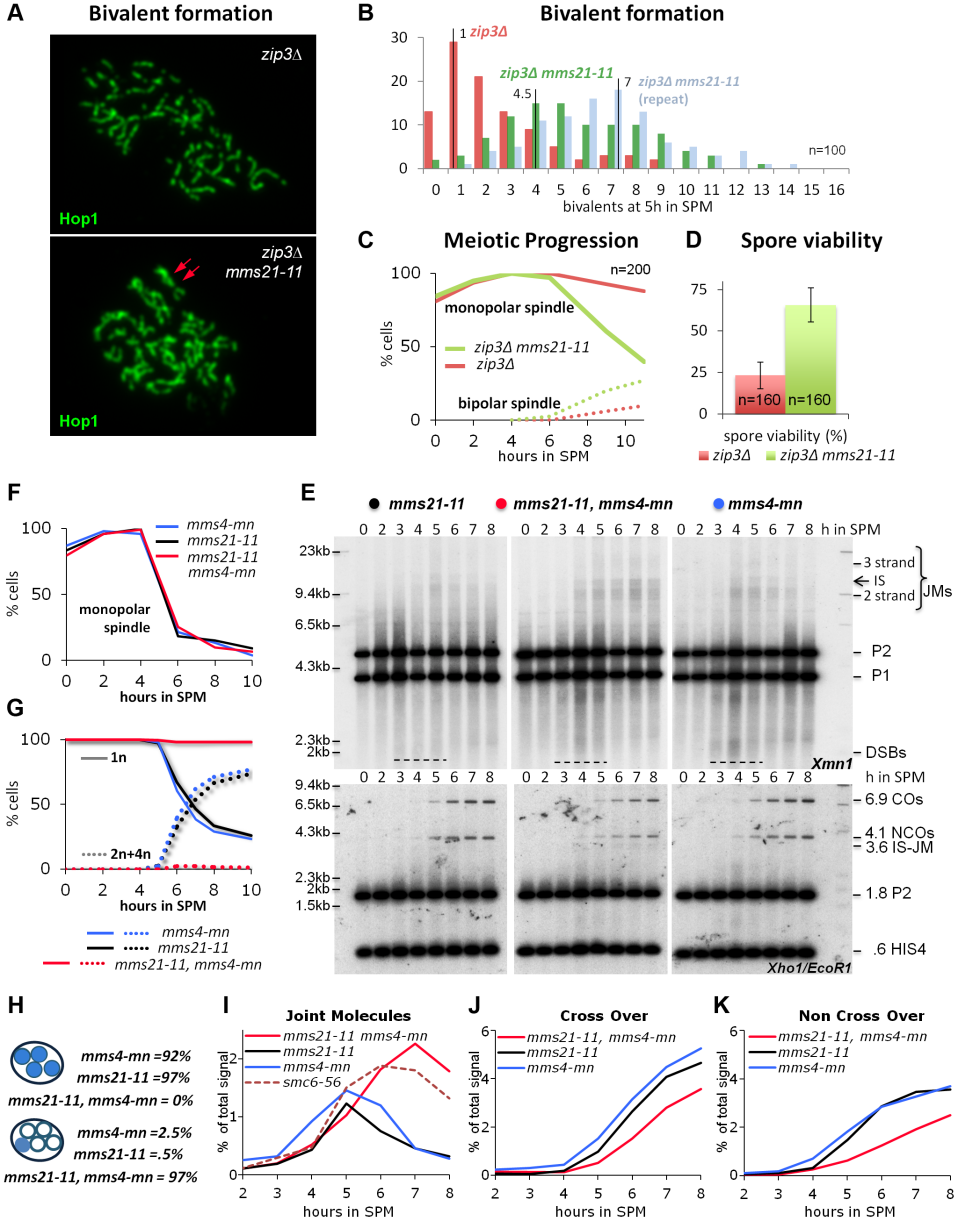
Mms21 SUMO E3 ligase activity antagonizes inappropriate Joint Molecule formation in *zip3Δ* and in *mms4-mn* mutants. (A) *mms21-11* restores bivalent formation and axial associations in *zip3Δ mutants*. Shown are representative nuclei after chromosome spreading and immunolabeling with Hop1 to visualize chromosome axes. Top panel: *zip3Δ*. Bottom panel: *zip3Δ mms21-11* double mutant. Red arrows point at a *mms21-11* dependent bivalent with apparently two axial associations. Several such bivalents emerge in this nucleus. (B) Quantification of bivalent formation: Spread nuclei were classified according to the number of bivalents present. The discrete density of the distribution is plotted (incidences against class). n = 100 nuclei were classified per experiment. Red: *zip3Δ*, green and blue: two biological repeats of *zip3Δ mms21-11*. (C) *mms21-11* improves meiotic progression in *zip3Δ*: Meiotic progression was followed via spindle morphology. Monopolar and bi-polar spindles were plotted against hours in SPM. n = 200 cells per experiment, continuous lines: monopolar spindles, dotted lines: bipolar spindles, red: *zip3Δ*, green: *zip3Δ mms21-11*. (D) *mms21-11* improves spore viability in *zip3Δ*: Spore viability was assayed by tetrad dissection in n = 40 tetrads per mutant, red: *zip3Δ*, green: *zip3Δ mms21-11*. (E) JMs accumulate in *mms21-11* that require the Mus81-Mms4 resolvase for resolution: Same JM, CO and NCO assay as described in Figure 3 (A through D). Southern blotting of samples from synchronous meiotic time courses at 30°C. Upper panels: Xmn1 digest, JM detection. Left: *mms21-11*, Middle: *mms21-11 mms4-mn*, Right: *mms4-mn*. Lower panels: Xho1/EcoR1 digest, CO/NCO detection. Left: *mms21-11*, Middle: *mms21-11 mms4-mn*, Right: *mms4-mn*. (F) Meiotic progression is normal in all *mms21-11 and mms4-mn* mutants: Meiotic spindles were labeled. Monopolar spindles were plotted against hours in SPM. n = 200 cells per experiment, continuous lines: monopolar spindles. Red: *mms4-mn mms21-11*, black: *mms21-11*, blue: *mms4-mn*. (G) Meiotic nuclear divisions are blocked in *mms4-mn mms21-11* double mutants. Continuous lines: cells containing one DAPI stained nucleus (1n), dotted lines: cells with 2 or 4 nuclei/DAPI-bodies (2n+4n). Red: *mms4-mn mms21-11*, black: *mms21-11*, blue: *mms4-mn*. (H) Segregation of DNA into spores: Numbers represent the percentage of tetrads with either DNA in all 4 spores (upper 3 lines), or all chromosomes outside the spores (lower 3 lines). (I) JMs accumulate and persist in *mms4-mn mms21-11* double mutants. Total JM signals were quantified, subtracted from background and plotted as % of total signal as a function of time in SPM. Red: *mms4-mn mms21-11*, black: *mms21-11*, blue: *mms4-mn*, red dashed line: *smc6-56* (33°C). (J) Reduced levels of CO in *mms4-mn mms21-11* double mutants: Representation as in (I) but quantification of CO product. (K) Reduced levels of NCO in *mms4-mn mms21-11* double mutants: Representation as in (I) but quantification of NCO product.

Accordingly, at 5 hrs in SPM, when in wild type cells most homologs are synapsed, univalents almost bare of axial associations and recognizable pairing dominate the *zip3Δ* mutant phenotype (Figure 4A). 63% of nuclei have no more than 2 chromosomes per nucleus paired or connected by axial associations (n = 100; Figure 4B). In contrast, pairing and axial associations are frequent in the *zip3Δ mms21-11* double mutant, with 5–6 chromosome pairs on average in one experiment and as many as 7 in a biological repeat (n = 100; Figure 4A, B). A similar improvement in pairing can be seen for *zip3Δ smc6-56*, but not for *mnd1Δ* mutants, which are defective in the strand invasion step (Figure S5A) [48], [49].

In addition to the increased number of axial associations, *mms21-11* ameliorated the pachytene arrest of *zip3Δ*. Following the spindle morphology as a marker of meiotic progression, we find 88% of *zip3Δ* cells at 11 hrs still in prophase I, whereas 60% of the *zip3Δ mms21-11* cells had at the same time already progressed beyond prophase I (n = 200; Figure 4C). Additionally, sporulation of *zip3Δ* was improved from 12.75% to 22.5% in the double mutant at 24 hours (n = 400). Thus, ZMM DSBs of *zip3Δ* are turned over in the *mms21-11* background into intermediates that do not elicit a DNA damage checkpoint response. The suppression of the prophase I arrest is not due to a checkpoint defect because the resulting tetrads of the double mutant exhibit a greatly improved spore viability of 65.63%, from 23.13% in *zip3Δ* (n = 160 spores; Figure 4D). The opposite would be expected for a checkpoint failure. Impairment of an early anti-recombinogenic function and inappropriate transformation of DSBs to JMs in mutants of the Smc5/6-Mms21 complex is further supported by the observation that neither absence of the three resolvases Mus81-Mms4, Slx1-Slx4 and Yen1, responsible for the removal of unregulated JMs [5], [9], nor the absence of all four meiosis relevant resolvases, Mlh1/3-Exo1, Mus81-Mms4, Slx1-Slx4 and Yen1 which account for ≥90% of the meiotic resolution activity [5] can improve chromosome pairing in *zip3Δ* (Figure S5B,C in comparison to *nse4-mn* and *sgs1-mn*, meiosis specific null alleles of the Smc5/6 kleisin subunit and the Sgs1/BLM helicase, respectively).

These results indicate that the SUMO E3 ligase deficient *mms21-11* allele fails to antagonize dHJ-formation during DSB repair in the *zip3Δ* mutant, thereby improving bivalent formation, facilitating meiotic progression and ultimately greatly improving spore viability. This is similar to the effect of *sgs1-mn* (*zip3Δ sgs1-mn*: 71.25%, n = 160 spores). While restoration of COs in *zip3Δ mms21-11* remains to be confirmed by physical analysis, we conclude that Mms21 as part of the Smc5/6 complex is specifically required for an early, antagonistic function of the complex in meiotic recombination, most likely by preventing the formation of illegitimate joint molecules, a role previously reported for the helicase BLM/Sgs1 [10]–[12], [61].

### Lack of the Mms21 SUMO E3 ligase leads to rogue HJ intermediates, depending on Mus81-Mms4 for resolution

Three resolvases with overlapping function are responsible for eliminating rogue HJ intermediates in budding yeast: Mus81-Mms4, Slx1-Slx4, and Yen1. JMs arising outside the ZMM pathway depend on these three partially redundant resolvases for resolution, with the XPF family nuclease Mus81-Mms4 showing the biggest contribution [5], [9]. If, as the Zip3 results suggest, considerable amounts of rogue joint molecules form also in the *mms21-11* single mutant, these joint molecules must apparently be efficiently removed prior to nuclear divisions as implied by functional chromatin segregation and high spore viability in *mms21-11*. The most important “rogue JM resolvase” Mus81-Mms4 is the most likely candidate to carry out this vital role. If *mms21-11* represented a separation of function mutant of the Smc5/6-Mms21 complex, unable to prevent aberrant JMs, additional inactivation of Mus81-Mms4 should block nuclear divisions, thereby recreating the full *smc6-56* mutant phenotype.

To test this hypothesis Mus81-Mms4 function was eliminated in the *mms21-11* background using a characterized meiotic null allele of *MMS4* (*mms4-mn*) [10]. Indeed, in the *mms21-11 mms4-mn* double mutant meiotic segregation of chromosomes is blocked, resulting in meiotic catastrophe, while only rarely a cell of the single mutants shows this defect (Figure 4G,H). Chromosome synapsis and meiotic progression were not different from wild type for the single mutants or the double mutant (Figure 4F). As predicted by these results, persistent JMs accumulated in the *mms21-11 mms4-mn* double mutant at levels comparable to those found in the *smc6-56* mutant while the JMs of both single mutants were successfully resolved (Figure 4E,I).

In wild type meiosis, most COs are formed in the ZMM pathway from dHJs resolved via Exo1-Mlh1/3, while the NCOs arise from non-ZMM DSBs repaired through SDSA [5], [9]. In the *mms21-11* mutant, a fraction of both COs and NCOs become dependent on MMS4 as their formation is reduced in *mms21-11 mms4-mn* (Figure 4J,K) but not in *MMS21 mms4-mn*. Thus, these recombination products form in *mms21-11* from the resolution of rogue dHJs by Mus81-Mms4, implying that a significant fraction of ZMM and SDSA breaks in *mms21-11* switch to a rogue JM fate.

In summary, the evidence indicates that the Mms21 SUMO E3 ligase is required to prevent the formation of inappropriate HJ intermediates. However, in contrast to the *smc6-56* mutant, the aberrant JMs can still be removed in *mms21-11*, but require for this the activity of the Mus81-Mms4 resolvase.

### The Smc5/6-Mms21 complex is required for the function of the “rogue JM resolvases”

Despite the failure to resolve a considerable amount of JMs (Figure 3B–D), the levels and kinetics of meiotic recombination products in the *smc6-56* mutant are only marginally affected. Consequently, the Smc5/6-Mms21 complex is not essential for the resolution of all JMs in budding yeast meiosis. However, as the *mms21-11* mutant does generate aberrant HJ intermediates that need Mus81-Mms4 for resolution, the Smc5/6-Mms21 complex could be required exclusively for the removal of the ZMM-independent “rogue JMs”.

To specifically address the ability of the *smc6-56* mutant to remove ZMM-independent JMs, JM resolution and product formation was analyzed in the background of an *SGS1* meiotic null allele (*sgs1-mn*). In *sgs1-mn* mutants, nearly all ZMM recombination intermediates adopt a rogue JM fate and, consequently, nearly all recombination products, namely COs and NCOs, depend on the “rogue JM resolvases” Mus81-Mms4, Slx1-Slx4, and Yen1 [5], [9]. If the Smc5/6-Mms21 complex were critically required for the removal of rogue JMs via these resolvases, the *smc6-56* mutant should strongly inhibit both JM resolution and product formation in the *sgs1-mn* background.

**Figure 5:**
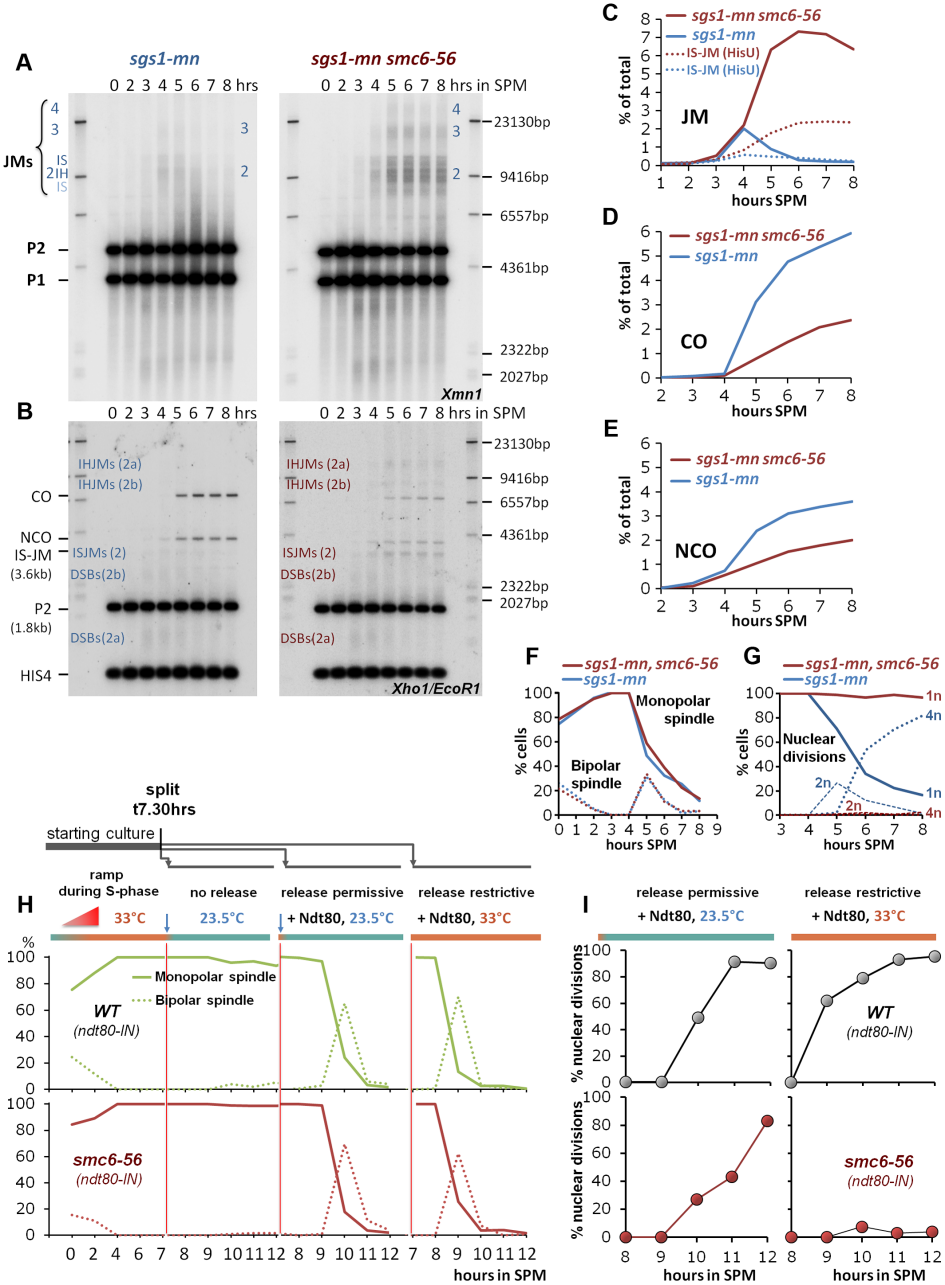
The Smc5/6-Mms21 complex is required for the function of the “rogue JM resolvases”. (A) Rogue JMs forming in *sgs1-mn* require Smc6 for their resolution: Same JM assay as described in Figure 3 (A, B). Southern blotting for JMs from synchronous meiotic time courses at 33°C. Xmn1 digest, JM detection. Left: *sgs1-mn*, Right: *sgs1-mn smc6-56*. (B) CO and NCO products from *sgs1-mn* are severely repressed in the absence of Smc6: Same CO and NCO assay as described in Figure 3 (C, D). Southern blotting of samples from synchronous meiotic time courses at 33°C. Xho1/EcoR1 digests, IS-JM/CO/NCO detection. Left: *sgs1-mn*, Right: *sgs1-mn smc6-56*. (C) Total JM signals were quantified, subtracted from background and plotted as % of total signal as a function of time in SPM. Continuous lines: JM from (A). Dotted lines: Inter sister JM from (B). Blue: *sgs1-mn*, red: *sgs1-mn smc6-56*. (D) CO signals from (B) were quantified, subtracted from background and plotted as % of total signal as a function of time in SPM. Blue: *sgs1-mn*, red: *sgs1-mn smc6-56*. (E) NCO signals from (B) were quantified, subtracted from background and plotted as % of total signal as a function of time in SPM. Blue: *sgs1-mn*, red: *sgs1-mn smc6-56*. (F) Meiotic progression is unaffected in *sgs1-mn smc6-56*: Meiotic progression was followed by spindle labeling. n = 200 cells per experiment, continuous lines: monopolar spindles, dotted lines: bipolar spindles. Blue: *sgs1-mn*, red: *sgs1-mn smc6-56*. (G) Meiotic nuclear divisions are blocked in *sgs1-mn smc6-56* double mutants. Continuous lines: cells containing one DAPI stained nucleus (1n), dashed lines: cells with 2 nuclei, dotted lines: cells with 4 nuclei (4n). Blue: *sgs1-mn*, red: *sgs1-mn smc6-56*. (H, I) Shift to permissive temperature during *ndt80* release restores nuclear divisions in *smc6-56*. (H) Upper panels, green: Wild type, lower panels, red: *smc6-56*. Continuous lines: Monopolar spindles. Dotted lines: bipolar spindles. From left to right: 1^st^ panel: Time course experiment under restrictive conditions in the *ndt80-IN* strain background in the absence of inducer (estradiol). 2^nd^ panel: shift to permissive temperature (23.5°C), without release (− estradiol). 3^rd^ panel: release (+ estradiol) into permissive temperature (causes short and synchronous burst of bipolar spindles). 4^th^ panel: release (+ estradiol) into restrictive temperature (causes short and synchronous burst of bipolar spindles). (I) % Multi nuclear cells (2n+4n) are plotted against hours in SPM. Upper panels (grey filled circles): *SMC6 ndt80-IN*, lower panels (red filled circles): *smc6-56 ndt80-IN*. Left panels: *ndt80* release (+ estradiol) into permissive conditions restores divisions in the *smc6-56* background. Right panel: release (+ estradiol) into restrictive temperature maintains the block of division.

As shown in Figure 5A–E, the *sgs1-mn smc6-56* double mutant does indeed dramatically accumulate JMs, accompanied by a substantial loss in recombination products consistent with a failure of the rogue JM resolvases in non-ZMM JM removal. In contrast, the *sgs1-mn* single mutant efficiently resolves JMs to CO and NCO products (Figure 5A–E), as expected [5], [9], [10]. Similarly to the *smc6-56* single mutant, nuclear divisions are completely blocked in the double mutant, while meiotic progression based on spindle morphology is unaffected in single and double mutants (Figure 5F,G).

In the *sgs1-mn* background where nearly all DSBs adopt a rogue fate, the amount of persistent JMs in the *sgs1-mn smc6-56* mutant is 3 to 4-fold elevated compared to the *smc6-56* single mutant, reaching a high level of 6–7% unresolved JMs. The failure to resolve JMs in the *sgs1-mn smc6-56* double mutant resulted in corresponding depletion of 60% of the *sgs1-mn* COs and 45% of the *sgs1-mn* NCOs (Figure 5C–E). We conclude that the Smc5/6-Mms21 complex is specifically required for the resolution of the unregulated, “rogue” JMs and that the resolution activity lost due to *smc6-56* equals the effect of loss of at least the most active rogue JM resolvase Mus81-Mms4 [9], [10].

To investigate whether JMs might become terminally inaccessible to resolvases due to an early defect in *smc6-56*, cells were allowed for 7 h 30 min to complete prophase I until the arrest in late pachytene of *ndt80* under restrictive temperature. Cells were then released into permissive temperature to supply functional Smc6 for JM resolution. The release was mediated by addition of estradiol using an estradiol inducible *NDT80* allele (*ndt80-IN*). Ndt80 expression induces pachytene exit and Cdc5 expression which in turn activates the resolvases for resolution of dHJs [6], [7], [62]. If JMs had derailed and become unresolvable early, the late addition of Smc6 should not be able to support nuclear divisions.

The key result of the experiment is shown in Figure 5I, namely that providing Smc6 after the *ndt80* arrest restores nuclear divisions to wild type levels, with only a slight delay, required to resolve the accumulated JMs. Notably, all the controls were as expected, that is cells efficiently resumed meiotic progression upon estradiol addition independent of the temperature (Figure 5H). Also, *SMC6* performed nuclear divisions independent of the restrictive conditions, while of course successful nuclear divisions in *smc6-56* depended on the termination of restrictive conditions (Figure 5H,I). We conclude that it is sufficient to provide Smc6 function after the *ndt80* arrest point to ensure chromosome segregation. The converse experiment, in which cells process DSBs under permissive conditions but are released under restrictive conditions from the ndt80 arrest, shows that cells block (Figure S6) consistent with a critical role of Smc5/6-Mms21 complex in mediating the function of rogue JM resolvases beyond only Mus81-Mms4.

In summary, we conclude that Smc5/6-Mms21 promotes the function of the “rogue JM resolvases” at the time of resolution - and thus rather directly, and that the integrity and accessibility of the aberrantly formed JMs is not affected.

### Smc5/6-Mms21 supports the function of the anti-recombinogenic helicase BLM/Sgs1

The biological function of the Mms21 SUMO E3 ligase domain is presumably mediated through regulation of downstream factors. The most obvious candidates for destabilizing early intermediates are helicases that can unwind recombinogenic structures. The helicase reported to interact with the Smc5/6-Mms21 complex is Mph1, an anti-recombinogenic, FANCM like helicase, but unlike Sgs1 or Mms21 SUMO ligase activity, Mph1 is dispensable for coping with the bulk of induced lesions [63], [64]. However, absence of Mph1 activity partially alleviates defects in Mms21 SUMO E3 ligase mutants, suggesting dysregulation of Mph1 activity [63], [64]. The same study also found that this suppression depends on the activity of Sgs1, the *sgs1Δ* mutant being epistatic. These data suggest that Mph1 activity becomes toxic in the absence of Smc5/6-Mms21 and may hamper Sgs1 mediated repair. It is therefore possible that the Mms21 SUMO E3 ligase mediates its anti-JM formation activity by coordinating the two helicases, Sgs1 and Mph1. However, observations in a previous study in which *mms21-11* and *sgs1Δ* conferred a synthetic growth defect in the double mutant led to the notion that Sgs1 and Smc5/6-Mms21 may not work together [64].

**Figure 6:**
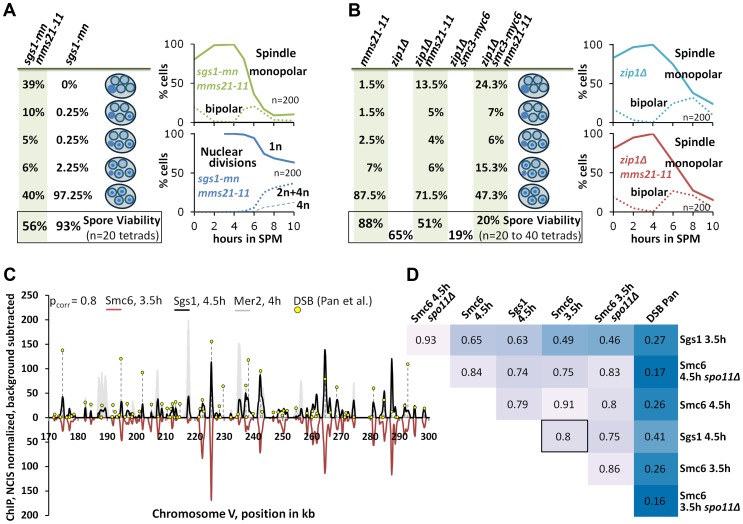
The Smc5/6-Mms21 complex supports the anti-recombinogenic function of Sgs1. (A) Lack of the Mms21 SUMO ligase mildly compromises chromatin separation in the *sgs1-mn* background. Left column on green background: From top down: Percent of tetrads with 0, 1,2,3,4 spores containing nuclear DNA. Bottom: Spore viability from tetrad dissection. Right upper panel: Meiotic progression of *sgs1-mn mms21-11* was followed by spindle labeling. n = 200 cells, continuous line: monopolar spindles, dotted line: bipolar spindles. Right lower panel: Continuous line: cells containing one DAPI stained nucleus (1n), dotted lines: cells with 2 or 4 nuclei (2n+4n), dashed lines: cells with 4 nuclei (4n). (B) As in (A). The Mms21 SUMO ligase guarantees complete chromatin separation in the *zip1Δ* mutant indicating that the Mms21 SUMO ligase is required for full Smc5/6-Mms21 resolvase activity. Top to bottom: Percent of tetrads with 0,1,2,3,4 spores containing nuclear DNA. Number at the bottom: Spore viability from tetrad dissection given as percentage. Genotypes indicated above each column. Right upper panel: Meiotic progression of *zip1Δ* was followed by spindle labeling. n = 200 cells, continuous line: monopolar spindles, dotted line: bipolar spindles. Right lower panel: Meiotic progression of *zip1Δ mms21-11* was followed by spindle labeling. n = 200 cells, continuous line: monopolar spindles, dotted line: bipolar spindles. (C; and D) Chromosomal interaction sites and intensities are highly similar between Sgs1 and Smc6. DNA-interaction sites for Sgs1-myc18 (t = 4.5 hours in SPM) and Smc6-myc13 (t = 3.5 hours in SPM) on a 130 kb region of chromosome V. ChIP-seq signals are shown after smoothing (bandwidth: 500 bp), NCIS normalization and background subtraction as described in Materials and Methods. Black: Sgs1. Smc6 signals were plotted in red on the negative scale to facilitate comparison. The obvious Sgs1/Smc6 symmetry in the example region is corroborated genome-wide by the high Pearson correlation (pcorr = 0.8) over more than 6000 peaks per profile. Yellow lollipops: DSB positions as defined in [53] (corresponding to ∼7000 DSB-hotspots across the genome). These positions map overwhelmingly often precisely at the ChIP-seq peaks. The filled grey profile represents Mer2-HAint. (t = 4h) to illustrate core site signals. Note that smaller Sgs1 signals, and to a lesser extent also Smc6 signals also overlap with core sites, as defined by Mer2. (D) Pearson correlation coefficient matrix between the peaks of all profiles shown in this work. Matching peaks (after smoothing to a bandwidth of 300 bp and subtracting the smoothed untagged control) were identified (based on exceeding a certain threshold, set here to Q = 0.7) and their intensities compared by Pearson correlation. The coloring should facilitate comparisons: dark blue represents low correlations whereas lighter shades indicate increased correlation. Intensities between DSBs and ChIP-seq profiles do not match (p_corr_<.3), whereas intensities of peaks between Sgs1 and Smc6 profiles usually highly (frequently p_corr_>.75).

In this study a striking overlap between the biological functions of Mms21 and Sgs1 is apparent. Similarly to Sgs1, Mms21 prevents the formation of rogue JMs and is required for SDSA and the repair block of early ZMM breaks, although the defect of *mms21-11* is clearly less pronounced than that of an *sgs1* mutant. This similarity in behavior of *mms21-11* and *sgs1* mutants is also observed in studies on mitotic cells [40]. In vegetative growth, we found very similar synthetic interactions for *mms21-11* as for *sgs1Δ* and *mph1Δ* (Figure S7B, C; and [65]), including reduced growth for the *mms21-11 sgs1Δ* double mutant. For the synthetic interaction of *mms21-11* and *sgs1-mn* in meiosis, we observed a moderate chromosome segregation defect in the double mutant. Specifically, 60% of tetrads were missing DNA in at least one spore and 39% of the tetrads (n = 200) were devoid of DNA in all four spores (Figure 6A).

Since Mms21 is an essential subunit of the Smc5/6-Mms21 complex and because the Smc5/6 complex itself is targeted by Mms21 for SUMOylation [27], [28] we tested whether the *mms21-11* allele might confer a mild defect in the Smc5/6-Mms21 rogue JM resolvase activity which could explain a synthetic interaction between *mms21-11* and *sgs1*. Since the JMs of ZMM mutants also depend on the rogue JM resolvases for their resolution [9], we tested whether *mms21-11* is permissive for DNA segregation in a *zip1Δ* mutant meiosis. *zip1Δ* is the most permissive ZMM mutant (presumably providing no substantial Sgs1 mediated ZMM-DSB repair block), forming JMs readily from its DSBs and consequently exhibiting a very short prophase 1 delay [4]. Accordingly, while DSB turnover and meiotic progression in the *zip3Δ* mutant could be enhanced by *mms21-11*, the already swift exit of *zip1Δ* from prophase 1 in SK1 is not improved further by *mms21-11* (Figure 6B). However, chromatin segregation is notably affected in the *zip1Δ mms21-11* double mutant with 28.5% of the tetrads missing DNA in at least one spore and 13.5% of the tetrads being completely devoid of DNA in all four spores (n = 200). Consistent with a reduced number of crossovers, spore viability is decreased from 65% of *zip1Δ* to 51.25% in the double mutant (n = 80 spores of 20 tetrads). Combination with a hypomorphic *smc3-myc6* allele, which decreases the viability of *zip1Δ* to 20.0% (n = 160 spores of 40 tetrads), even exaggerates this segregation defect to 52.7% of the tetrads lacking DNA in at least one spore and 24.3% of the tetrads (n = 300) devoid of DNA in all spores in the resulting *zip1Δ smc3-myc6 mms21-11* triple mutant. We conclude that *mms21-11* indeed mediates a defect to the rogue JM resolvase function of Smc5/6-Mms21.

Given that already low amounts of unresolved JMs completely block nuclear divisions and that *sgs1-mn* imposes a rogue JM fate on basically all recombination events [9], the defect in the rogue JM resolvase function of *mms21-11*, although relevant, must be rather weak.

Finally, we tested by ChIP-Seq if the localization pattern of the epitope tagged Sgs1-myc18 would lend support to the idea of cooperation between Sgs1 and the Smc5/6-Mms21 complex. Indeed, 70% of the 1000 strongest Sgs1-myc18 peaks map precisely to sites of meiotic DSB formation, as identified by Pan and coworkers [53] at near single nucleotide resolution where they overlap nearly perfectly with peaks of the Smc6 profiles (Figure 6C). However, the intensity of hotspot-matching Sgs1 peaks is not proportional to the activity of corresponding hotspots but proportional to the corresponding Smc6 peaks. Thus the correlation coefficients between the peaks of Sgs1 profiles and their matching DSB sites is low (cor = .27 [Sgs1, 3.5h], cor = .41 [Sgs1, 4.5h], Figure 6D). Similarly, Smc6 peaks don't correlate well with DSB intensities (cor = .26 [Smc6, 3.5h and Smc6, 4.5h]) despite localizing precisely at hotspots (Figure 6D) for most peaks. This suggests that the proteins are not recruited proportionally to the DSB activity. In contrast, peak intensities match very well between Sgs1 and Smc6 (cor = ∼.8 for different profile comparisons, Figure 6D). Smc6 and Sgs1 also match on many non-DSB positions including prominent peaks at the centromeres (figure 2G, S4, S7A). Sgs1, and to a lesser extent Smc6 also bind to core sites, but in general these signals are much smaller than their signals at DSB sites. These findings show that Smc6 and Sgs1 populate largely the same chromosomal target sites at high resolution and suggest that they are accumulated by some common characteristics at these sites that is different from actual hotspot activity.

## Discussion

### The Smc5/6-Mms21 complex antagonizes unregulated JMs

Broken chromosomes pose a considerable threat for cells; unrepaired, they cause potentially lethal loss of genetic information, but their repair is equally risky. Inappropriate recombination intermediates and outcomes are inevitable if repair is not carefully controlled. JMs typically arise as HR intermediates with both ends of the DSB lesion engaged with one or more repair templates. Having eliminated the primary lesion and any major stretches of single stranded DNA, JMs do not trigger a DNA damage checkpoint response. Thus, if JMs arise between different chromosomes they mediate dangerous, stable connections due to reciprocal base pairing and catenation which will cause chromosome non-disjunction if not removed in time before anaphase. JMs generated at non-allelic positions are particularly dangerous because their resolution can lead to deletions or translocations and thus need to be avoided in the first place. In many organisms, HR via JMs is strongly down regulated for much of their life cycle, the exception being meiosis where the generation of crossovers via JMs is imperative. Mechanisms and factors that mediate surveillance of such dangerous intermediates or the coordination of the known JM-antagonists are not well known or understood to date.

In meiosis, cells are forced to generate high numbers of crossovers and consequently evolved a specialized recombination pathway on top of the mitotic machinery to do so safely. In budding yeast, the conserved ZMM recombination pathway was first described to perform this task [4], [66], [67]. It involves a sophisticated machinery which works on a subset of DSBs (ZMM-DSBs) to generate appropriate amounts of stable JMs to achieve at least one obligatory CO per bivalent, and it also ensures that COs are formed between the appropriate partners. The ZMM-dHJs are transformed into COs at pachytene exit by a dedicated ZMM resolution machinery, dependent on Exo1-Mlh1/3 [5]. In contrast, non-ZMM DSBs are repaired fast and safely by SDSA to yield NCOs and are thought to support homology search. Recently, BLM/Sgs1 helicase was identified as being central to SDSA mediated NCO formation, as well as in preserving early ZMM recombination intermediates [10]–[12], [61]. Unregulated or “rogue” JMs that arise from non-ZMM DSBs by escaping destabilization through Sgs1, or that escape the ZMM pathway, are resolved by a group of rogue JM resolvases: Mus81-Mms4, Slx1-Slx4 and Yen1 [5], [9].

Here we demonstrate that the Smc5/6-Mms21 complex specifically binds to sites of meiotic DSBs and plays a dual role in homologous recombination as an antagonist of unregulated JMs and HJ intermediates. It does so by promoting anti-JM formation activity through its Mms21 SUMO E3 ligase and, if inappropriate JMs have already formed, by mediating their resolution through rogue JM resolvases. Similar conclusions have been drawn in an independent study (accompanying manuscript) [54]. In particular, that study showed directly that Exo1-Mlh1/3 dependent resolution of ZMM-dHJs does not require Smc5/6-Mms21.

Phenotypes associated with defects in these functions include the recombination dependent failure to separate chromatin during meiosis upon Smc6 inactivation and the concomitant accumulation of persistent JMs that also include a considerable fraction of unresolved IS-JMs and some three-strand JMs indicative of aberrant JM formation. This phenotype resembles the behavior of *sgs1-mn mms4-mn* mutants [10], [12] in which most meiotic recombination events give rise to “rogue” non-ZMM JMs and block nuclear divisions, because they depend on the inactivated “rogue JM resolvase” Mus81-Mms4 for resolution. In analogy, this implies that the Smc5/6-Mms21 complex mediates both functions in the management of unregulated JMs. It supports the prevention of rogue JMs as does Blm/Sgs1 and mediates their efficient removal through the rogue JM resolvases. Early defects of Smc5/6-Mms21 mutants were also noted in the accompanying manuscript by Copsey and coworkers [54], who observed an increase in IS JMs, as well as an increase of Zip3 foci indicating compensation for derailed ZMM intermediates, and high levels of unresolved JMs.

This view is corroborated by the role of the SUMO E3 ligase domain of Mms21. *mms21-11*, which lacks this domain, largely separates the two functions of the complex. This allele is defective in the anti rogue JM formation activity of the complex but only mildly affects Smc5/6-Mms21 resolution function. In the *mms21-11* mutant, a significant amount of additional JMs are formed and in the background of the meiotic null allele *mms4-mn*, unresolved JMs accumulate comparable to the *smc6-56* mutant (Figure 3B,E, 4E,I). Thus, the SUMO E3 ligase domain of Mms21 is required for the anti rogue JM formation activity of the complex but is largely dispensable for the resolution activity.

**Figure 7:**
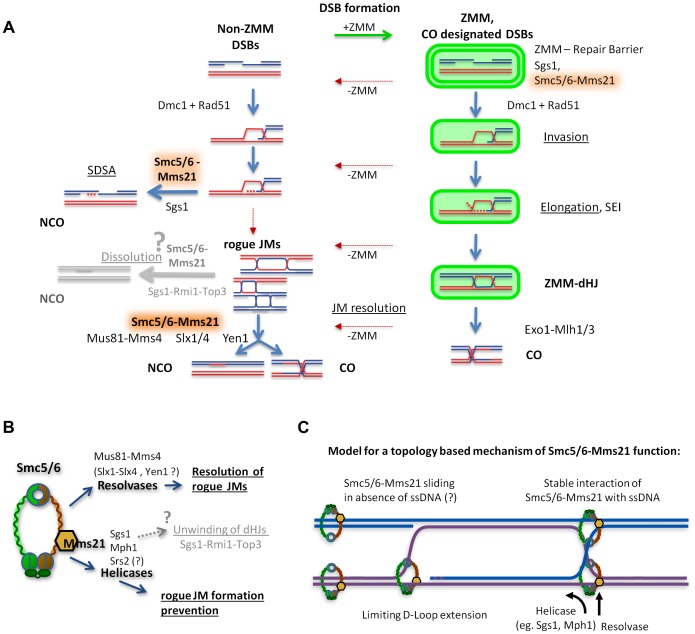
Model of how Smc5/6-Mms21 may antagonize inappropriate JMs in meiotic recombination. (A) A representation of homologous recombination and intermediate pathway choice in the course of budding yeast meiosis is shown, along with the points of function for Smc5/6-Mms21 in antagonizing inappropriate JMs. Meiotic DSBs can engage for two alternative fates: non-ZMM DSBs and ZMM-DSBs (highlighted in green). Regular pathway progression is marked with blue arrows and aberrant deviations are depicted in red. By default, stable strand invasions are disassembled for all breaks through the action of Sgs1 helicase and Smc5/6-Mms21. Non-ZMM DSBs are consequently repaired by SDSA into non-crossovers. In contrast, ZMM-DSBs are in addition also prevented from being repaired at all until licensed in the ZMM pathway for progression to stable invasions, here referred to as “ZMM-Repair Barrier”. When a ZMM-DSB is allowed to form stable SEIs and dHJs, the ZMM-pathway protects that DSB from the action of Sgs1 and Smc5/6-Mms21 and marks the resulting dHJ for CO specific resolution by Exo1-Mlh1/3. Intermediates that deviate from their supposed repair pathways by inappropriate intermediate stabilization and absence of a ZMM label result in “rogue” JMs that depend on Smc5/6-Mms21 for resolution by the rogue JM resolvase Mus81-Mms4 (and probably also Slx1-Slx4 and Yen1). (B) Our data implicates the Smc5/6-Mms21 complex in at least two independent mechanisms for rogue JM avoidance: On one hand prevention of JM formation through Mms21 SUMO E3 mediated regulation of anti-recombinogenic helicases, and on the other hand, the promotion of resolution of JMs by rogue JM resolvases. We also assume that through regulation of helicases, the Smc5/6-Mms21 complex may very likely also be involved in regulating dHJ dissolution by Sgs1-Rmi1-Top3. (C) Smc5/6-Mms21 may survey the DNA for displaced ssDNA at D-loops or HJs by topologically entrapping dsDNA as a sliding SMC ring and binding stably to ssDNA (e.g. at the hinge or any other part of the ring) upon encounter. Stable binding to such ssDNA will constrain D-loop extension and HJ branch migration and label sites with a ssDNA/dsDNA interface where appropriate action of anti-recombinogenic helicases (like Sgs1 or Mph1) and resolvases will be required.

On the other hand, inactivation of Smc6 severely compromised JM resolution and recombination product formation in an *sgs1-mn* mutant background (Figure 5A–E, see also accompanying manuscript for a mutant in another Smc5/6-Mms21 subunit, Nse4 [54]), where JM resolution is almost fully dependent on the rogue JM resolvases [5], [9]. The resolution deficiency in *sgs1-mn smc6-56* equals or exceeds that seen in *sgs1-mn mms4-mn* [9], [10], thus resolution activity equivalent to at least the Mus81-Mms4 resolvase must have been lost (identical conclusion in the accompanying study [54]). Providing Smc5/6-Mms21 function after the Ndt80-IN pachytene arrest proves sufficient to ensure meiotic chromosome segregation. This indicates that the lack of early (destabilizing) function produced no irreversible damage and can be compensated for by the late (resolution) function. This also suggests that late recruitment of the complex to promote resolution is possible. In this way, the Smc5/6-Mms21 complex supports meiotic recombination pathway choice and safeguards the turnover of non-ZMM intermediates, particularly the otherwise unregulated non-ZMM JMs (Figure 7A). These observations imply that the Smc5/6-Mms21 complex recognizes recombinogenic lesions and locally mediates antagonistic activities. How might the Smc5/6-Mms21 complex mediate this activity?

### The Smc5/6-Mms21 complex affects the activity of anti-JM factors

The Smc5/6-Mms21 complex binds to DSB sites, suggesting it might locally recruit and/or orchestrate the function of anti-recombinogenic helicases and of resolvases. The absence of the complex in meiosis renders the activity of Sgs1 insufficient for normal intermediate destabilization and protection and strongly impairs the function of the rogue JM resolvases, supporting the above model. Notably, the defect of *smc6-56* and *mms21-11* mutants on rogue JM prevention is weaker than that of *sgs1-mn* leaving the ZMM pathway largely operative and thus CO formation unaffected by Smc6 inactivation. The phenotype of the SUMO E3 domain deletion suggests that Smc5/6-Mms21 mediates its anti-rogue JM formation activity through SUMOylation dependent regulation and coordination of anti-recombinogenic helicase activity at the site of the lesion. Sgs1 and Mph1 are the most likely targets. Interestingly, it has been reported that the FANCM-like helicase Mph1 becomes toxic in the absence of the Mms21 ligase activity [63]. The observed auto-SUMOylation of the complex could also be critical in this function for recruiting or sequestering regulatory targets [27]. In addition, since DNA damage induced SUMOylation of Sgs1 has been reported [40], Sgs1 may represent a direct substrate for post translational regulation by Smc5/6-Mms21. However, it was also reported that Mms21 is not essential for this SUMOylation and that different SUMO E3 ligases may provide redundancy [40], [68]. Consistent with a regulatory role of the Smc5/6-Mms21 complex for anti-recombinogenic helicase function, we found that the recruitment of Sgs1 to meiotic DSBs does require neither Mms21 SUMO E3 ligase activity nor an intact complex since Sgs1-myc18 enrichment at DSB hotspots comparable to wild-type is detected by qChIP in both *mms21-11* and *smc6-56* mutants (Figure S8A,B).

Beyond its supportive role in the prevention of rogue JMs, we find a profound defect of *smc6-56* in resolution of rogue JMs in meiosis, implying a direct function of Smc5/6-Mms21 in promoting rogue JM resolvase activity. There are three rogue JM resolvases identified in *S. cerevisiae*, Mus81-Mms4, Slx1-Slx4, and Yen1.

Mus81-Mms4 is the one resolvase which facilitates the removal of the bulk of rogue JMs arising from homologous recombination [5], [9]. Comparing our results to published data, we estimate that in the *smc6-56* mutant resolution activity equivalent to at least the Mus81-Mms4 resolvase must have been lost [9], [10]. There is no evidence that Mus81-Mms4 is subject to SUMOylation or direct interaction with Smc5/6. However, the complex may stabilize the HJ and present the substrate DNA to Mus81-Mms4, or it may mediate processing steps preceding resolution. With the Smc5/6-Mms21 complex, we could identify the second known factor critical for full activity of the Mus81-Mms4 resolvase after the identification of its activating kinase Cdc5 [7].

In contrast to Mus81-Mms4, direct interaction of the Slx1-Slx4 resolvase with the Smc5/6-Mms21 binding partner Rtt107 was observed, although it is unclear whether Rtt107 binds Slx1-Slx4 and Smc5/6-Mms21 alternatively or simultaneously [38]. Slx4 is also a SUMO substrate and interacts with Rad1-Rad10, a ssDNA nuclease involved in DNA processing during repair [69], [70]. Therefore, direct regulation of Slx1-Slx4 and Slx4-Rad1-Rad10 via Smc5/6-Mms21 is possible. However, during meiosis, Slx1-Slx4 only plays a minor role for rogue JM resolution, and also Rad1 did not appear to be required for JM resolution [5], [9]. Yen1 is the third rogue JM resolvase in budding yeast. Biochemical studies indicate that HJs are its natural substrate without the need for prior processing [71]. Yen1 becomes active around the onset of metaphase 2 and will resolve most rogue JMs still present, even in the absence of Mus81-Mms4 and Slx1-Slx4 [5], [7], [9] but not at elevated JM levels as in an *sgs1* background. *smc6-56* may represent a strong hypomorph for one, two or all rogue JM resolvases, however, the strong JM accumulation seen in *sgs1-mn smc6-56* suggests that more than just the function of Mus81-Mms4 is affected.

Intriguingly, we find preloading of Smc6 to break sites. This could be a reasonable safety measure when anticipating the programmed generation of hundreds of DSBs. It was reported recently that Rtt107 promotes recruitment of Smc5/6-Mms21 specifically to the HO site upon DSB formation [38]. However, as Rtt107 is neither essential (like Smc5/6) nor required for Smc5/6-Mms21 function, Rtt107 may rather enhance the recruitment of Smc5/6-Mms21 to specific loci in anticipation of DNA damage. Meiotic DSB-hotspots are located in promoters and nuclease sensitive DNA regions [72]–[74] and are frequently associated with chromatin features such as H3K4me3 [75] that may directly, or via Rtt107, allow for local enrichment of Smc5/6-Mms21 to protect vulnerable DNA regions.

### A topological model for the function of the Smc5/6-Mms21 complex

Smc5/6-Mms21 represents a ring shaped SMC complex, highly similar to its closely related brethren, cohesin and condensin [28] for which topological mechanisms of function were successfully demonstrated [23], [76]. While evidence for topological binding of the Smc5/6-Mms21 complex to DNA is still missing to date, its overall high similarity to cohesin and condensin and also previously proposed DNA damage independent loading through the cohesin loaders Scc2-Scc4 to chromatin [39], make a topological component in the function of Smc5/6-Mms21 likely.

If the Smc5/6-Mms21 complex would serve solely as a platform for JM antagonizing factors and their regulators, it would appear inconceivable why the SMC ring should have been maintained throughout evolution. Instead, it is far more likely that a preceding topological function of the evolutionary ancestor SMC complex fulfilled a function rudimentarily similar to Smc5/6-Mms21, which was ultimately enhanced in the course of evolution. In addition, preemptive loading of Smc5/6-Mms21 to DNA would appear considerably more effective in preventing dangerous lesions if the Smc5/6-Mms21 complex were not statically bound but rather could slide along longer regions of DNA, as proposed for cohesin [77].

How could the Smc5/6-Mms21 complex mediate its function in antagonizing JMs through an underlying topological association with DNA? We propose the following model to explain the previously observed functions for the Smc5/6-Mms21 complex.

The Smc5/6-Mms21 ring, preemptive of DNA damage, is loaded onto dsDNA, entrapping (in contrast to cohesin) only one dsDNA molecule. Such topological loading onto a single dsDNA molecule and the previously reported strong ssDNA binding properties of Smc5 and Smc6 [25], [26] are sufficient to counteract and stabilize recombination intermediates, and can serve to direct the activity of anti-recombinogenic helicases as well as resolvases at (and to) the according site, namely, the junction between the HR partners (Figure 7C, S9A).

In such a model, Smc5/6-Mms21 could slide freely along the intact dsDNA until encountering the ssDNA/dsDNA interface of an occurring lesion. Such an interface would be present at a progressing D-Loop, or at the HJ of the mature intermediate. Stable binding of the Smc5/6-Mms21 ring at such ssDNA junction sites may limit the spatial freedom for subsequent D-Loop extension, reduce the chance for second end capture, and impede further branch migration of HJs towards detrimental dHJ extension (as such expanding branch migration would require to overcome one obstructing strand of the HJ topologically) (Figure 7C, S9A). We also believe that this model could be extended to mitosis and be applied to recombinogenic structures such as cruciform DNA structures arising from replication fork regression.

By these means, the Smc5/6-Mms21 complex could survey for recombinogenic lesions, stabilize them through binding and, ultimately, employ counteractive measures by means of helicases and resolvases. As a corollary, this model postulates that Smc5/6-Mms21 mediates its function from outside the lesion, with involvement from only the intact donor DNA molecule being sufficient, to ultimately associate with the very borders of the recombinogenic lesion. Our observations that Smc5/6-Mms21 specifically enriches to sites of DSB formation after break formation but, nonetheless, fails to reveal significant on-top co-localization with the recombinosome marker Rad51, are consistent with this supposition.

In addition, it is to date unknown how anti-recombinogenic helicases like Sgs1-Rmi1-Top3 or Mph1 are directed to mediate their function in the context of a lesion. Association of a helicase to the wrong strand of the recombination junction will, instead of disassembly, result in extension. This problem is also eminent in the function of the Sgs1-Rmi1-Top3 dHJ dissolvase. While hetero-duplex DNA may provide information about the relative position of the parent molecules, it cannot account for directing the dissolution of JMs between perfectly identical sister-chromatids. Since in our model Smc5/6-Mms21 would inherently mark the parent associated “outsides” of a lesion, it may thus provide the lesion with a polarity and direct the anti-recombinogenic helicases for acting in the appropriate orientation (Figure S9B).

By these means, the Smc5/6-Mms21 complex could stably mark recombination intermediates and thus orchestrate the activity of anti-recombinogenic helicases and resolvases at the very site where JM antagonizing effectors are needed. While downstream effectors of the complex may vary to suit the specific needs of the individual organism or cell type, we postulate that the underlying JM restraining activity of Smc5/6-Mms21 complex is conserved throughout organisms.

## Materials and Methods

### Yeast strains

All strains used in this study are derivatives of SK1. Detailed genotypes are provided in Table S1. Strains were constructed by crossing or LiAc transformation using standard procedures. The *URA3-arg4* recombination reporter was described in [47]. In the meiotic null mutants *mms4-mn*, *sgs1-mn* and *nse4-mn* the respective promoters are replaced by a *CLB2* promoter fragment [78] and the estrogen inducible *ndt80-IN* system has been described [79].

### Growth conditions and synchronized sporulation

Yeast strains were grown at 30°C in supplemented YPD (1% Difco yeast extract, 2% Difco peptone, 2% dextrose, 75 mg/L Ade, 75 mg/L Ura, 75 mg/L Trp) with exception of the *smc6-56* mutants which were grown at 23.5°C permissive temperature. All manipulation of strains followed standard procedures. For synchronous sporulation in liquid culture, strains were grown at 30°C overnight in SPS pre-sporulation media (0.5% Difco yeast extract, 1% Difco peptone, 0.17% Difco yeast nitrogen base w/o AA&AS, 1% potassium acetate, 0.5% ammonium sulphate, 0.05 M potassium-biphthalate, pH 5.5) to an OD660 of 1.1–1.3 (4×10^7^ cells/ml). Meiosis was induced by a subsequent wash and transfer of the cells into pre-warmed supplemented SPM (1% potassium acetate, 0.001% PPG2000, 4 mg/L Ura, 4 mg/L Trp, 4 mg/L His, 4 mg/L Arg, 6 mg/L Leu) at equal volume. Maximum aeration was provided for efficient meiosis. For synchronous meiosis, *smc6-56* mutants were allowed to exit mitosis and proceed through most of pre-meiotic S-phase under (semi-) permissive conditions with the following temperature regime applied (after pre-growth at 23.5°C): At 0 hr SPM, upshift to 26°C, at 1 hr15 min to 28°C, at 1 hr40 min to 30°C, at 2 hr20 min to 33°C. Every temperature shift is translated to the culture media within 10 minutes. We find that *smc6-56* is just able to perform nuclear divisions if undergoing meiosis at 30°C as long as not burdened by any additional number of rogue JMs (data not shown). Therefore, we define 28–30°C as semi-permissive temperature for *smc6-56* in meiosis. *ndt80-IN* expression and exit from *ndt80-IN* pachytene arrest was induced by the addition of β-estradiol (ED, 5 mM stock in EtOH, stored at −20°C) to a final concentration of 1 µM at 7 hr30 min SPM, at which point approximately 80–90% of possible JMs had already formed [9]. The separation of early and late functions of Smc5/6-Mms21 in the respective experiments was performed using *smc6-56* and the *ndt80-IN* system. Elimination of the early, pre pachytene-exit function and providing Smc5/6-Mms21 only after pachytene: Normal S-phase upshift to restrictive 33°C and arrest in pachytene followed at 7 hr30 min by addition of ED for Ndt80 induction and linear downshift to 23.5°C over 25 minutes. Elimination of the late, post pachytene function of Smc5/6-Mms21: Cells were transferred into SPM at 23.5°C and allowed to arrest in pachytene. The temperature was raised to 25.5°C from 6 hr45 min to 7 hrs, to 33°C until 7 hr20 min, and ED for *ndt80-IN* release was added at 7 hr30 min.

### Physical recombination intermediate assays

Preparation of genomic DNA and Southern blotting of one dimensional 0.6% agarose gels for physical detection of recombination intermediates was performed as described in detail [47], [80], [81] using the CTAB/CoHex/Mg2+ procedure which stabilizes HJ intermediates. Relevant sets of JM experiments were performed in parallel for comparability. For radioactive Southern hybridization, the ArgD probe was used for detection of both maternal and paternal *URA3-arg4EcPal*/*URA3-ARG4* loci and the HisU probe for allele specific detection of the maternal *his4::URA3-arg4EcPal* locus. Both probes are described [47]. As size marker, lambda HindIII digested DNA was used and probed against. Storage phosphor screens were used for signal detection and scanned with the Bio-Rad Molecular Imager FX. Quantification of signals was performed using Fuji Image Gauge Ver4.0. Frequencies are given as a percentage of total DNA signal.

### Cytology

Nuclear divisions were followed on ethanol fixed and DAPI stained whole cells. For spindle staining on whole yeast cells, 1 ml aliquots of culture were fixed in 3.2% formaldehyde overnight at 4°C. Cells were washed and cell walls digested at 37°C using 100 µg Zymolyase 20T (SEIKAGAKU #120491), 70 mM DTT and 1 M sorbitol, in 200 µl. Cells were mounted on poly-L-lysine coated slides and fixed for 3 minutes in ice cold methanol and 10 seconds in ice cold acetone. Cells were blocked and immunostained in 0.5% BSA fraction V and 0.2% gelatin in 1×PBS. Microtubuli were detected using rat anti-tubulin-alpha (Serotec MCA78, 1∶200) and FITC-conjugated rabbit anti-rat (Sigma F1763, 1∶100) antibodies. Vectashield with DAPI (Vector Laboratories H-1200) was used to stain the DNA and stabilize the label. Chromosome surface spreads and immunostainings were performed as described [82]. For antibodies used in this study see Supplemental Information, Table S2. Evaluation of synapsis followed the criteria used in [83]. Chromosome pairing in *zip3Δ* mutant surface spread nuclei was evaluated by counting pairs of Hop1 axes of roughly equal length and in close proximity to each other, strictly parallel to each other, or connected via axial associations, or engaged in (pseudo-) synapsis. Images were taken on a Zeiss Axioskop fluorescence microscope with a Photometrics CH250 CCD camera using IPLab Spectrum with magnification and exposure times constant.

### Flow cytometry

Flow cytometric quantification of cellular DNA content was performed with a BD Biosciences FACSCanto on ethanol fixed cells in the presence of 20 µg/ml propidium iodide in 50 mM Tris pH 7.5. Prior measurement, cells were treated overnight with 2 mg/ml RNaseA in 50 mM Tris 15 mM NaCl pH 7.5 at 40°C and one hour with 350 µg Proteinase K at 50°C in 500 µl volume. After brief sonication, propidium iodide was added 15 minutes before performing measurements. 5000 events were counted for each sample. Analysis was performed with Treestar FlowJo v10.

### ChIP-seq

ChIP from meiotic cultures was performed as described by Panizza and coworkers [51]. In brief, 4×10^9^ cells were collected per time point and incubated with para-formaldehyde (1% final concentration) for 15 minutes at 25°C. Cross-linking was stopped by addition of glycine to 131 mM. 4×10^9^ cells were divided into 8 aliquots and separately opened using a multibead shocker (YASUI-KIKAI, Osaka) at 2,500 rpm, 15 cycles of 30 sec on and 30 sec off, at 4°C. Extracts were sonicated to an average of 200 bp by a Covaris S2 instrument. After removing the cell debris, the supernatant was used for the chromatin immunoprecipitation. For each sample, 50 µl Dynabeads Pan mouse IgG (Invitrogen) were incubated with the 9E11 anti-myc (mouse) antibody for 6–15 hr at 4°C. The precipitated DNA was used as a template for quantitative real-time PCR using GoTaq qPCR Master Mix for SYBR assay (Promega) (primer sequences: DSB1: CCGCAGAAGCCAACAAACGG, CTTTCGGTGGAACCTCGACC; DSB2: CGTGCCAGATTGAATTTTGA, GAATGGCCTTGGTAGCAAAT; DSB3: ACTTCCAACTGCAGGACGAC, ATCTGGCGGATGAACTTGAG; DSB4: ACGAACAGAGTCCCGAACCT, GCGGTTAATTCGATGGAAAG; CORE1: TGGATGGCAACTGAAGGAGC, TGGAATACCTATGAGTTGACTGC; ADP1: GGTGATGATTGCTCTCTGCC, CGTCACAATTGATCCCTCCC).

For genome-wide analysis (ChIP-seq), one-tenth of the ChIP DNA was analyzed by real-time PCR (qChIP) while the remaining DNA was concentrated by precipitation in ethanol. For the preparation of libraries for Illumina sequencing, we strictly followed the protocols provided by Illumina (Illumina ChIP-seq DNA sample prep protocol). Briefly, DNA-ends were repaired to convert overhangs to phosphorylated blunt ends with T4 DNA polymerase, E. coli DNA Pol I (Klenow fragment), and T4 polynucleotide kinase (PNK). An ‘A’ nucleotide was added to the 3′ end of the blunt ended fragments using Klenow fragment (3′ to 5′ exo minus). This prepared the DNA fragments for ligation to the adapters (Truseq Adaptor1-5) which have a single ‘T’ base overhang at their 3′ ends. After ligation, excess adaptors were removed by selecting a certain size range from 200–500 bp with QIAquick Gel Extraction Kit (Qiagen). Purified templates were PCR amplified with 15–18 cycles by KAPA-HiFi Hot Start PCR Kit (KAPA biosystems). Before hybridization to the flow cell, the amount and the size of the DNA library was controlled to be at least 1 µg/ul (for an average fragment size of 300 bp).

### Processing of ChIP-seq data

Sequencing was performed at the CSF NGS Unit (csf.ac.at) with Illumina HiSeq 2000 resulting in 50 bp single-end reads in multiplex (Illumina TruSeq adapters 1–5). 2.3–8.6M reads per sample could be aligned with high confidence to the *S. cerevisiae* strain S288C, genome version R64-1-1 (20110203; http://downloads.yeastgenome.org/sequence/S288C_reference/genome_releases/). We used NextGenMap (http://cibiv.github.com/NextGenMap) for fast and sensitive alignment allowing up to 5000 hits per sequence. Read depth per position was generated in Java, dividing each hit by the number of genome-wide matches in case of multi-mapping reads.

All subsequent analyses were performed using R (version 2.15.2). After summing up the read depth of both strands, gaps were filled up with zeros followed by smoothing with ksmooth (Nadaraja-Watson kernel) with bandwidths as indicated and a resolution of 10 bp. Samples were normalized relative to their negative controls using NCIS [84]. This method estimates the fraction of background signal in each sample. After normalization, the untagged control sample was subtracted from each profile for background removal. The profiles remained robust against changes between different negative controls.

Peaks were defined as being flanked on both sides by valleys with a minimum depth. The maximal distance between matching peaks from different profiles was set to be equal to the smoothing bandwidth. Peak heights were subsequently compared by pair-wise Pearson correlation (R, cor). The significance of the number of overlapping peaks between profiles was assessed by a hyper-geometric random model. For matching peaks with DSB hotspots, peaks were required to map precisely between the borders of the DSB hotspots. The significance of the number of peaks matching to hotspot regions was assessed by a binomial random model. Here hotspots are defined according to [53] as groups of Spo11-oligo 5′-ends mapping less than 50 bp from each other. To account for the differences in genome versions, the raw data taken from [53] (GEO accession GSE26449) were re-aligned to the same genome version as the other samples, R64-1-1. Mapped sequence reads and generated profiles for this study are provided at the GEO repository, accession number GSE51977.

## Supporting Information

Figure S1 Meiosis specific depletion of Smc6 leaves meiotic progression and chromosome synapsis unaffected (Related to Figure 1). (A) A time series of FACS profiles for pre-meiotic DNA replication in the *smc6-56* mutant from indicated time points post induction of meiosis and under our regiment for gradual inactivation of Smc5/6 at late S-phase. Subsequent meiotic progression is shown for this time course experiment on meiotic spindles (n = 200 for each time point), as exit from meiotic prophase 1. (B) The semi-permissive temperature of 30°C for the *smc6-56* allele permits meiotic DNA segregation while the restrictive temperature of 33°C does not. Meiotic nuclei of ethanol fixed and DAPI stained cells were counted at the indicated time points and temperatures (n = 200). The sum of bi- and tetra-nucleated cells (2n+4n) is plotted. (C) Upper panels: Three spread nuclei representing examples of pachytene cells showing no aberrant features from *wild type*, *smc6-56* and *mms21-11* mutants as indicated. White: DAPI stained chromosomes, Green: Zip1 protein demonstrating full synapsis, Red: Few, normal residual Rad51 foci. Lower panels: Quantification of synapsis in *wild type*, *smc6-56* and *mms21-11* diploids from 4, 5 and 6 hour time points of sporulation (different shades of blue) at the temperatures indicated. All mutants form normal SC, although *mms21-11* showed a reduced yield of extended and full SCs at lower temperature.
(TIF)

Figure S2 Binding pattern of Smc6 on meiotic chromatin by cytology (related to Figure 2). (A) Smc5-HA3 and Smc6-myc13 foci colocalize: Red: Smc6-myc13, Green: Smc5-HA3, white bar: 10 µm. White rectangle indicates position of magnified sub region. Co-localization for 1689 foci of 8 prophase 1 nuclei was determined. (B) Smc6-myc13 localizes early to meiotic chromatin, concomitant with the appearance of Rec8-HA3 foci. 5 nuclei (from 2 h in SPM) represent consecutive stages from left to right, based on the abundance of Rec8-HA foci. Panels from top: Rec8-HA3, Smc6-myc13, chromatin stained with DAPI. White bar: 10 µm. Colored Panel: Overlay of Rec8 and Smc6 signals reveal very little overlap in early nuclei. Red: Smc6-myc13, Green: Rec8-HA3, White: DAPI. White rectangle indicates position of magnified sub region. Upon close inspection, none of the early Rec8 and Smc6 foci in the region show significant overlap. (C) Partial co-localization between Zip4-myc9 and Smc6-HA3. Nuclei from 4 hours in SPM show limited on top but frequent side by side localization, similar to the situation between Rad51 and Smc6. Red: Smc6-HA3, green: Zip4-myc9, white bar: 10 µm. White rectangle indicates position of magnified sub region.
(TIF)

Figure S3 Chromatin binding of Smc6 throughout meiosis by cytology (related to Figure 2). 35 spread nuclei of various meiotic stages sorted in a consecutive order from left to right and from top to bottom by combining information from Zip1 and DAPI morphologies. Each nucleus is represented by three micrographs, a green and red merge (between Zip1 and Smc6, respectively), a merge between blue and red (DAPI and Smc6) and a white micrograph (DAPI staining of DNA). The upper three rows depict stages from the earliest appearance of Smc6 representing presumably (early) replicating nuclei, to leptotene and zygotene. The three lanes in the middle represent zygotene, pachytene and post pachytene stages (presumably corresponding to diplotene). Three white arrowheads point at dense clusters of Smc6 foci accumulating at the unsynapsed regions of chromosome XII, representing the rDNA. The bottom three lanes correspond to late stages: Prometaphase/metaphase, as well as several anaphase I and anaphase II examples. Three white arrowheads point at dense clusters of Smc6 foci accumulating at the residual connecting chromatin between the almost pinched off nuclei. White bar: 10 µm.
(TIF)

Figure S4 Smc6 is recruited to meiotic DSB hotspots where it enriches to upon DSB formation (related to Figure 2D,E). ChIP-seq profiles for complete chromosomes. (A, B) The upper two panels show chromosome III, the lower two chromosome V. Of the two panels depicting the same chromosome the upper one corresponds to time point 3.5 hours in SPM, the lower one to 4.5 hours in SPM. The color coding is: Black: Smc6-myc13, blue: Smc6-myc13, *spo11Δ*, red: DSBs shown on the negative axis for better comparison. A profile of chromosome axis protein Hop1 (taken from [51]) was added in grey to mark axis sites. Appropriately colored arrows point to sites where the primers for cold region, hotspot and core used for qPCR are located. Here ChIP-seq profiles were smoothed (bw = 250 bp), decile normalized and background subtracted (except for Hop1). (C) qPCR of a biological repeat at three positions on chromosome III from the same experiment, a DSB site (ca. at 211k, YCR047C), a core site (ca. at 219k) and a cold spot (ca. at 136, ADP1). To correct for possible differences in the efficiencies of the IPs across the different strains, the enrichment of core and DSB qPCR signals relative to the ADP1 signal is plotted for the indicated genotypes and time points, Core/ADP1 shown in blue, DSB/ADP1 in red. (D) Overlap of Smc6 (4.5 h) peaks with the 1000 strongest DSB hotspots [53]. A peak is considered overlapping if it lies within the borders of the hotspot. Left column: % of DSB hotpots that are hit by Smc6 peaks. Middle column: The number of Smc6 peaks required to hit the corresponding percentage of DSB hotspots. Right column: The significance (binomial random model), which was in all cases below the smallest value displayed by R (10^−15^). Because the strongest hotspots do not always correspond to the strongest Smc6 peaks, naturally, there is only 34% overlap between the 1000 strongest Smc6 (t4.5) and DSB sites. However, nearly all of the 1000 strongest DSB hotspots contain an Smc6 peak if smaller Smc6 peaks are also considered (e.g. 80% when considering 6000 Smc6 peaks). The significance of this co-localization is still very high (p(random model)<10^−15^).
(TIF)

Figure S5 Smc5/6 mutants promote the formation of inappropriate, stable recombination intermediates (related to Figure 4). (A) *smc6-56* does not promote axis pairing in the *mnd1Δ* mutant which is defective for the DSB strand invasion step in meiotic homologous recombination. Upper panel: Quantification of bivalent formation on spread nuclei of 5 hrs SPM stained against Hop1 (n = 50 for each mutant). Number of incidences is plotted for each category of “0” to “16” bivalents. Dark blue: *mnd1*Δ, blue: *mnd1*Δ *smc6-56*, red: *zip3*Δ, green: *zip3*Δ *smc6-56* (all at 33°C). Lower panel: Representative nuclei of the indicated mutants at 5 hrs SPM after chromosome spreading and staining against Hop1 (green). (B) Inactivation of the rogue JM resolvases cannot promote stabilization of axial associations in the *zip3Δ* mutant while inactivation of Smc5/6-Mms21 does, indicative of inappropriate JM formation. As in (A): Upper panel: Quantification of bivalent formation on spread nuclei of 5 hrs SPM stained against Hop1 (n = 100 for each mutant). Blue: *zip3*Δ, red: *zip3Δ mms4-mn slx1Δ yen1Δ*, green: *zip3*Δ *nse4-mn* (at 28°C). Lower panel: Representative nuclei of the indicated mutants at 5 hrs SPM. Hop1 in green. (C) Removal of all characterized meiotic resolvase activity cannot promote chromosome pairing in *zip3Δ* such as observed in *zip3Δ sgs1-mn* and *zip3Δ nse4-mn*. Left panel: Quantification of bivalent formation on spread nuclei of 5 hrs SPM stained against Hop1, plotted as percentage of incidents (n = 150 for each mutant). Blue: *zip3*Δ, pink: *zip3Δ mms4-mn slx1Δ yen1Δ mlh3Δ*, light green: *zip3*Δ *nse4-mn*, dark green: *zip3*Δ *sgs1-mn* (at 30°C). Right panel: Representative nuclei of the indicated mutants at 5 hrs SPM. Hop1 in green.
(TIF)

Figure S6 Inactivation of Smc5/6-Mms21 at pachytene exit prevents nuclear divisions (related to Figure 5). A shift to restrictive temperature during *ndt80* release abolishes nuclear divisions in *smc6-56*, indicating that early function of Smc5/6-Mms21 is not sufficient to relieve the block of division, but that late functions are required. The experiment is related to Figure 5 (H,I), except that in this experiment the temperature regime was inverted, starting with meiosis at permissive temperature until the release from the *ndt80-IN* arrest. Left panels show meiotic progression in response to estradiol addition, as monitored by spindle staining. The permissive (23.5°C) and restrictive (33°C) temperatures are indicated by turquoise and orange colors above the bar diagram. ED indicates the timing of addition of estradiol to induced Ndt80 expression and exit from pachytene. In contrast, a shift to permissive temperature during Ndt80 release restores nuclear divisions in *smc6-56* (Figure 5I). Upper panels: Wild type (*ndt80-IN*), lower panels: *smc6-56* (*ndt80-IN*). Light green bars: Monopolar spindles, dark green bars bipolar spindles. From left to right: 1^st^ panel: Time course starting under permissive conditions in the n*dt80-IN* strain background in the absence of inducer (estradiol). 2^nd^ panel: shift to restrictive temperature (33°C), without release (− estradiol). 3^rd^ panel: release (+ estradiol) into restrictive temperature (causes short and synchronous burst of bipolar spindles). 4^rd^ panel: release (+ estradiol) of cells that started meiosis under restrictive conditions into restrictive temperature (positive control for blocked divisions, causes short and synchronous burst of bipolar spindles). The four diagrams on the right show nuclear divisions as a function of time post Ndt80 expression: % 1n (circle), 2n (triangle) and 4n (squares, n = number of nuclei) plotted against hours in SPM. Upper two panels show normal divisions in *SMC6*, independent of treatments. Lower panels: Nuclear divisions are blocked, irrespective of whether they spent their prophase (DSB repair) under restrictive or permissive conditions, although the chromatin appears more flexible during anaphases if Smc5/6-Mms21 was provided during DSB repair.
(TIF)

Figure S7 Analogies between Smc5/6-Mms21 and Sgs1 (related to Figure 6). (A) Chromosomal interaction sites and intensities are highly similar between Sgs1 and Smc6. DNA-interaction sites for Sgs1-myc18 (t = 4.5 hours in SPM) and Smc6-myc13 (t = 3.5 hours in SPM) on two full length profiles are shown for chromosome III (upper two diagrams) and chromosome V (lower two diagrams). (Full datasets are available at GEO, accession number GSE51977). ChIP-seq signals were smoothed at bandwidth 250 bp, decile normalized and background subtracted (minus untagged). White circle: Centromere. Black: Sgs1. Smc6 signals were plotted in green on the negative scale to facilitate comparison. The apparent Sgs1/Smc6 symmetry in the example region is corroborated genome-wide by the high Pearson correlation (pcorr = 0.8) over more than 6000 peaks per profile. (B) Vegetative synthetic interactions of the *mms21-11* mutant with resolvase and anti-recombinogenic helicase mutants assayed by colony size after spore germination. The relevant genotypes are indicated by color code (to the left of the spore colonies), red squares over colonies indicates the presence of double or triple mutations. For the *mus81Δ yen1Δ mms21-11* panel, yellow dashed squares are *mms21-11 yen1Δ*, the blue dashed square *mms21-11 mus81Δ* and the green dashed square *mus81Δ yen1Δ*. (C) Summary of observed synthetic interactions (colony size) and comparison with published ones (generation time in .002% MMS), [65]. Black indicates absence of synthetic growth defect, consecutive brighter shades of red indicate stronger synthetic effects (colony size). (n/a: not applicable, n/d: not determined).
(TIF)

Figure S8 Smc5/6-Mms21 is not essential for Sgs1 recruitment to meiotic DSB sites (related to Figure 6C, D). (A) Sgs1 does not require Mms21 SUMOylation activity for recruitment to DSB hotspots. qChIP of Sgs1-myc18 at various time points in meiosis (30°C) at three different DSB sites (DSB2: 130,7 kb [Chr I], DSB3: 190,5 kb [Chr I], DSB4: 96,1 kb [Chr IV]) shown for *MMS21* (blue bars) and *mms21-11* (red bars). Different shades represent the different hotspots. The Sgs1 signals in *mms21-11* follow those of wild type with a 1 hour delay. The right panel documents meiotic progression by spindle morphology of the same culture, revealing a 1 hour delay in the mutant culture. This delay is most likely due to synthety with the C-terminal myc-tag of Sgs1 because untagged *mms21-11* cells do not show a meiotic delay. (B) Sgs1 does not require Smc6 for recruitment to DSB hotspots at 33°C. qChIP for Sgs1-myc18 at various time points during meiosis (33°C) at two different DSB sites (DSB1: 211 kb [Chr III], DSB4: 96,1 kb [Chr IV]) shown for *SMC6* (blue bars) and *smc6-56* (red bars) and at Core1: 219,1 kb [Chr III] (*SMC6* (green bars) and *smc6-56* (brown bars)). The kinetics of Sgs1 signals in *smc6-56* appear to lag 1 hour behind. The right panel documents meiotic progression by spindle morphology of the same culture.
(TIF)

Figure S9 Topological model for early Smc5/6-Mms21 functions on dHJs (related to Figure 7). (A) Surveying for displaced ssDNA as a preloaded complex will inherently lock Smc5/6-Mms21 to the “outside” of recombinogenic lesions. In the case of a dHJ, such binding will label the parental side of the dHJ, opposed to the hetero-duplex DNA region. Stable binding to the HJ will subsequently disfavor dHJ extension as base pairing may be inhibited and dHJ extension would require overcoming a protruding parental DNA strand. (B) A single HJ is devoid of information regarding the inside or outside regions of a dHJ. At the least, inter-sister dHJs lack any hetero-duplex DNA which could help to distinguish the inside of a lesion from the outside. Thus, inappropriate association of Sgs1-Rmi1-Top3 may result in dHJ extension instead of dissolution. As preloaded Smc5/6-Mms21 binding to the ssDNA of a HJ will label the parental side of a dHJ, Smc5/6-Mms21 may provide a polarity to the HJ and guide the activity of Sgs1-Rmi1-Top3 towards dissolution of the dHJ.
(TIF)

File S1 A single pdf file containing all supporting figures, supporting tables and corresponding legends is provided for convenience.
(PDF)

Table S1 Yeast strains used in this study.
(DOCX)

Table S2 Antibodies used in this study.
(DOCX)
