# Supplementary figures and images for: Smc5/6-Mms21 Prevents and Eliminates Inappropriate Recombination Intermediates in Meiosis
Source: PLoS Genet. 2013 Dec 26;9(12):e1004067. doi: 10.1371/journal.pgen.1004067 (PMC3873250; doi:10.1371/journal.pgen.1004067)

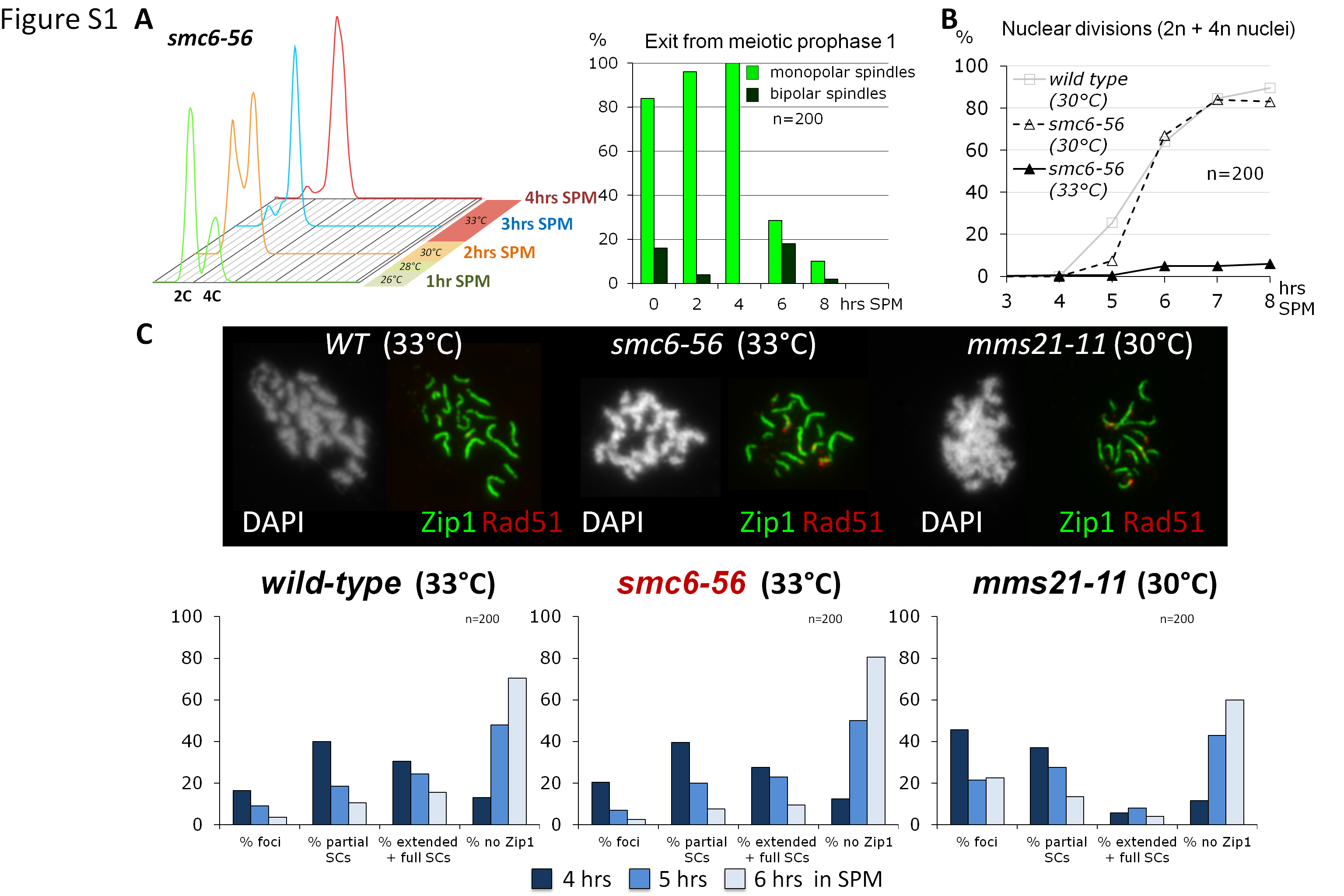

Supplement: Figure S1 — Meiosis specific depletion of Smc6 leaves meiotic progression and chromosome synapsis unaffected (Related to Figure 1). (A) A time series of FACS profiles for pre-meiotic DNA replication in the smc6-56 mutant from indicated time points post induction of meiosis and under our regiment for gradual inactivation of Smc5/6 at late S-phase. Subsequent meiotic progression is shown for this time course experiment on meiotic spindles (n = 200 for each time point), as exit from meiotic prophase 1. (B) The semi-permissive temperature of 30°C for the smc6-56 allele permits meiotic DNA segregation while the restrictive temperature of 33°C does not. Meiotic nuclei of ethanol fixed and DAPI stained cells were counted at the indicated time points and temperatures (n = 200). The sum of bi- and tetra-nucleated cells (2n+4n) is plotted. (C) Upper panels: Three spread nuclei representing examples of pachytene cells showing no aberrant features from wild type, smc6-56 and mms21-11 mutants as indicated. White: DAPI stained chromosomes, Green: Zip1 protein demonstrating full synapsis, Red: Few, normal residual Rad51 foci. Lower panels: Quantification of synapsis in wild type, smc6-56 and mms21-11 diploids from 4, 5 and 6 hour time points of sporulation (different shades of blue) at the temperatures indicated. All mutants form normal SC, although mms21-11 showed a reduced yield of extended and full SCs at lower temperature. (TIF) [file pgen.1004067.s001.tif]

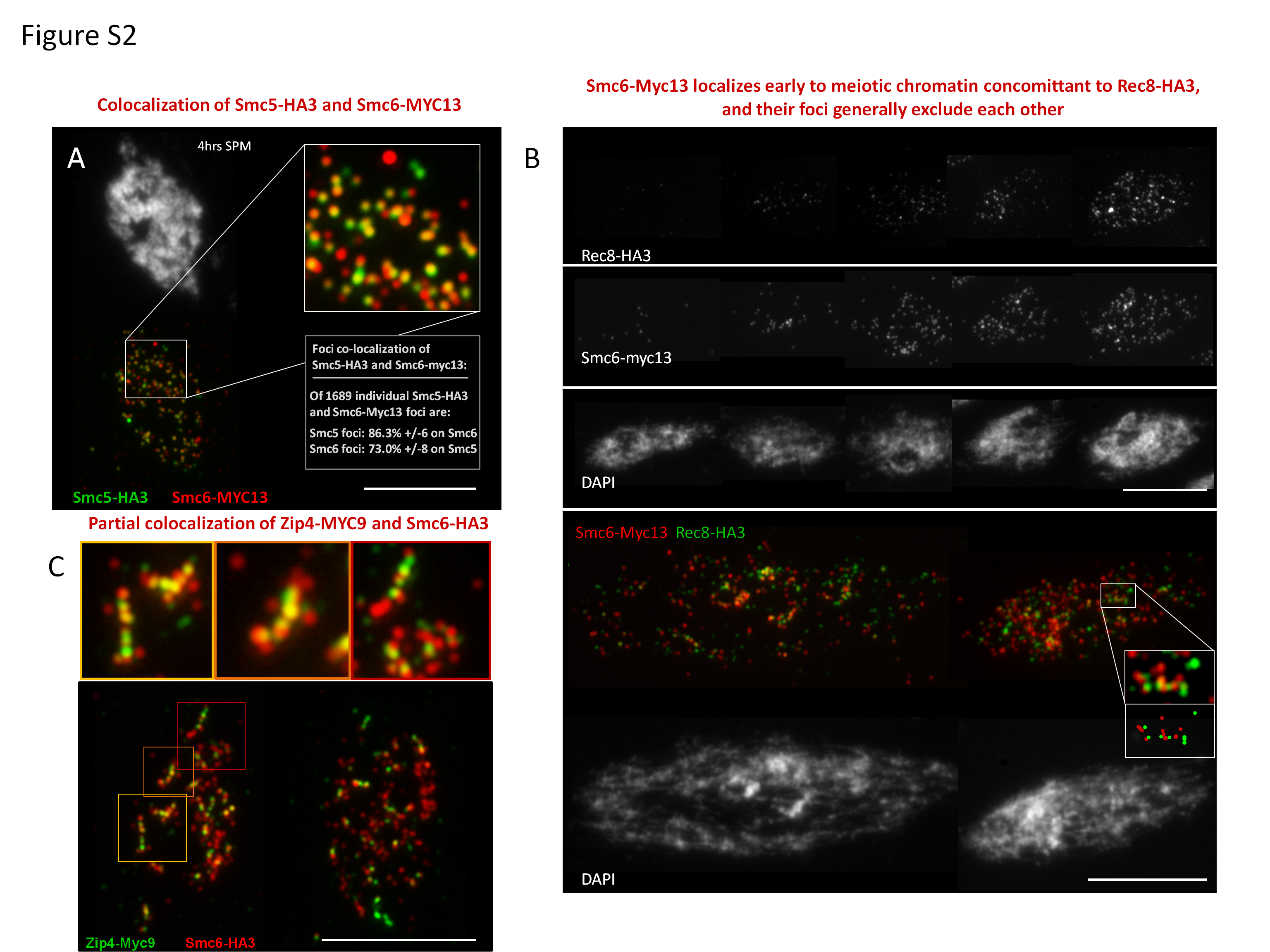

Supplement: Figure S2 — Binding pattern of Smc6 on meiotic chromatin by cytology (related to Figure 2). (A) Smc5-HA3 and Smc6-myc13 foci colocalize: Red: Smc6-myc13, Green: Smc5-HA3, white bar: 10 µm. White rectangle indicates position of magnified sub region. Co-localization for 1689 foci of 8 prophase 1 nuclei was determined. (B) Smc6-myc13 localizes early to meiotic chromatin, concomitant with the appearance of Rec8-HA3 foci. 5 nuclei (from 2 h in SPM) represent consecutive stages from left to right, based on the abundance of Rec8-HA foci. Panels from top: Rec8-HA3, Smc6-myc13, chromatin stained with DAPI. White bar: 10 µm. Colored Panel: Overlay of Rec8 and Smc6 signals reveal very little overlap in early nuclei. Red: Smc6-myc13, Green: Rec8-HA3, White: DAPI. White rectangle indicates position of magnified sub region. Upon close inspection, none of the early Rec8 and Smc6 foci in the region show significant overlap. (C) Partial co-localization between Zip4-myc9 and Smc6-HA3. Nuclei from 4 hours in SPM show limited on top but frequent side by side localization, similar to the situation between Rad51 and Smc6. Red: Smc6-HA3, green: Zip4-myc9, white bar: 10 µm. White rectangle indicates position of magnified sub region. (TIF) [file pgen.1004067.s002.tif]

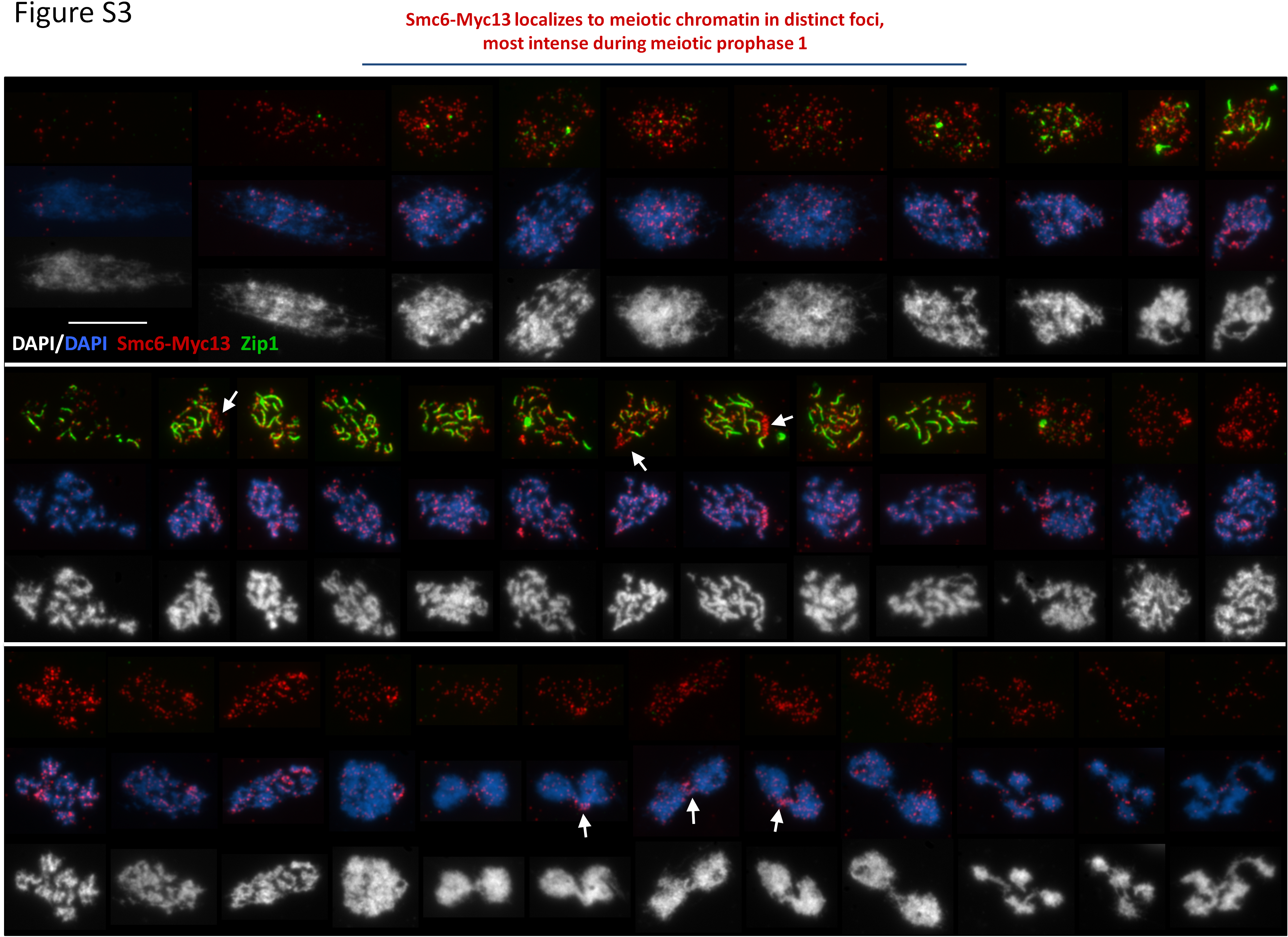

Supplement: Figure S3 — Chromatin binding of Smc6 throughout meiosis by cytology (related to Figure 2). 35 spread nuclei of various meiotic stages sorted in a consecutive order from left to right and from top to bottom by combining information from Zip1 and DAPI morphologies. Each nucleus is represented by three micrographs, a green and red merge (between Zip1 and Smc6, respectively), a merge between blue and red (DAPI and Smc6) and a white micrograph (DAPI staining of DNA). The upper three rows depict stages from the earliest appearance of Smc6 representing presumably (early) replicating nuclei, to leptotene and zygotene. The three lanes in the middle represent zygotene, pachytene and post pachytene stages (presumably corresponding to diplotene). Three white arrowheads point at dense clusters of Smc6 foci accumulating at the unsynapsed regions of chromosome XII, representing the rDNA. The bottom three lanes correspond to late stages: Prometaphase/metaphase, as well as several anaphase I and anaphase II examples. Three white arrowheads point at dense clusters of Smc6 foci accumulating at the residual connecting chromatin between the almost pinched off nuclei. White bar: 10 µm. (TIF) [file pgen.1004067.s003.tif]

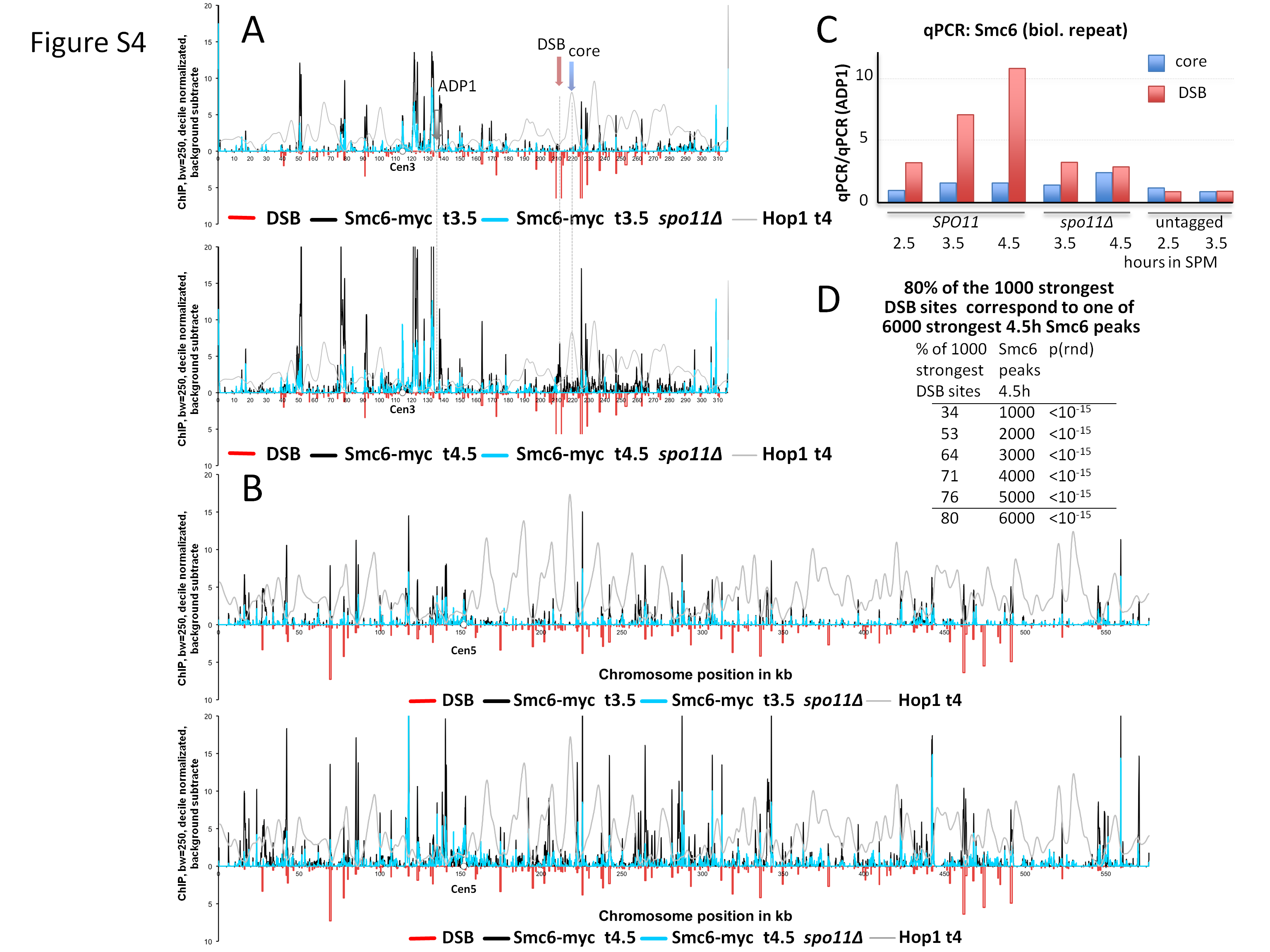

Supplement: Figure S4 — Smc6 is recruited to meiotic DSB hotspots where it enriches to upon DSB formation (related to Figure 2D,E). ChIP-seq profiles for complete chromosomes. (A, B) The upper two panels show chromosome III, the lower two chromosome V. Of the two panels depicting the same chromosome the upper one corresponds to time point 3.5 hours in SPM, the lower one to 4.5 hours in SPM. The color coding is: Black: Smc6-myc13, blue: Smc6-myc13, spo11Δ, red: DSBs shown on the negative axis for better comparison. A profile of chromosome axis protein Hop1 (taken from [51]) was added in grey to mark axis sites. Appropriately colored arrows point to sites where the primers for cold region, hotspot and core used for qPCR are located. Here ChIP-seq profiles were smoothed (bw = 250 bp), decile normalized and background subtracted (except for Hop1). (C) qPCR of a biological repeat at three positions on chromosome III from the same experiment, a DSB site (ca. at 211k, YCR047C), a core site (ca. at 219k) and a cold spot (ca. at 136, ADP1). To correct for possible differences in the efficiencies of the IPs across the different strains, the enrichment of core and DSB qPCR signals relative to the ADP1 signal is plotted for the indicated genotypes and time points, Core/ADP1 shown in blue, DSB/ADP1 in red. (D) Overlap of Smc6 (4.5 h) peaks with the 1000 strongest DSB hotspots [53]. A peak is considered overlapping if it lies within the borders of the hotspot. Left column: % of DSB hotpots that are hit by Smc6 peaks. Middle column: The number of Smc6 peaks required to hit the corresponding percentage of DSB hotspots. Right column: The significance (binomial random model), which was in all cases below the smallest value displayed by R (10−15). Because the strongest hotspots do not always correspond to the strongest Smc6 peaks, naturally, there is only 34% overlap between the 1000 strongest Smc6 (t4.5) and DSB sites. However, nearly all of the 1000 strongest DSB hotspots contain an Smc6 peak if smaller Sm [file pgen.1004067.s004.tif]

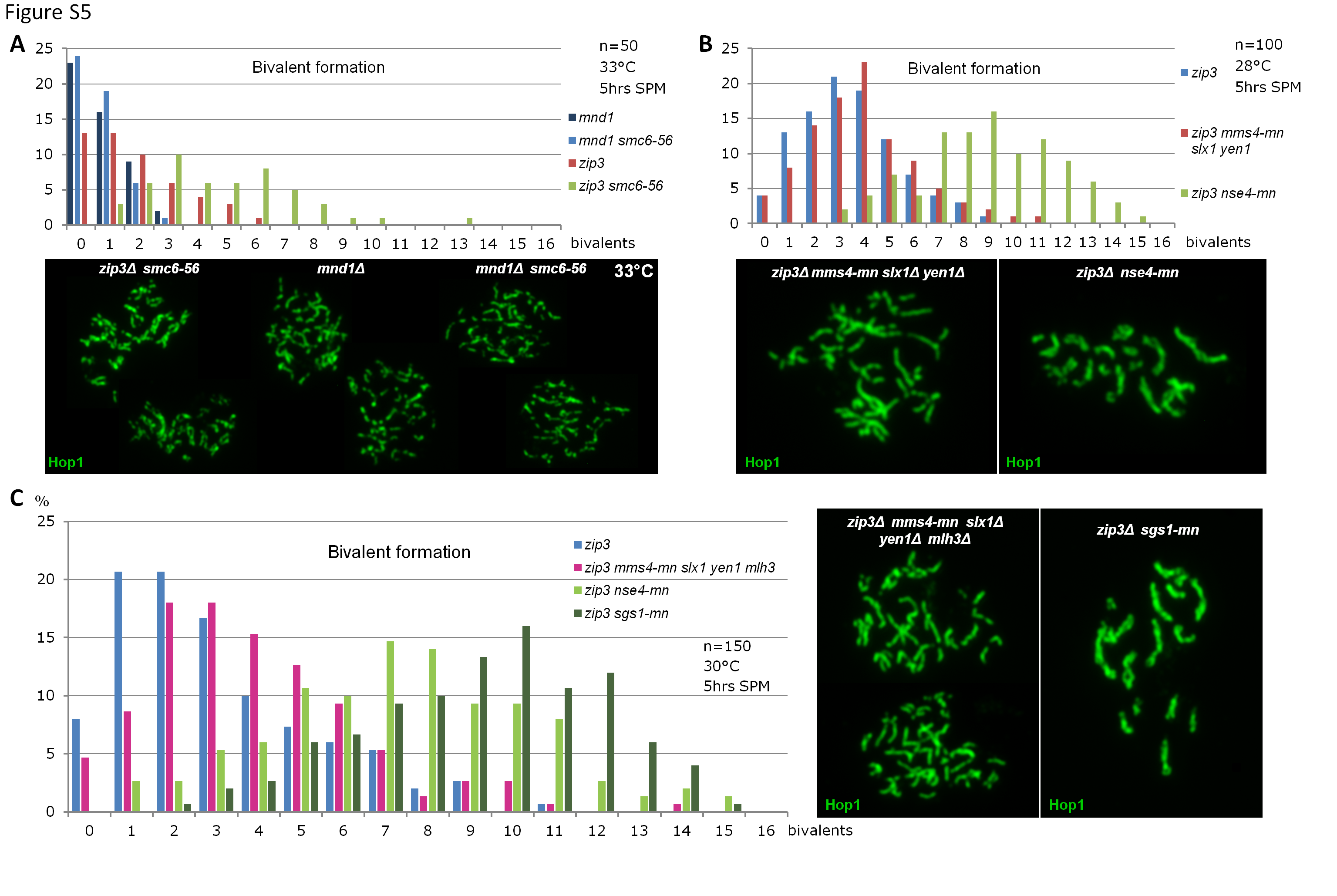

Supplement: Figure S5 — Smc5/6 mutants promote the formation of inappropriate, stable recombination intermediates (related to Figure 4). (A) smc6-56 does not promote axis pairing in the mnd1Δ mutant which is defective for the DSB strand invasion step in meiotic homologous recombination. Upper panel: Quantification of bivalent formation on spread nuclei of 5 hrs SPM stained against Hop1 (n = 50 for each mutant). Number of incidences is plotted for each category of “0” to “16” bivalents. Dark blue: mnd1Δ, blue: mnd1Δ smc6-56, red: zip3Δ, green: zip3Δ smc6-56 (all at 33°C). Lower panel: Representative nuclei of the indicated mutants at 5 hrs SPM after chromosome spreading and staining against Hop1 (green). (B) Inactivation of the rogue JM resolvases cannot promote stabilization of axial associations in the zip3Δ mutant while inactivation of Smc5/6-Mms21 does, indicative of inappropriate JM formation. As in (A): Upper panel: Quantification of bivalent formation on spread nuclei of 5 hrs SPM stained against Hop1 (n = 100 for each mutant). Blue: zip3Δ, red: zip3Δ mms4-mn slx1Δ yen1Δ, green: zip3Δ nse4-mn (at 28°C). Lower panel: Representative nuclei of the indicated mutants at 5 hrs SPM. Hop1 in green. (C) Removal of all characterized meiotic resolvase activity cannot promote chromosome pairing in zip3Δ such as observed in zip3Δ sgs1-mn and zip3Δ nse4-mn. Left panel: Quantification of bivalent formation on spread nuclei of 5 hrs SPM stained against Hop1, plotted as percentage of incidents (n = 150 for each mutant). Blue: zip3Δ, pink: zip3Δ mms4-mn slx1Δ yen1Δ mlh3Δ, light green: zip3Δ nse4-mn, dark green: zip3Δ sgs1-mn (at 30°C). Right panel: Representative nuclei of the indicated mutants at 5 hrs SPM. Hop1 in green. (TIF) [file pgen.1004067.s005.tif]

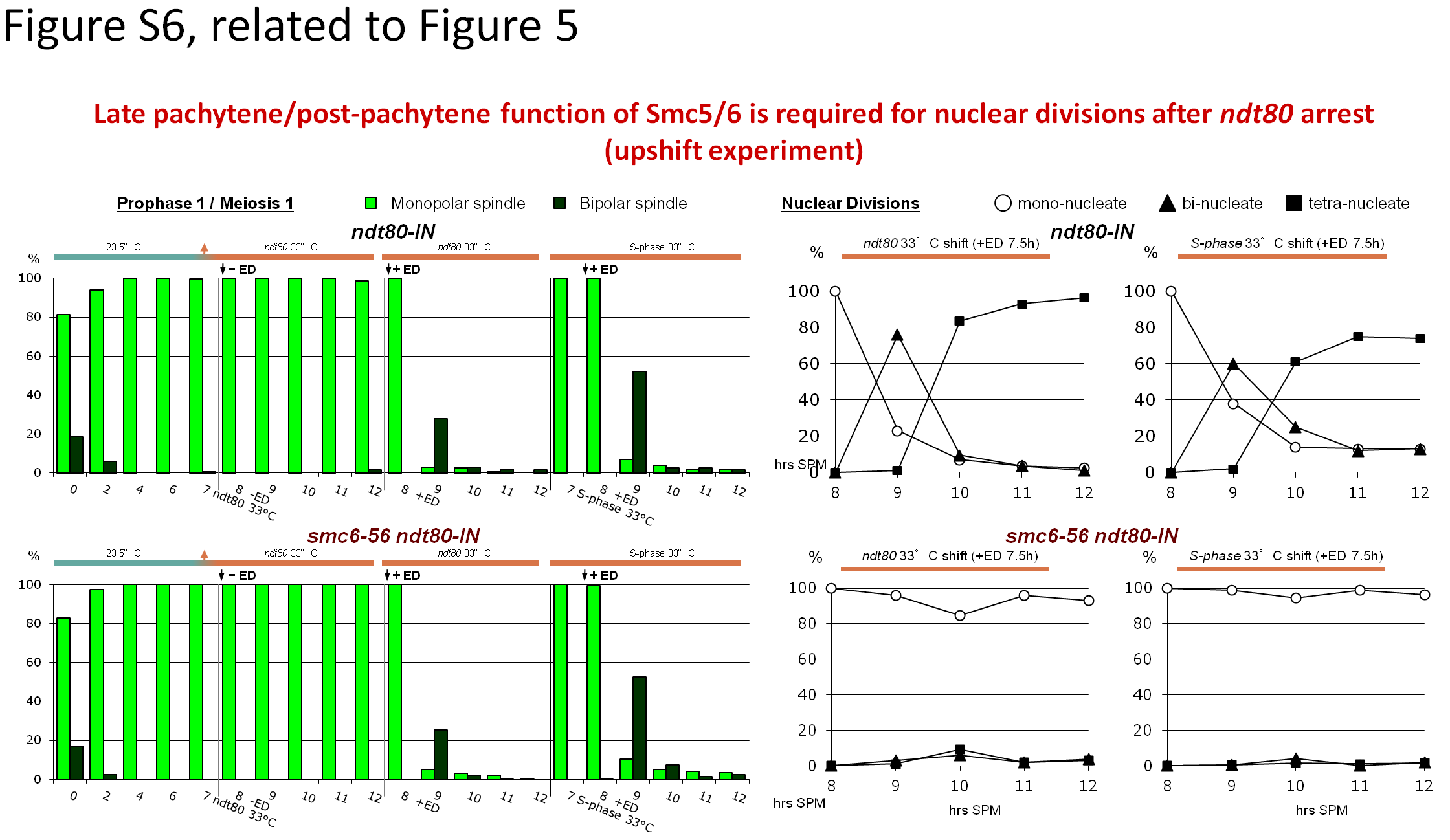

Supplement: Figure S6 — Inactivation of Smc5/6-Mms21 at pachytene exit prevents nuclear divisions (related to Figure 5). A shift to restrictive temperature during ndt80 release abolishes nuclear divisions in smc6-56, indicating that early function of Smc5/6-Mms21 is not sufficient to relieve the block of division, but that late functions are required. The experiment is related to Figure 5 (H,I), except that in this experiment the temperature regime was inverted, starting with meiosis at permissive temperature until the release from the ndt80-IN arrest. Left panels show meiotic progression in response to estradiol addition, as monitored by spindle staining. The permissive (23.5°C) and restrictive (33°C) temperatures are indicated by turquoise and orange colors above the bar diagram. ED indicates the timing of addition of estradiol to induced Ndt80 expression and exit from pachytene. In contrast, a shift to permissive temperature during Ndt80 release restores nuclear divisions in smc6-56 (Figure 5I). Upper panels: Wild type (ndt80-IN), lower panels: smc6-56 (ndt80-IN). Light green bars: Monopolar spindles, dark green bars bipolar spindles. From left to right: 1st panel: Time course starting under permissive conditions in the ndt80-IN strain background in the absence of inducer (estradiol). 2nd panel: shift to restrictive temperature (33°C), without release (− estradiol). 3rd panel: release (+ estradiol) into restrictive temperature (causes short and synchronous burst of bipolar spindles). 4rd panel: release (+ estradiol) of cells that started meiosis under restrictive conditions into restrictive temperature (positive control for blocked divisions, causes short and synchronous burst of bipolar spindles). The four diagrams on the right show nuclear divisions as a function of time post Ndt80 expression: % 1n (circle), 2n (triangle) and 4n (squares, n = number of nuclei) plotted against hours in SPM. Upper two panels show normal divisions in SMC6, independent of treatments. Lower panels: Nuclear [file pgen.1004067.s006.tif]

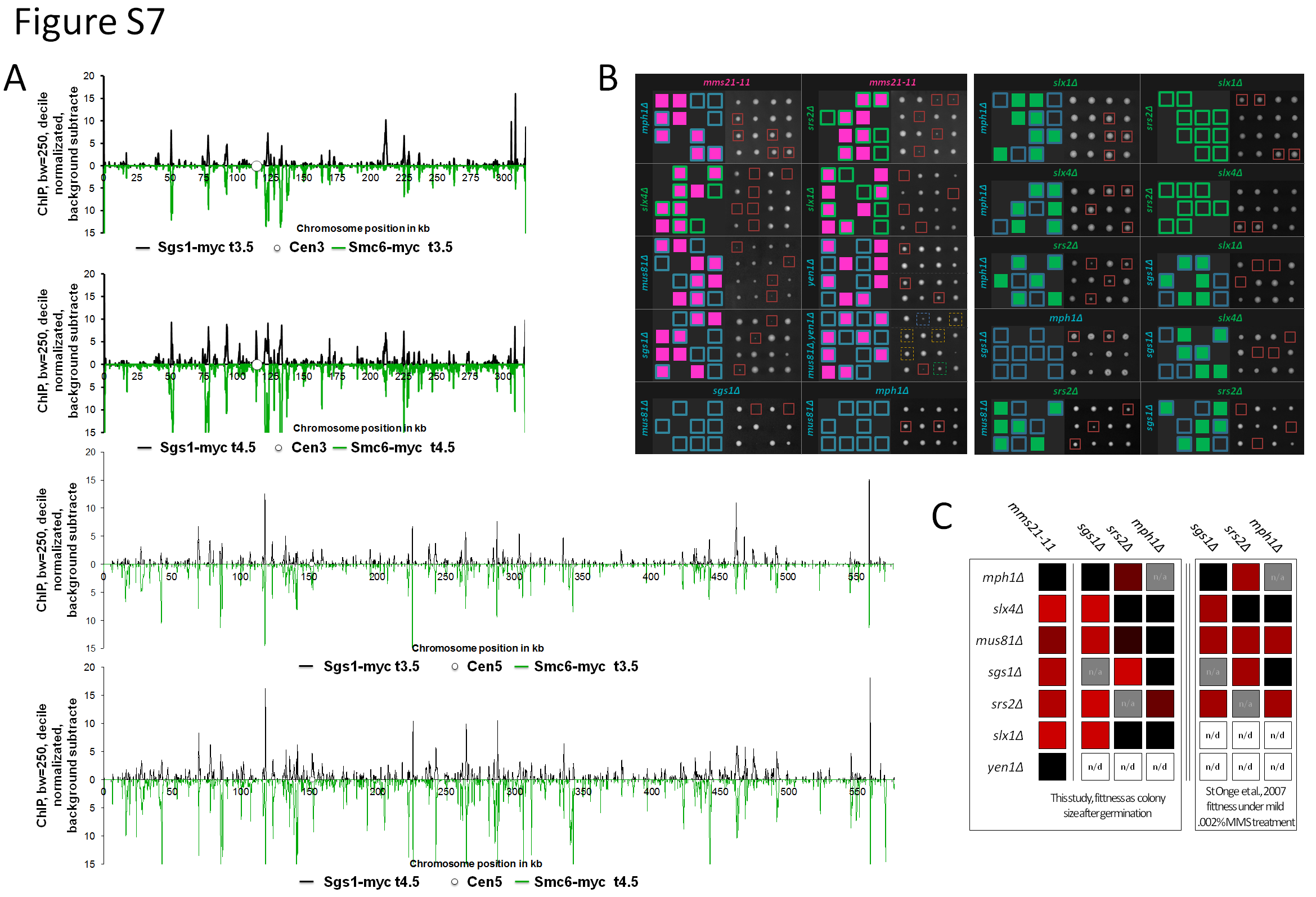

Supplement: Figure S7 — Analogies between Smc5/6-Mms21 and Sgs1 (related to Figure 6). (A) Chromosomal interaction sites and intensities are highly similar between Sgs1 and Smc6. DNA-interaction sites for Sgs1-myc18 (t = 4.5 hours in SPM) and Smc6-myc13 (t = 3.5 hours in SPM) on two full length profiles are shown for chromosome III (upper two diagrams) and chromosome V (lower two diagrams). (Full datasets are available at GEO, accession number GSE51977). ChIP-seq signals were smoothed at bandwidth 250 bp, decile normalized and background subtracted (minus untagged). White circle: Centromere. Black: Sgs1. Smc6 signals were plotted in green on the negative scale to facilitate comparison. The apparent Sgs1/Smc6 symmetry in the example region is corroborated genome-wide by the high Pearson correlation (pcorr = 0.8) over more than 6000 peaks per profile. (B) Vegetative synthetic interactions of the mms21-11 mutant with resolvase and anti-recombinogenic helicase mutants assayed by colony size after spore germination. The relevant genotypes are indicated by color code (to the left of the spore colonies), red squares over colonies indicates the presence of double or triple mutations. For the mus81Δ yen1Δ mms21-11 panel, yellow dashed squares are mms21-11 yen1Δ, the blue dashed square mms21-11 mus81Δ and the green dashed square mus81Δ yen1Δ. (C) Summary of observed synthetic interactions (colony size) and comparison with published ones (generation time in .002% MMS), [65]. Black indicates absence of synthetic growth defect, consecutive brighter shades of red indicate stronger synthetic effects (colony size). (n/a: not applicable, n/d: not determined). (TIF) [file pgen.1004067.s007.tif]

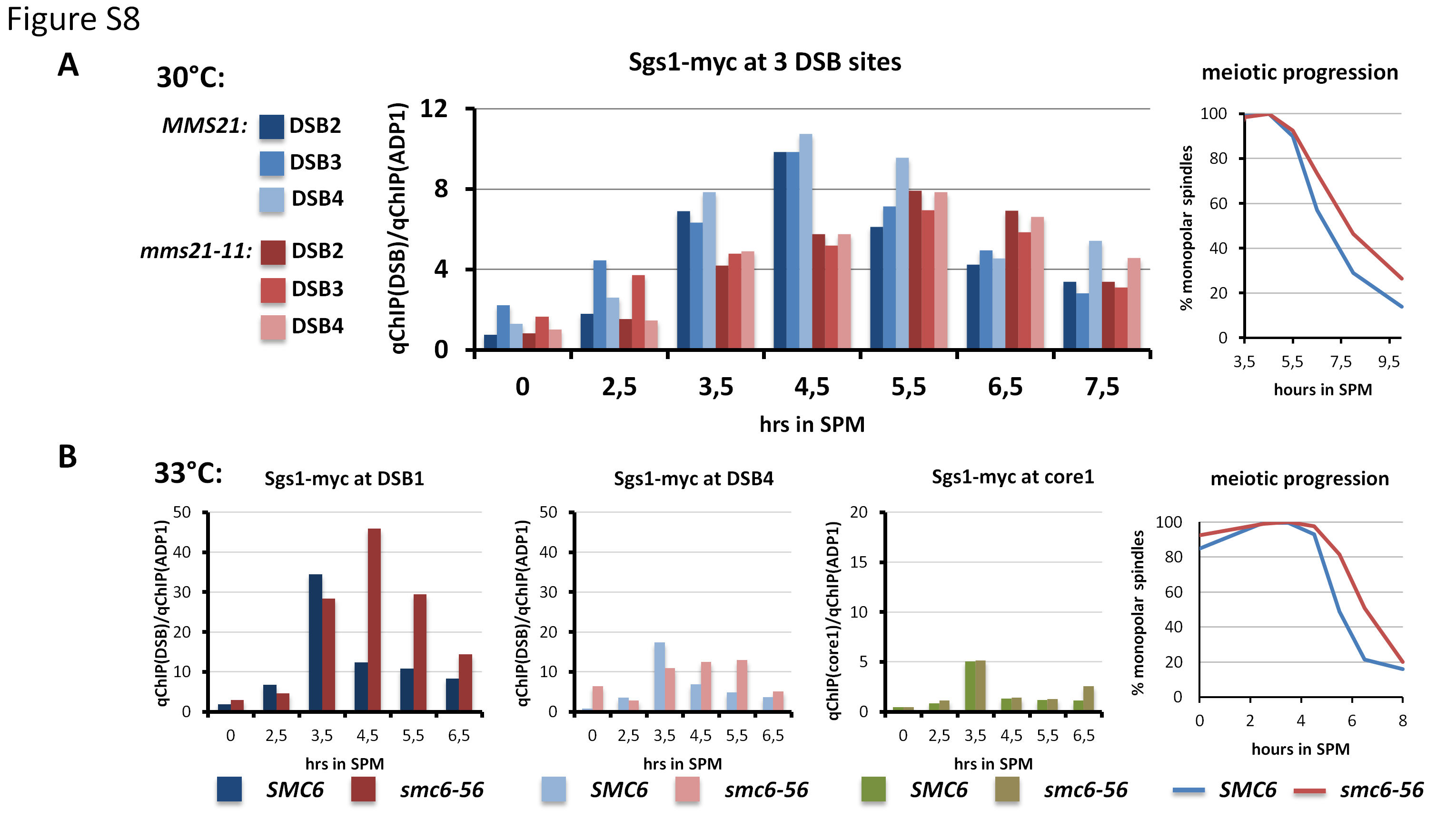

Supplement: Figure S8 — Smc5/6-Mms21 is not essential for Sgs1 recruitment to meiotic DSB sites (related to Figure 6C, D). (A) Sgs1 does not require Mms21 SUMOylation activity for recruitment to DSB hotspots. qChIP of Sgs1-myc18 at various time points in meiosis (30°C) at three different DSB sites (DSB2: 130,7 kb [Chr I], DSB3: 190,5 kb [Chr I], DSB4: 96,1 kb [Chr IV]) shown for MMS21 (blue bars) and mms21-11 (red bars). Different shades represent the different hotspots. The Sgs1 signals in mms21-11 follow those of wild type with a 1 hour delay. The right panel documents meiotic progression by spindle morphology of the same culture, revealing a 1 hour delay in the mutant culture. This delay is most likely due to synthety with the C-terminal myc-tag of Sgs1 because untagged mms21-11 cells do not show a meiotic delay. (B) Sgs1 does not require Smc6 for recruitment to DSB hotspots at 33°C. qChIP for Sgs1-myc18 at various time points during meiosis (33°C) at two different DSB sites (DSB1: 211 kb [Chr III], DSB4: 96,1 kb [Chr IV]) shown for SMC6 (blue bars) and smc6-56 (red bars) and at Core1: 219,1 kb [Chr III] (SMC6 (green bars) and smc6-56 (brown bars)). The kinetics of Sgs1 signals in smc6-56 appear to lag 1 hour behind. The right panel documents meiotic progression by spindle morphology of the same culture. (TIF) [file pgen.1004067.s008.tif]

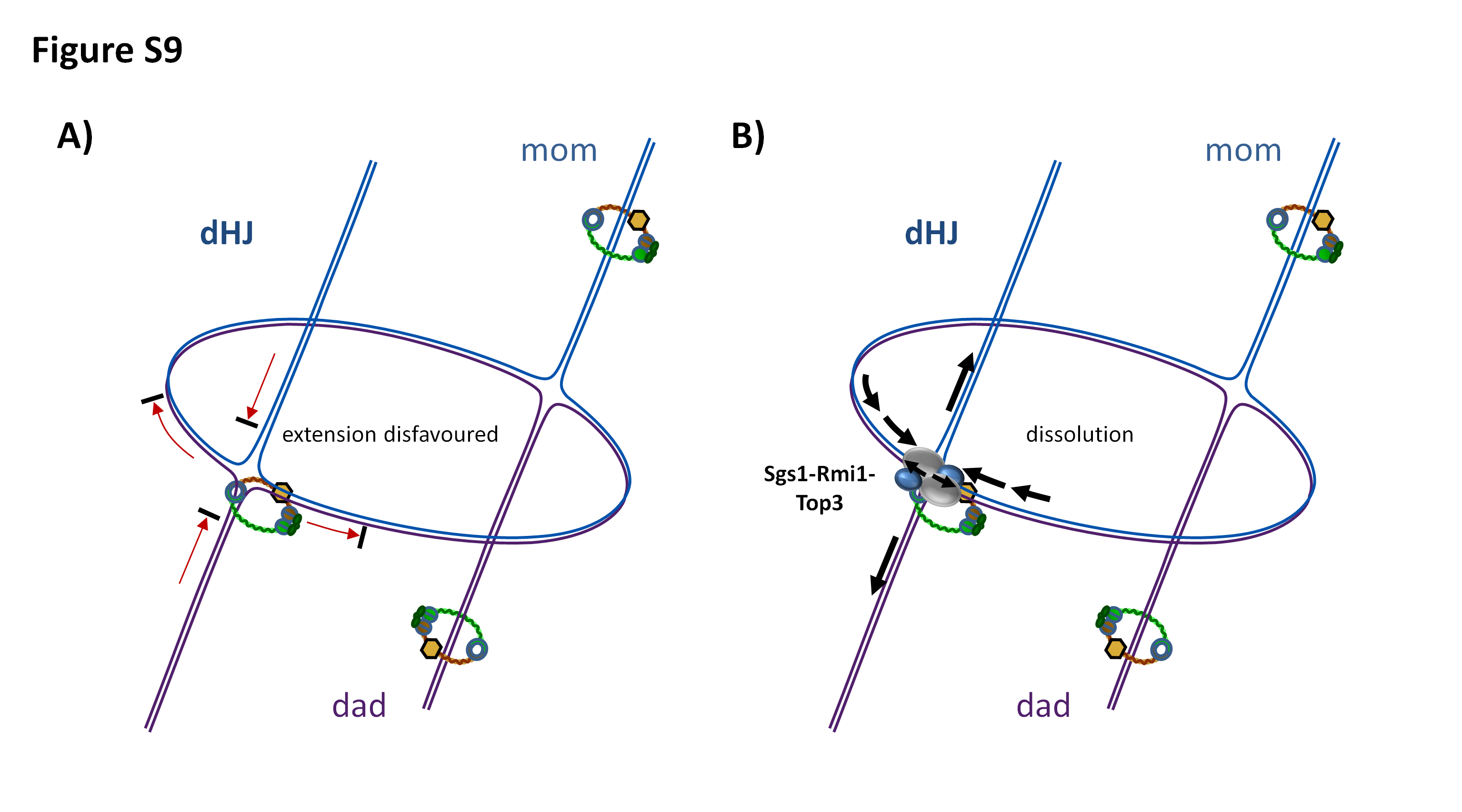

Supplement: Figure S9 — Topological model for early Smc5/6-Mms21 functions on dHJs (related to Figure 7). (A) Surveying for displaced ssDNA as a preloaded complex will inherently lock Smc5/6-Mms21 to the “outside” of recombinogenic lesions. In the case of a dHJ, such binding will label the parental side of the dHJ, opposed to the hetero-duplex DNA region. Stable binding to the HJ will subsequently disfavor dHJ extension as base pairing may be inhibited and dHJ extension would require overcoming a protruding parental DNA strand. (B) A single HJ is devoid of information regarding the inside or outside regions of a dHJ. At the least, inter-sister dHJs lack any hetero-duplex DNA which could help to distinguish the inside of a lesion from the outside. Thus, inappropriate association of Sgs1-Rmi1-Top3 may result in dHJ extension instead of dissolution. As preloaded Smc5/6-Mms21 binding to the ssDNA of a HJ will label the parental side of a dHJ, Smc5/6-Mms21 may provide a polarity to the HJ and guide the activity of Sgs1-Rmi1-Top3 towards dissolution of the dHJ. (TIF) [file pgen.1004067.s009.tif]
