# Supplementary material for: Smc5/6-Mms21 Prevents and Eliminates Inappropriate Recombination Intermediates in Meiosis
Source: PLoS Genet. 2013 Dec 26;9(12):e1004067. doi: 10.1371/journal.pgen.1004067 (PMC3873250; doi:10.1371/journal.pgen.1004067)
Supplement: File S1 — A single pdf file containing all supporting figures, supporting tables and corresponding legends is provided for convenience. (PDF) [file pgen.1004067.s010.pdf]

Figure S1

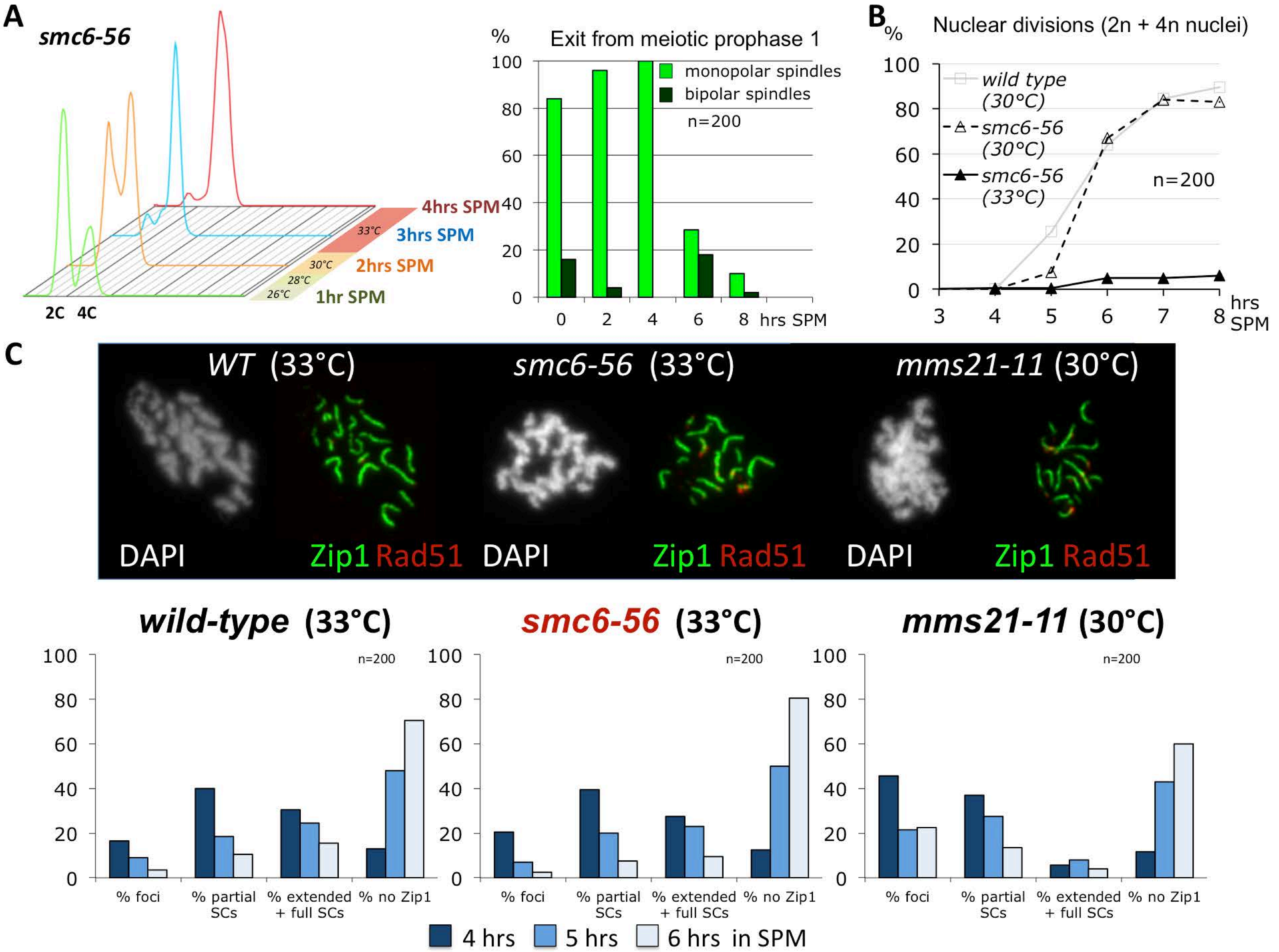

A
smc6-56
2C 4C
26°C
28°C
30°C
33°C
1hr SPM
2hrs SPM
3hrs SPM
4hrs SPM
Exit from meiotic prophase 1
%
monopolar spindles
bipolar spindles
n=200
0 2 4 6 8 hrs SPM
B
Nuclear divisions (2n + 4n nuclei)
wild type (30°C)
smc6-56 (30°C)
smc6-56 (33°C)
n=200
hrs SPM
C
WT (33°C)
smc6-56 (33°C)
mms21-11 (30°C)
DAPI
Zip1 Rad51
wild-type (33°C)
smc6-56 (33°C)
mms21-11 (30°C)
n=200
% foci
% partial SCs
% extended + full SCs
% no Zip1
4 hrs
5 hrs
6 hrs in SPM

Figure S2

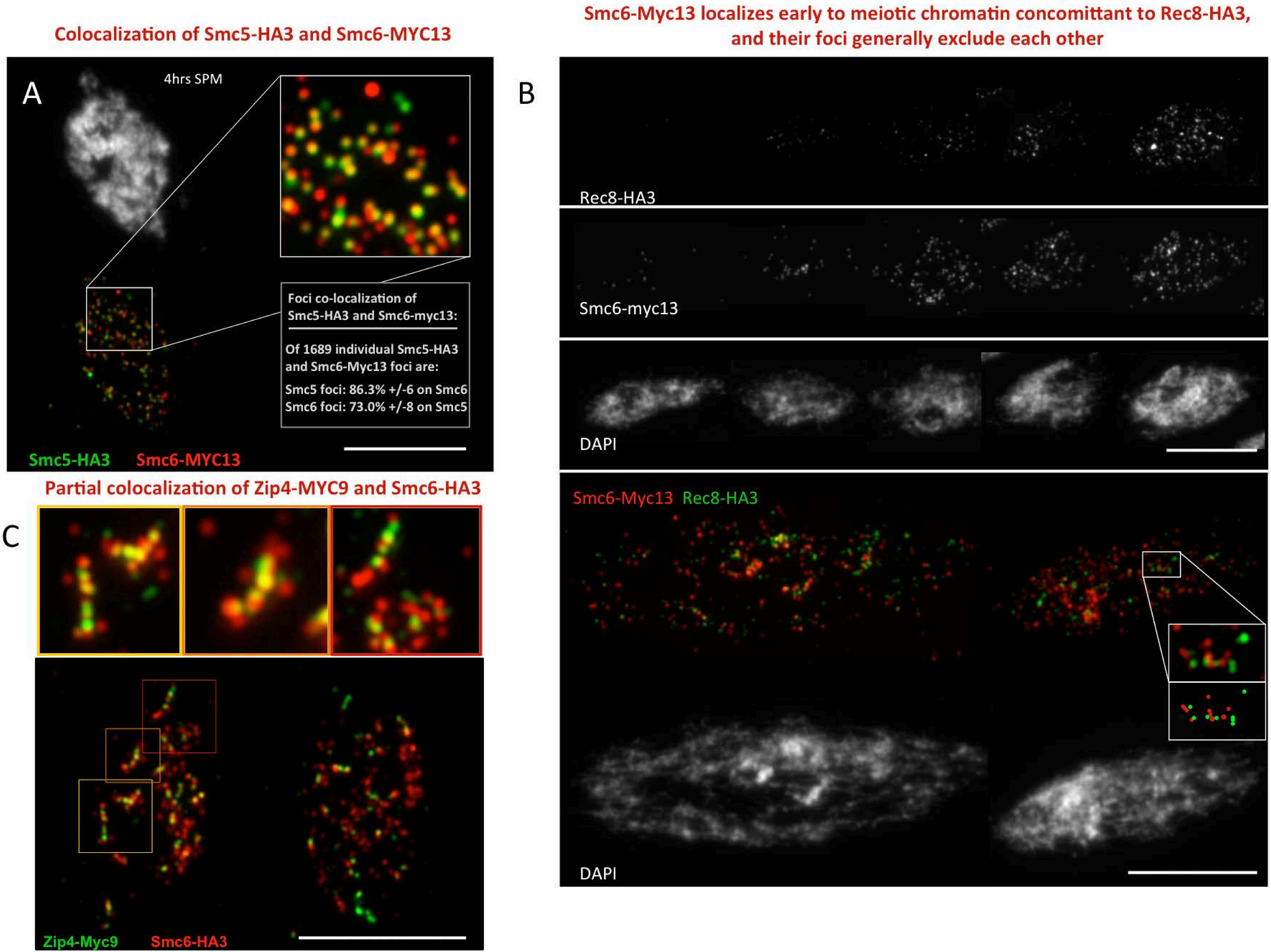
Colocalization of Smc5-HA3 and Smc6-MYC13
Smc6-Myc13 localizes early to meiotic chromatin concomittant to Rec8-HA3, and their foci generally exclude each other
A
4hrs SPM
Foci co-localization of Smc5-HA3 and Smc6-myc13:
Of 1689 individual Smc5-HA3 and Smc6-Myc13 foci are:
Smc5 foci: 86.3% +/-6 on Smc6
Smc6 foci: 73.0% +/-8 on Smc5
Smc5-HA3
Smc6-MYC13
B
Rec8-HA3
Smc6-myc13
DAPI
Smc6-Myc13
Rec8-HA3
DAPI
Partial colocalization of Zip4-MYC9 and Smc6-HA3
C
Zip4-Myc9
Smc6-HA3

Figure S3

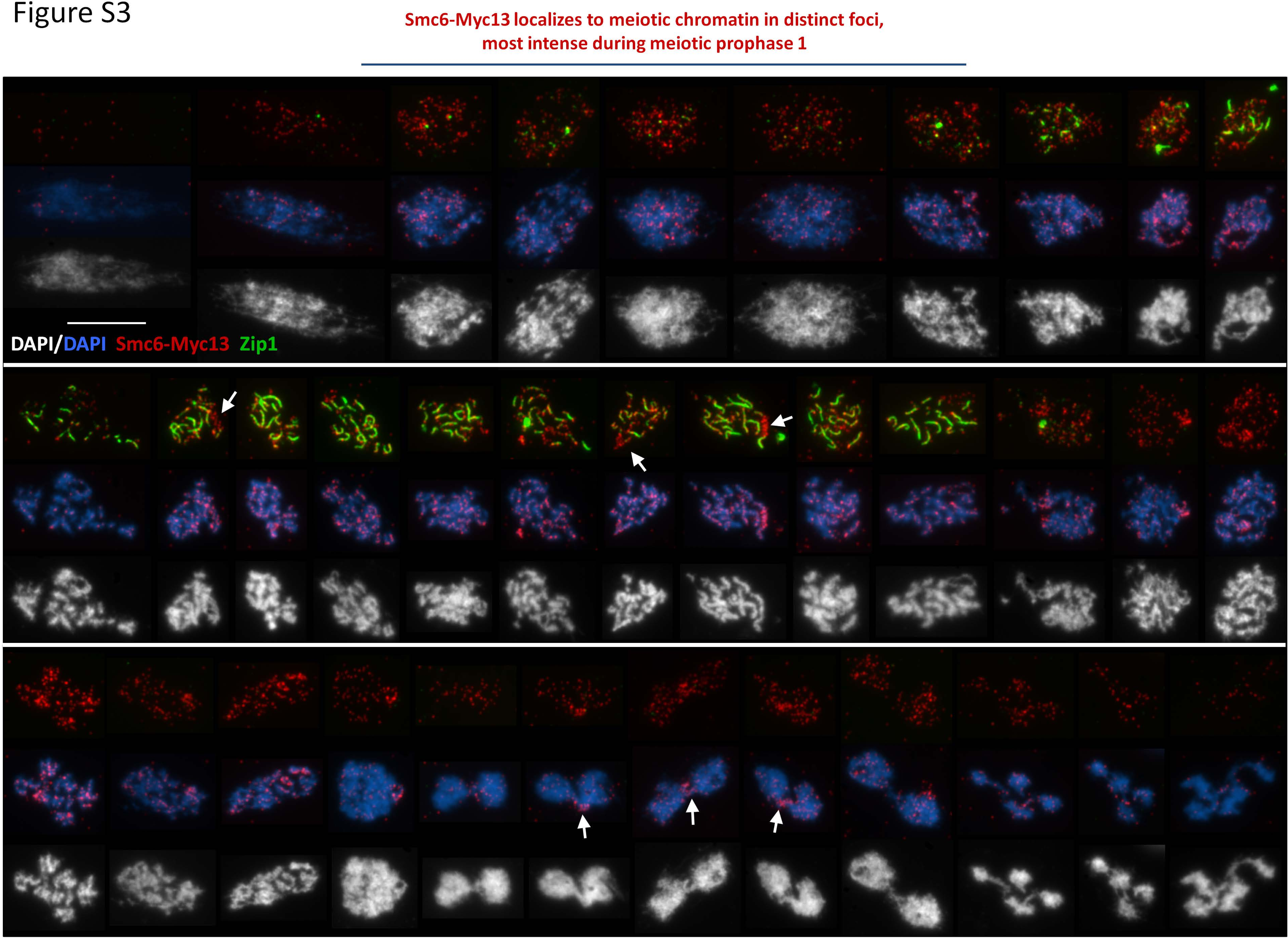
Smc6-Myc13 localizes to meiotic chromatin in distinct foci,
most intense during meiotic prophase 1
DAPI/DAPI Smc6-Myc13 Zip1

Figure S4

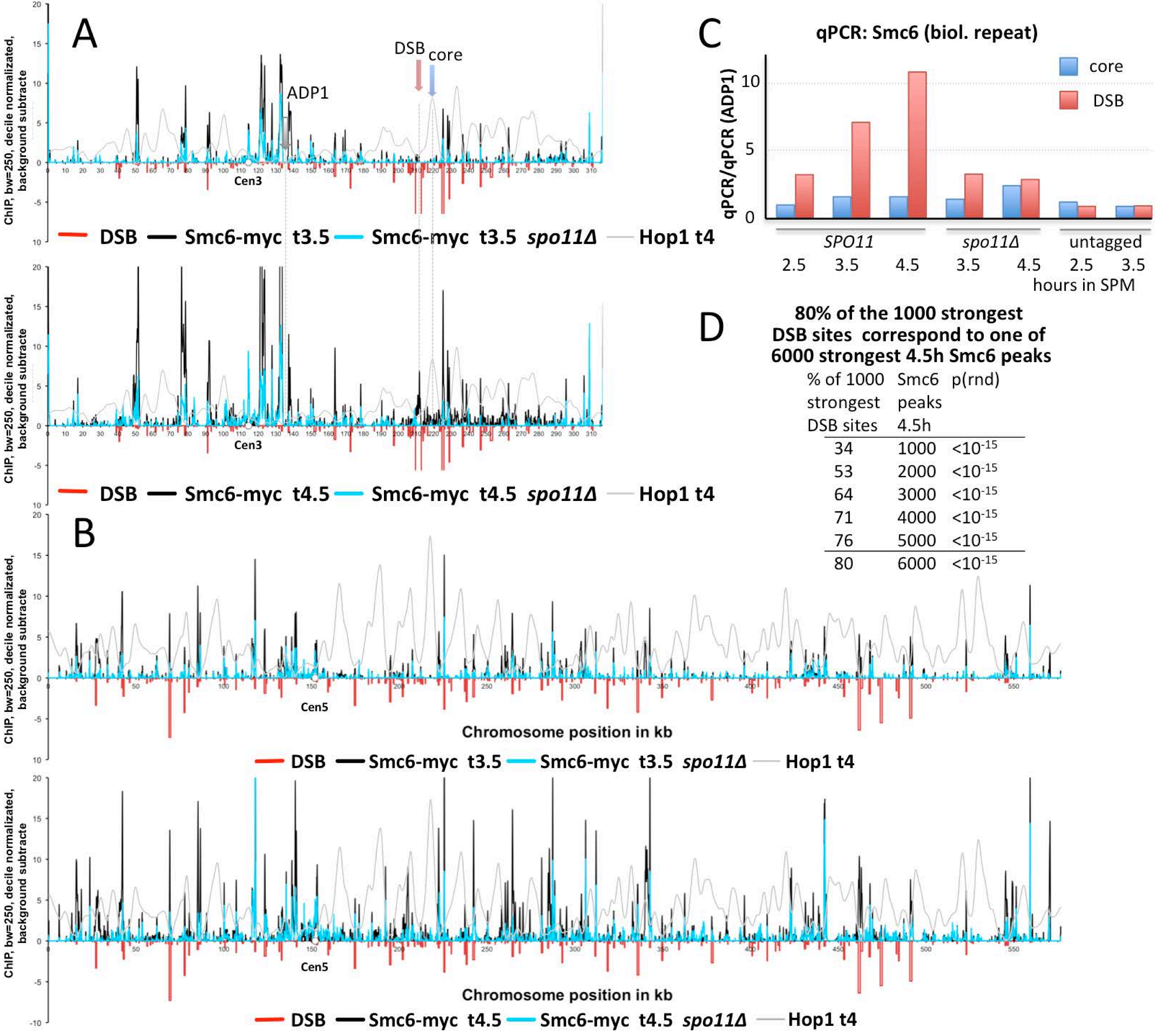

% of 1000 strongest DSB sites	Smc6 peaks 4.5h	p(rnd)
34	1000	$<10^{-15}$
53	2000	$<10^{-15}$
64	3000	$<10^{-15}$
71	4000	$<10^{-15}$
76	5000	$<10^{-15}$
80	6000	$<10^{-15}$

Figure S5

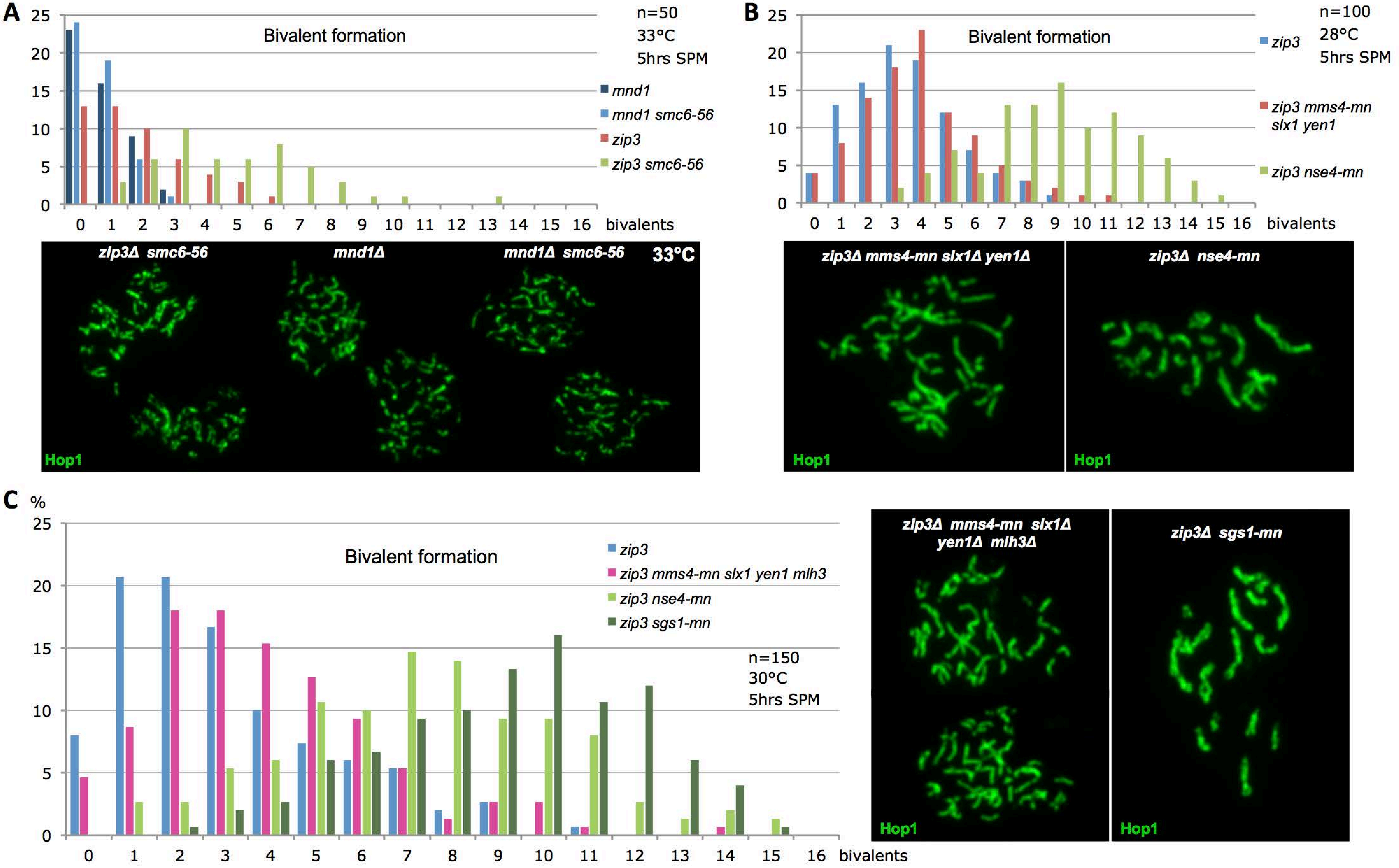
A
Bivalent formation
n=50
33°C
5hrs SPM
mnd1
mnd1 smc6-56
zip3
zip3 smc6-56
0 5 10 15 20 25
0 1 2 3 4 5 6 7 8 9 10 11 12 13 14 15 16 bivalents
zip3Δ smc6-56
mnd1Δ
mnd1Δ smc6-56
33°C
Hop1
B
Bivalent formation
n=100
28°C
5hrs SPM
zip3
zip3 mms4-mn slx1 yen1
zip3 nse4-mn
0 5 10 15 20 25
0 1 2 3 4 5 6 7 8 9 10 11 12 13 14 15 16 bivalents
zip3Δ mms4-mn slx1Δ yen1Δ
zip3Δ nse4-mn
Hop1
Hop1
C
%
Bivalent formation
zip3
zip3 mms4-mn slx1 yen1 mlh3
zip3 nse4-mn
zip3 sgs1-mn
n=150
30°C
5hrs SPM
0 5 10 15 20 25
0 1 2 3 4 5 6 7 8 9 10 11 12 13 14 15 16 bivalents
zip3Δ mms4-mn slx1Δ yen1Δ mlh3Δ
zip3Δ sgs1-mn
Hop1
Hop1

Figure S6, related to Figure 5

Late pachytene/post-pachytene function of Smc5/6 is required for nuclear divisions after *ndt80* arrest (upshift experiment)

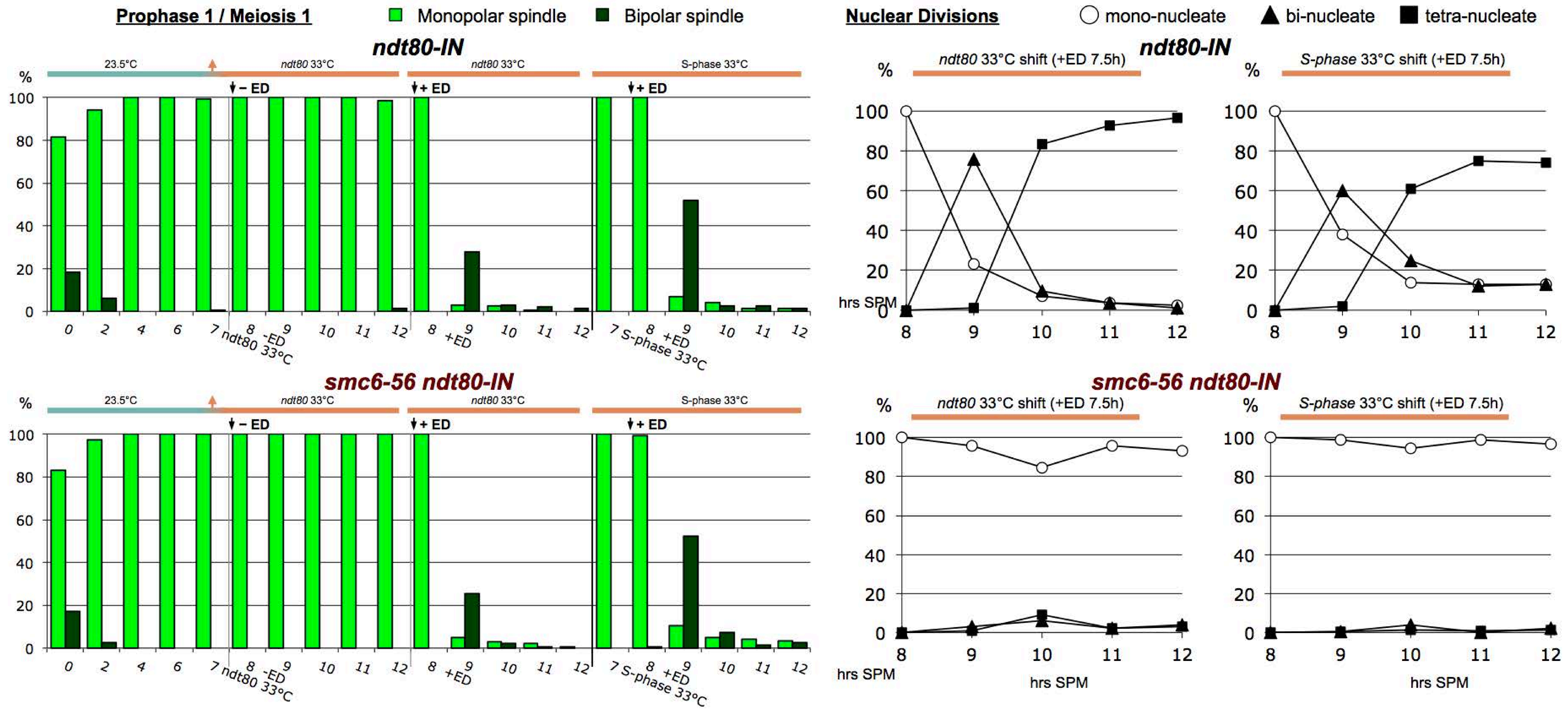

Figure S7

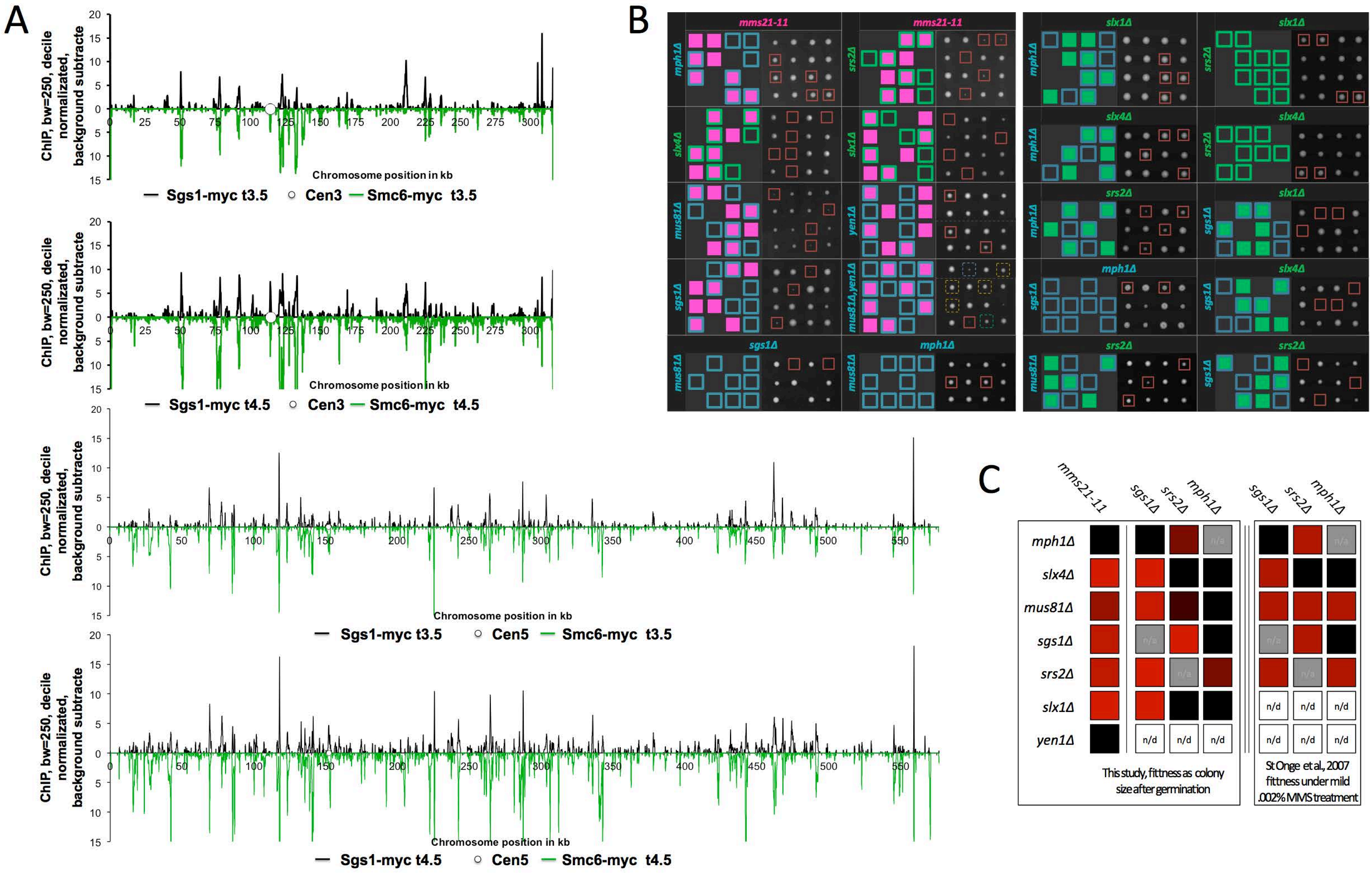
A
ChIP, bw=250, decile normalizated, background subtracte
Chromosome position in kb
Sgs1-myc t3.5
Cen3
Smc6-myc t3.5
Sgs1-myc t4.5
Cen3
Smc6-myc t4.5
Sgs1-myc t3.5
Cen5
Smc6-myc t3.5
Sgs1-myc t4.5
Cen5
Smc6-myc t4.5
B
mms21-11
slx1Δ
slx4Δ
srs2Δ
mph1Δ
sgs1Δ
mus81Δ
yen1Δ
mus81Δ,yen1Δ
C
mms21-11
sgs1Δ
srs2Δ
mph1Δ
mph1Δ
slx4Δ
mus81Δ
sgs1Δ
srs2Δ
slx1Δ
yen1Δ
n/a
n/d
This study, fitness as colony size after germination
St Onge et al., 2007 fitness under mild .002% MMS treatment

Figure S8

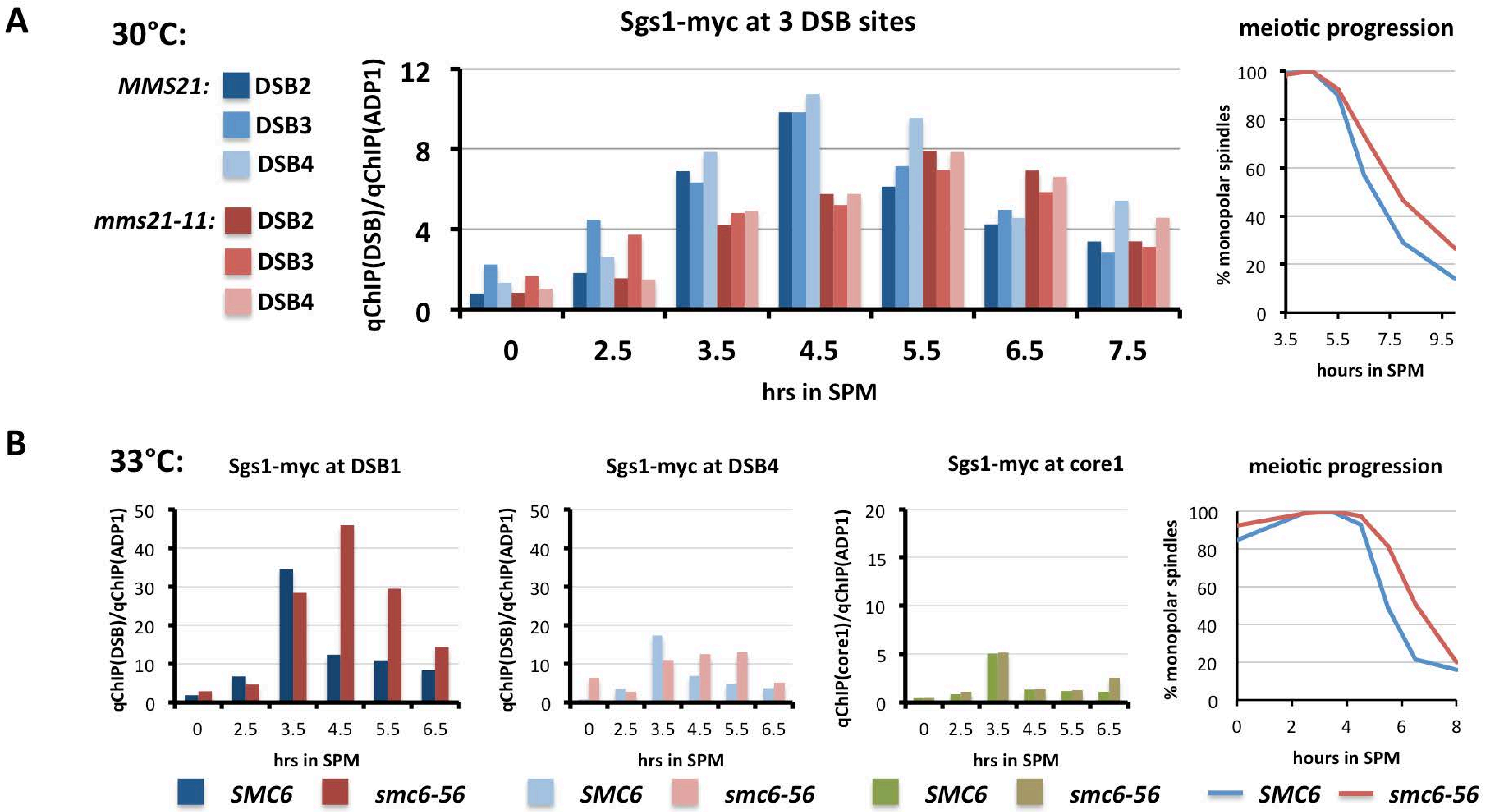
A
30°C:
MMS21: DSB2 DSB3 DSB4
mms21-11: DSB2 DSB3 DSB4
Sgs1-myc at 3 DSB sites
qChIP(DSB)/qChIP(ADP1)
0 4 8 12
0 2.5 3.5 4.5 5.5 6.5 7.5
hrs in SPM
meiotic progression
% monopolar spindles
0 20 40 60 80 100
3.5 5.5 7.5 9.5
hours in SPM
B
33°C:
Sgs1-myc at DSB1
qChIP(DSB)/qChIP(ADP1)
0 10 20 30 40 50
0 2.5 3.5 4.5 5.5 6.5
hrs in SPM
Sgs1-myc at DSB4
qChIP(DSB)/qChIP(ADP1)
0 10 20 30 40 50
0 2.5 3.5 4.5 5.5 6.5
hrs in SPM
Sgs1-myc at core1
qChIP(core1)/qChIP(ADP1)
0 5 10 15 20
0 2.5 3.5 4.5 5.5 6.5
hrs in SPM
meiotic progression
% monopolar spindles
0 20 40 60 80 100
0 2 4 6 8
hours in SPM
SMC6 smc6-56
SMC6 smc6-56
SMC6 smc6-56
SMC6 smc6-56

Figure S9

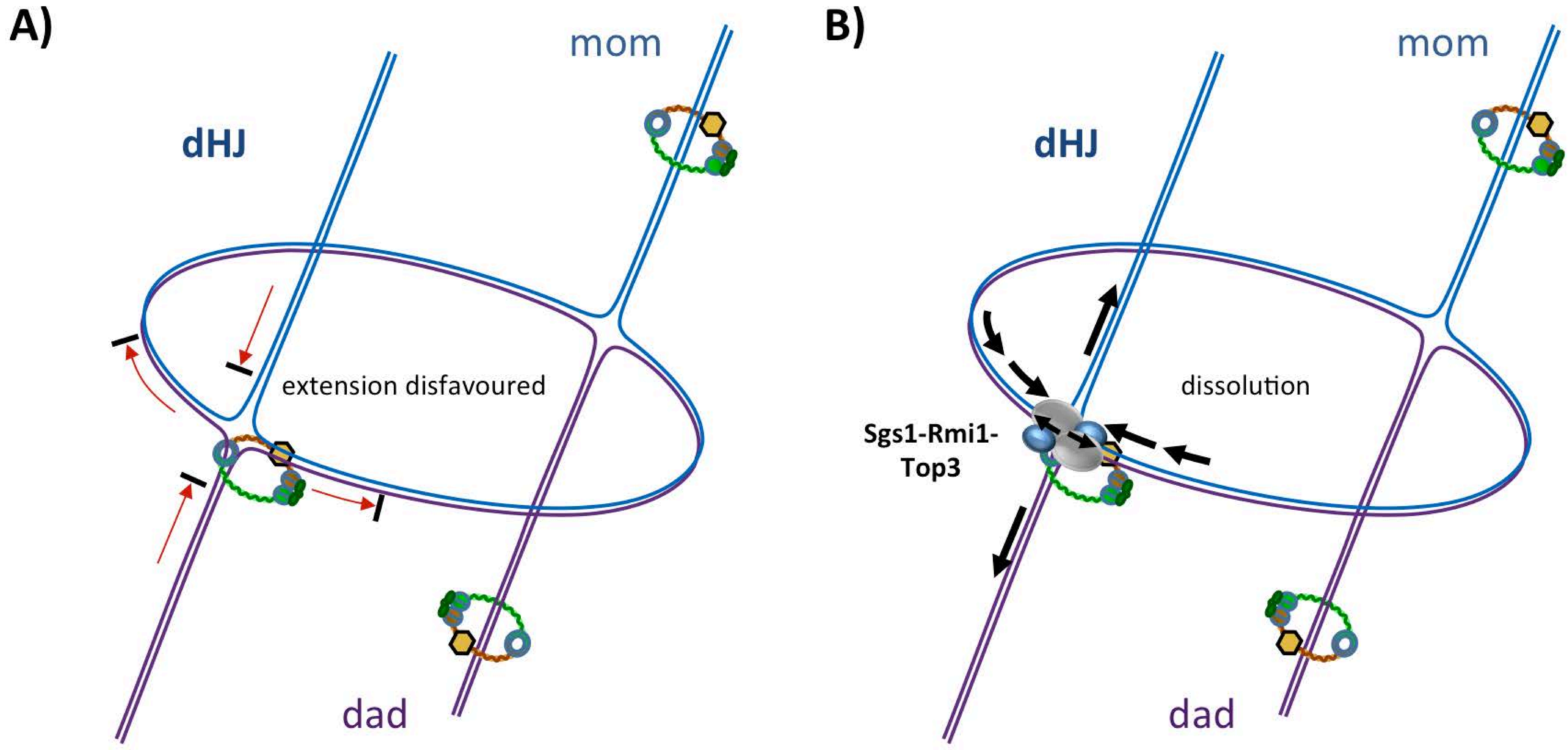

A)
dHJ
mom
extension disfavoured
dad
B)
dHJ
mom
dissolution
Sgs1-Rmi1-
Top3
dad

Table S1. Yeast Strains Used in This Study, Related to the Experimental Procedures

Strain	Relevant genotype
FKY407	Mat a/alpha, *zip1::LYS2*<br>*zip1::LYS2*
FKY483	Mat a/alpha, *zip1::LYS2, smc3-myc6::LEU2*<br>*zip1::LYS2, smc3-myc6::LEU2*
FKY1585	Mat alpha, *sgs1Δ::KanMX4*
FKY1610	Mat a, *sgs1Δ::KanMX4, leu2::hisG*
FKY1701	Mat a/alpha, *SGS1-MYC18::TRP1, trp1::hisG*<br>*SGS1-MYC18::TRP1, trp1::hisG*
FKY2053	Mat a/alpha, *mnd1Δ::KanMX*<br>*mnd1Δ::KanMX*
FKY2754	Mat a/alpha, *SMC6-MYC13::KanMX*<br>*SMC6-MYC13::KanMX*
FKY3108	Mat a/alpha, *his4Δ(Sal1-Cla1)::URA3-Δ(Sma1-Eco47III)-arg4-EcPal(1691),*<br>*arg4Δ(eco47III-hpa1)*<br>*leu2-RV::URA3-(Sma1-Eco47III)-ARG4, arg4Δ(eco47III-hpa1)*
FKY3321	Mat a/alpha, *pGAL-NDT80::TRP1, ura3::pGPD1-GAL4(848).ER::URA3*<br>*pGAL-NDT80::TRP1, ura3::pGPD1-GAL4(848).ER::URA3*
FKY4027	Mat a/alpha, *zip3Δ::KanMX4*<br>*zip3Δ::KanMX4*
FKY4066	Mat a/alpha, *ho::LYS2, lys2*<br>*ho::LYS2, lys2*
FKY4067	Mat a/alpha, *smc6-56*<br>*smc6-56*
FKY4115	Mat alpha, *mms21-11::LEU2, leu2::hisG*
FKY4116	Mat a, *mms21-11::LEU2, leu2::hisG*

FKY4117	Mat a/alpha, *mms21-11::LEU2, leu2::hisG*<br>*mms21-11::LEU2, leu2::hisG*
FKY4152	Mat a/alpha, *REC8-HA3::URA3, mms21-11::LEU2, leu2::hisG*<br>*REC8-HA3::URA3, mms21-11::LEU2, leu2::hisG*
FKY4153	Mat a/alpha, *REC8-HA3::URA3, smc6-56*<br>*REC8-HA3::URA3, smc6-56*
FKY4154	Mat a/alpha, *REC8-HA3::URA3, SMC6-MYC13::KanMX, ura3*<br>*REC8-HA3::URA3, SMC6-MYC13::KanMX, ura3*
FKY4171	Mat a/alpha, *zip3Δ::KanMX4, mms21-11::LEU2, leu2*<br>*zip3Δ::KanMX4, mms21-11::LEU2, leu2*
FKY4179	Mat a/alpha, *zip1::LYS2, smc3-myc6::LEU2, mms21-11::LEU2*<br>*zip1::LYS2, smc3-myc6::LEU2, mms21-11::LEU2*
FKY4201	Mat a/alpha, *SMC6-MYC13::KanMX, rec8Δ::KanMX4*<br>*SMC6-MYC13::KanMX, rec8Δ::KanMX4*
FKY4232	Mat a/alpha, *SMC5-HA3::KanMX, SMC6-MYC13::KanMX*<br>*SMC5-HA3::KanMX, SMC6-MYC13::KanMX*
FKY4236	Mat a/alpha, *SMC6-HA3::KanMX, SPO22-MYC9::TRP1, trp1::hisG*<br>*SMC6-HA3::KanMX, SPO22-MYC9::TRP1, trp1::hisG*
FKY4237	Mat a/alpha, *his4Δ(Sal1-Cla1)::URA3-Δ(Sma1-Eco47III)-arg4-EcPal(1691),*<br>*arg4Δ(eco47III-hpa1), smc6-56*<br>*leu2-RV::URA3-(Sma1-Eco47III)-ARG4, arg4Δ(eco47III-hpa1), smc6-56*
FKY4238	Mat a/alpha, *his4Δ(Sal1-Cla1)::URA3-Δ(Sma1-Eco47III)-arg4-EcPal(1691),*<br>*arg4Δ(eco47III-hpa1), mms21-11::LEU2*<br>*leu2-RV::URA3-(Sma1-Eco47III)-ARG4, arg4Δ(eco47III-hpa1),*<br>*mms21-11::LEU2*
FKY4357	Mat a/alpha, *mms21-11::LEU2, zip1Δ::KanMX4, leu2::hisG*<br>*mms21-11::LEU2, zip1Δ::KanMX4, leu2::hisG*
FKY4363	Mat a/alpha, *smc6-56, spo11::URA3, spo13::hisG, ura3*

	smc6-56, spo11::URA3, spo13::hisG, ura3
FKY4377	Mat a, *mus81Δ::KanMX*
FKY4405	Mat alpha, *mus81Δ::KanMX, leu2*
FKY4413	Mat a/alpha, *KanMX::pCLB2-3HA-MMS4*<br>*KanMX::pCLB2-3HA-MMS4*
FKY4414	Mat a/alpha, *mms21-11::LEU2, KanMX::pCLB2-3HA-MMS4, leu2::hisG*<br>*mms21-11::LEU2, KanMX::pCLB2-3HA-MMS4, leu2::hisG*
FKY4437	Mat a, *mms21-11::LEU2, yen1Δ::KanMX4, leu2::hisG*
FKY4454	Mat a/alpha, *pGAL-NDT80::TRP1, ura3::pGPD1-GAL4(848).ER::URA3, smc6-56*<br>*pGAL-NDT80::TRP1, ura3::pGPD1-GAL4(848).ER::URA3, smc6-56*
FKY4558	Mat alpha, *yen1Δ::KanMX4, leu2::hisG*
FKY4573	Mat a/alpha, *his4Δ(Sal1-Cla1)::URA3-Δ(Sma1-Eco47III)-arg4-EcPal(1691),*<br>*arg4Δ(eco47III-hpa1), mms21-11::LEU2, KanMX::pCLB2-3HA-MMS4*<br>*leu2-RV::URA3-(Sma1-Eco47III)-ARG4, arg4Δ(eco47III-hpa1),*<br>*mms21-11::LEU2, KanMX::pCLB2-3HA-MMS4*
FKY4776	Mat a/alpha, *his4Δ(Sal1-Cla1)::URA3-Δ(Sma1-Eco47III)-arg4-EcPal(1691),*<br>*arg4Δ(eco47III-hpa1), KanMX::pCLB2-3HA-MMS4*<br>*leu2-RV::URA3-(Sma1-Eco47III)-ARG4, arg4Δ(eco47III-hpa1),*<br>*KanMX::pCLB2-3HA-MMS4*
FKY4793	Mat a, *slx1Δ::NatMX4, leu2::hisG*
FKY4796	Mat a, *slx4Δ::NatMX4, leu2::hisG*
FKY4803	Mat a/alpha, *SGS1-MYC18::TRP1, mms21-11::LEU2, trp1::hisG, leu2::hisG*<br>*SGS1-MYC18::TRP1, mms21-11::LEU2, trp1::hisG, leu2::hisG*
FKY4805	Mat a/alpha, *smc6-56, zip3::KanMX*<br>*smc6-56, zip3::KanMX*
FKY5028	Mat a/alpha, *TOP3-HA3::KanMX6, smc6-56*<br>*TOP3-HA3::KanMX6, smc6-56*
FKY5054	Mat a/alpha, *TOP3-HA3::KanMX6*

	TOP3-HA3::KanMX6
FKY5202	Mat alpha, *mph1Δ::KanMX, leu2::hisG*
FKY5247	Mat a/alpha, *zip3Δ::KanMX, sgs1::KanMX::pCLB2-3HA-SGS1*<br>*zip3Δ::KanMX, sgs1::KanMX::pCLB2-3HA-SGS1*
FKY5249	Mat a/alpha, *his4Δ(Sal1-Cla1)::URA3-Δ(Sma1-Eco47III)-arg4-EcPal(1691),*<br>*arg4Δ(eco47III-hpa1), sgs1::KanMX::pCLB2-3HA-SGS1*<br>*leu2-RV::URA3-(Sma1-Eco47III)-ARG4, arg4Δ(eco47III-hpa1),*<br>*sgs1::KanMX::pCLB2-3HA-SGS1*
FKY5250	Mat a/alpha, *his4Δ(Sal1-Cla1)::URA3-Δ(Sma1-Eco47III)-arg4-EcPal(1691),*<br>*arg4Δ(eco47III-hpa1), sgs1::KanMX::pCLB2-3HA-SGS1, smc6-56*<br>*leu2-RV::URA3-(Sma1-Eco47III)-ARG4, arg4Δ(eco47III-hpa1),*<br>*sgs1::KanMX::pCLB2-3HA-SGS1, smc6-56*
FKY5351	Mat a/alpha, *SGS1-MYC18::TRP1, smc6-56, trp1::hisG*<br>*SGS1-MYC18::TRP1, smc6-56, trp1::hisG*
FKY5478	Mat a/alpha, *smc6-56, spo11::URA3, ura3*<br>*smc6-56, spo11::URA3, ura3*
FKY5483	Mat alpha, *srs2Δ::NatMX4, leu2::hisG*
FKY5607	Mat a/alpha, *zip3Δ::KanMX, KanMX::pCLB2-3HA-MMS4, slx1Δ::NatMX4,*<br>*yen1Δ::KanMX4*<br>*zip3Δ::KanMX, KanMX::pCLB2-3HA-MMS4, slx1Δ::NatMX4,*<br>*yen1Δ::KanMX4*
FKY5608	Mat a/alpha, *nse4::KanMX6::pCLB2-HA3-NSE4*<br>*nse4::KanMX6::pCLB2-HA3-NSE4*
FKY5609	Mat a/alpha, *zip3Δ::KanMX, nse4::KanMX6::pCLB2-HA3-NSE4*<br>*zip3Δ::KanMX, nse4::KanMX6::pCLB2-HA3-NSE4*
FKY5642	Mat a/alpha, *smc6-56, mnd1Δ::KanMX4*<br>*smc6-56, mnd1Δ::KanMX4*
FKY5662	Mat a/alpha, *zip3Δ::KanMX, KanMX::pCLB2-3HA-MMS4, slx1Δ::NatMX4,*

	yen1Δ::KanMX4, mlh3Δ::hphMX4<br>*zip3Δ::KanMX, KanMX::pCLB2-3HA-MMS4, slx1Δ::NatMX4,*<br>*yen1Δ::KanMX4, mlh3Δ::hphMX4*

All strains used in this study are isogenic derivatives of SK1.

Table S2. Antibodies used in this study.

animal	conjugated to	specificity	Source	dilution used
goat	CY3	anti-mouse	Jackson Immuno, 115-165-146	1:400 cytology
mouse		anti-myc (9E10)	Kim Nasmyth, Wolfgang Zachariae	1:35 cytology
mouse		anti-HA (16B12)	Ralf Hess, MMS-101R	1:1500 cytology
rabbit		anti-Zip1	Eurogentec,Wohlrab, FKab104	1:50 cytology
goat	Alexa488	anti-rabbit	Molecular Probes, A-11034	1:300 cytology
mouse		anti-Rad51	NeoMarkers, MS-988-P0	1:50 cytology
rabbit		anti-HA	Sigma, H6908	1:100 cytology
rabbit		anti-myc	Gramsch, CM-100	1:500 cytology
rabbit		anti-Hop1	Eurogentec, Ferscha, FKab206	1:50 cytology
rat		anti-tubulin	Serotec, MCA78	1:200 cytology
rabbit	FITC	anti-rat	Sigma, F1763	1:100 cytology

Supplemental Figure Legends :

Figure S1. Meiosis specific depletion of Smc6 leaves meiotic progression and chromosome synapsis unaffected (Related to Figure 1)

(A) A time series of FACS profiles for pre-meiotic DNA replication in the *smc6-56* mutant from indicated time points post induction of meiosis and under our regiment for gradual inactivation of Smc5/6 at late S-phase. Subsequent meiotic progression is shown for this time course experiment on meiotic spindles (n=200 for each time point), as exit from meiotic prophase 1.

(B) The semi-permissive temperature of 30°C for the *smc6-56* allele permits meiotic DNA segregation while the restrictive temperature of 33°C does not. Meiotic nuclei of ethanol fixed and DAPI stained cells were counted at the indicated time points and temperatures (n=200). The sum of bi- and tetra-nucleated cells (2n+4n) is plotted.

(C) Upper panels: Three spread nuclei representing examples of pachytene cells showing no aberrant features from *wild type*, *smc6-56* and *mms21-11* mutants as indicated. White: DAPI stained chromosomes, Green: Zip1 protein demonstrating full synapsis, Red: Few, normal residual Rad51 foci.

Lower panels: Quantification of synapsis in *wild type*, *smc6-56* and *mms21-11* diploids from 4, 5 and 6 hour time points of sporulation (different shades of blue) at the temperatures indicated. All mutants form normal SC, although *mms21-11* showed a reduced yield of extended and full SCs at lower temperature.

Figure S2. Binding pattern of Smc6 on meiotic chromatin by cytology (Related to Figure 2)

(A) Smc5-HA3 and Smc6-myc13 foci colocalize: Red: Smc6-myc13, Green: Smc5-HA3, white bar: 10µm. White rectangle indicates position of magnified sub region. Co-localization for 1689 foci of 8 prophase 1 nuclei was determined.

(B) Smc6-myc13 localizes early to meiotic chromatin, concomitant with the appearance of Rec8-HA3 foci. 5 nuclei (from 2h in SPM) represent consecutive stages from left to right, based on the abundance of Rec8-HA foci. Panels from top: Rec8-HA3, Smc6-myc13, chromatin stained with DAPI. White bar: 10µm. Colored Panel: Overlay of Rec8 and Smc6 signals reveal very little overlap in early nuclei. Red: Smc6-myc13, Green: Rec8-HA3, White: DAPI. White rectangle indicates position of magnified sub region. Upon close inspection, none of the early Rec8 and Smc6 foci in the region show significant overlap.

(C) Partial co-localization between Zip4-myc9 and Smc6-HA3. Nuclei from 4 hours in SPM show limited on top but frequent side by side localization, similar to the situation between Rad51 and Smc6. Red: Smc6-HA3, green: Zip4-myc9, white bar: 10µm. White rectangle indicates position of magnified sub region.

Figure S3. Chromatin binding of Smc6 throughout meiosis by cytology (Related to Figure 2)

35 spread nuclei of various meiotic stages sorted in a consecutive order from left to right and from top to bottom by combining information from Zip1 and DAPI morphologies. Each nucleus is represented by three micrographs, a green and red merge (between Zip1 and Smc6, respectively), a merge between blue and red (DAPI and Smc6) and a white micrograph (DAPI staining of DNA). The upper three rows depict stages from the earliest appearance of Smc6 representing presumably (early) replicating nuclei, to leptotene and zygotene. The three lanes in the middle represent zygotene, pachytene and post pachytene stages (presumably corresponding to diplotene). Three white arrowheads point at dense clusters of Smc6 foci accumulating at the unsynapsed regions of chromosome XII, representing the rDNA. The bottom three lanes correspond to late stages: Prometaphase/metaphase, as well as several anaphase I and anaphase II examples. Three white arrowheads point at dense clusters of Smc6 foci accumulating at the residual connecting chromatin between the almost pinched off nuclei. White bar: 10µm.

Figure S4. Smc6 is recruited to meiotic DSB hotspots where it enriches to upon DSB formation (Related to Figure 2D,E)

ChIP-seq profiles for complete chromosomes. (A, B) The upper two panels show chromosome III, the lower two chromosome V. Of the two panels depicting the same chromosome the upper one corresponds to time point 3.5 hours in SPM, the lower one to 4.5 hours in SPM. The color coding is: Black: Smc6-myc13, blue: Smc6-myc13, *spo11Δ*, red: DSBs shown on the negative axis for better comparison. A profile of chromosome axis protein Hop1 (taken from [1]) was added in grey to mark axis sites. Appropriately colored arrows point to sites where the primers for cold region, hotspot and core used for qPCR are located. Here ChIP-seq profiles were smoothed (bw=250bp), decile normalized and background subtracted (except for Hop1).

(C) qPCR of a biological repeat at three positions on chromosome III from the same experiment, a DSB site (ca. at 211k, YCR047C), a core site (ca. at 219k) and a cold spot (ca. at 136, ADP1). To correct for possible differences in the efficiencies of the IPs across the different strains, the enrichment of core and DSB qPCR signals relative to the ADP1 signal is plotted for the indicated genotypes and time points, Core/ADP1 shown in blue, DSB/ADP1 in red.

(D) Overlap of Smc6 (4.5h) peaks with the 1000 strongest DSB hotspots [2]. A peak is considered overlapping if it lies within the borders of the hotspot. Left column: % of DSB hotpots that are hit by Smc6 peaks. Middle column: The number of Smc6 peaks required to hit the corresponding percentage of DSB hotspots. Right column: The significance (binomial random model), which was in all cases below the smallest value displayed by R (10^{-15}). Because the strongest hotspots do not always correspond to the strongest Smc6 peaks, naturally, there is only 34% overlap between the 1000 strongest Smc6 (t4.5) and DSB sites. However, nearly all of the 1000 strongest DSB hotspots contain an Smc6 peak if smaller Smc6 peaks are also considered (e.g. 80% when considering 6000 Smc6 peaks). The significance of this co-localization is still very high (p(random model)<10^{-15}).

Figure S5. Smc5/6 mutants promote the formation of inappropriate, stable recombination intermediates (Related to Figure 4)

(A) *smc6-56* does not promote axis pairing in the *mnd1Δ* mutant which is defective for the DSB strand invasion step in meiotic homologous recombination. Upper panel: Quantification of bivalent formation on spread nuclei of 5hrs SPM stained against Hop1 (n=50 for each mutant). Number of incidences is plotted for each category of “0” to “16” bivalents. Dark blue: *mnd1*Δ, blue: *mnd1*Δ *smc6-56*, red: *zip3*Δ, green: *zip3*Δ *smc6-56* (all at 33°C). Lower panel: Representative nuclei of the indicated mutants at 5hrs SPM after chromosome spreading and staining against Hop1 (green).

(B) Inactivation of the rogue JM resolvases cannot promote stabilization of axial associations in the *zip3Δ* mutant while inactivation of Smc5/6-Mms21 does, indicative of inappropriate JM formation. As in (A): Upper panel: Quantification of bivalent formation on spread nuclei of 5hrs SPM stained against Hop1 (n=100 for each mutant). Blue: *zip3*Δ, red: *zip3Δ mms4-mn slx1Δ yen1Δ*, green: *zip3*Δ *nse4-mn* (at 28°C). Lower panel: Representative nuclei of the indicated mutants at 5hrs SPM. Hop1 in green.

(C) Removal of all characterized meiotic resolvase activity cannot promote chromosome pairing in *zip3Δ* such as observed in *zip3Δ sgs1-mn* and *zip3Δ nse4-mn*. Left panel: Quantification of bivalent formation on spread nuclei of 5hrs SPM stained against Hop1, plotted as percentage of incidents (n=150 for each mutant). Blue: *zip3*Δ, pink: *zip3Δ mms4-mn slx1Δ yen1Δ mlh3Δ*, light green: *zip3*Δ *nse4-mn,* dark green: *zip3*Δ *sgs1-mn* (at 30°C). Right panel: Representative nuclei of the indicated mutants at 5hrs SPM. Hop1 in green.

Figure S6. Inactivation of Smc5/6-Mms21 at pachytene exit prevents nuclear divisions (Related to Figure 5)

A shift to restrictive temperature during *ndt80* release abolishes nuclear divisions in *smc6-56,* indicating that early function of Smc5/6-Mms21 is not sufficient to relieve the block of division, but that late functions are required. The experiment is related to Figure 5 (H,I), except that in this experiment the temperature regime was inverted, starting with meiosis at permissive temperature until the release from the *ndt80-IN* arrest. Left panels show meiotic progression in response to estradiol addition, as monitored by spindle staining. The permissive (23.5°C) and restrictive (33°C) temperatures are indicated by turquoise and orange colors above the bar diagram. ED indicates the timing of addition of estradiol to induced Ndt80 expression and exit from pachytene. In contrast, a shift to permissive temperature during Ndt80 release restores nuclear divisions in *smc6-56* (Figure 5I). Upper panels: Wild type (*ndt80-IN*), lower panels: *smc6-56* (*ndt80-IN*). Light green bars: Monopolar spindles, dark green bars bipolar spindles. From left to right: 1st panel: Time course starting under permissive conditions in the n*dt80-IN* strain background in the absence of inducer (estradiol). 2nd panel: shift to restrictive temperature (33°C), without release (- estradiol). 3rd panel: release (+ estradiol) into restrictive temperature (causes short and synchronous burst of bipolar spindles). 4rd panel: release (+ estradiol) of cells that started meiosis under restrictive conditions into restrictive temperature (positive control for blocked divisions, causes short and synchronous burst of bipolar spindles).

The four diagrams on the right show nuclear divisions as a function of time post Ndt80 expression: % 1n (circle), 2n (triangle) and 4n (squares, n= number of nuclei) plotted against hours in SPM. Upper two panels show normal divisions in *SMC6*, independent of treatments. Lower panels: Nuclear divisions are blocked, irrespective of whether they spent their prophase (DSB repair) under restrictive or permissive conditions, although the chromatin appears more flexible during anaphases if Smc5/6-Mms21 was provided during DSB repair.

Figure S7: Analogies between Smc5/6-Mms21 and Sgs1 (Related to Figure 6)

(A) Chromosomal interaction sites and intensities are highly similar between Sgs1 and Smc6. DNA-interaction sites for Sgs1-myc18 (t = 4.5 hours in SPM) and Smc6-myc13 (t = 3.5 hours in SPM) on two full length profiles are shown for chromosome III (upper two diagrams) and chromosome V (lower two diagrams). (Full datasets are available at GEO, accession number GSE51977). ChIP-seq signals were smoothed at bandwidth 250bp, decile normalized and background subtracted (minus untagged). White circle: Centromere. Black: Sgs1. Smc6 signals were plotted in green on the negative scale to facilitate comparison. The apparent Sgs1/Smc6 symmetry in the example region is corroborated genome-wide by the high Pearson correlation (pcorr=0.8) over more than 6000 peaks per profile.

(B) Vegetative synthetic interactions of the *mms21-11* mutant with resolvase and anti-recombinogenic helicase mutants assayed by colony size after spore germination. The relevant genotypes are indicated by color code (to the left of the spore colonies), red squares over colonies indicates the presence of double or triple mutations. For the *mus81Δ yen1Δ mms21-11* panel, yellow dashed squares are *mms21-11 yen1Δ*, the blue dashed square *mms21-11 mus81Δ* and the green dashed square *mus81Δ yen1Δ*.

(C) Summary of observed synthetic interactions (colony size) and comparison with published ones (generation time in .002% MMS), [3]. Black indicates absence of synthetic growth defect, consecutive brighter shades of red indicate stronger synthetic effects (colony size). (n/a: not applicable, n/d: not determined)

Figure S8: Smc5/6-Mms21 is not essential for Sgs1 recruitment to meiotic DSB sites (Related to Figure 6C, D)

(A) Sgs1 does not require Mms21 SUMOylation activity for recruitment to DSB hotspots. qChIP of Sgs1-myc18 at various time points in meiosis (30°C) at three different DSB sites (DSB2: 130,7kb [Chr I], DSB3: 190,5kb [Chr I], DSB4: 96,1kb [Chr IV]) shown for *MMS21* (blue bars) and *mms21-11* (red bars). Different shades represent the different hotspots. The Sgs1 signals in *mms21-11* follow those of wild type with a 1 hour delay. The right panel documents meiotic progression by spindle morphology of the same culture, revealing a 1 hour delay in the mutant culture. This delay is most likely due to synthety with the C-terminal myc-tag of Sgs1 because untagged *mms21-11* cells do not show a meiotic delay.

(B) Sgs1 does not require Smc6 for recruitment to DSB hotspots at 33°C. qChIP for Sgs1-myc18 at various time points during meiosis (33°C) at two different DSB sites (DSB1: 211kb [Chr III], DSB4: 96,1kb [Chr IV]) shown for *SMC6* (blue bars) and *smc6-56* (red bars) and at Core1: 219,1kb [Chr III] (*SMC6* (green bars) and *smc6-56* (brown bars)). The kinetics of Sgs1 signals in *smc6-56* appear to lag 1 hour behind. The right panel documents meiotic progression by spindle morphology of the same culture.

Figure S9: Topological model for early Smc5/6-Mms21 functions on dHJs (Related to Figure 7)

(A) Surveying for displaced ssDNA as a preloaded complex will inherently lock Smc5/6-Mms21 to the "outside" of recombinogenic lesions. In the case of a dHJ, such binding will label the parental side of

the dHJ, opposed to the hetero-duplex DNA region. Stable binding to the HJ will subsequently disfavor dHJ extension as base pairing may be inhibited and dHJ extension would require overcoming a protruding parental DNA strand.

(B) A single HJ is devoid of information regarding the inside or outside regions of a dHJ. At the least, inter-sister dHJs lack any hetero-duplex DNA which could help to distinguish the inside of a lesion from the outside. Thus, inappropriate association of Sgs1-Rmi1-Top3 may result in dHJ extension instead of dissolution. As preloaded Smc5/6-Mms21 binding to the ssDNA of a HJ will label the parental side of a dHJ, Smc5/6-Mms21 may provide a polarity to the HJ and guide the activity of Sgs1-Rmi1-Top3 towards dissolution of the dHJ.

Table S1: Yeast strains used in this study

Table S2: Antibodies used in this study

Supporting Information File S1: A single pdf file containing all supporting figures, supporting tables and corresponding legends is provided for convenience.
