# Supplementary material for: Smc5/6-Mms21 Prevents and Eliminates Inappropriate Recombination Intermediates in Meiosis
Source: PLoS Genet. 2013 Dec 26;9(12):e1004067. doi: 10.1371/journal.pgen.1004067 (PMC3873250; doi:10.1371/journal.pgen.1004067)
Supplement: Table S1 — Yeast strains used in this study. (DOCX) [file pgen.1004067.s011.docx]

| ***Table S1. Yeast Strains Used in This Study, Related to the Experimental Procedures*** | |
| --- | --- |
|  |  |
| Strain | Relevant genotype |
| *FKY407* | Mat a/alpha, *zip1::LYS2* |
|  | *zip1::LYS2* |
| *FKY483* | Mat a/alpha, *zip1::LYS2, smc3-myc6::LEU2* |
|  | *zip1::LYS2, smc3-myc6::LEU2* |
| *FKY1585* | Mat alpha, *sgs1∆::KanMX4* |
| *FKY1610* | Mat a, *sgs1∆::KanMX4, leu2::hisG* |
| *FKY1701* | Mat a/alpha, *SGS1-MYC18::TRP1, trp1::hisG* |
|  | *SGS1-MYC18::TRP1, trp1::hisG* |
| *FKY2053* | Mat a/alpha, *mnd1∆::KanMX*  *mnd1∆::KanMX* |
| *FKY2754* | Mat a/alpha, *SMC6-MYC13::KanMX* |
|  | *SMC6-MYC13::KanMX* |
| *FKY3108* | Mat a/alpha, *his4∆(Sal1-Cla1)::URA3-∆(Sma1-Eco47III)-arg4-EcPal(1691),* |
|  | *arg4∆(eco47III-hpa1)* |
|  | *leu2-RV::URA3-(Sma1-Eco47III)-ARG4, arg4∆(eco47III-hpa1)* |
| *FKY3321* | Mat a/alpha, *pGAL-NDT80::TRP1, ura3::pGPD1-GAL4(848).ER::URA3* |
|  | *pGAL-NDT80::TRP1, ura3::pGPD1-GAL4(848).ER::URA3* |
| *FKY4027* | Mat a/alpha, *zip3∆::KanMX4* |
|  | *zip3∆::KanMX4* |
| *FKY4066* | Mat a/alpha, *ho::LYS2, lys2* |
|  | *ho::LYS2, lys2* |
| *FKY4067* | Mat a/alpha, *smc6-56* |
|  | *smc6-56* |
| *FKY4115* | Mat alpha, *mms21-11::LEU2, leu2::hisG* |
| *FKY4116* | Mat a, *mms21-11::LEU2, leu2::hisG* |
| *FKY4117* | Mat a/alpha, *mms21-11::LEU2, leu2::hisG* |
|  | *mms21-11::LEU2, leu2::hisG* |
| *FKY4152* | Mat a/alpha, *REC8-HA3::URA3, mms21-11::LEU2, leu2::hisG* |
|  | *REC8-HA3::URA3, mms21-11::LEU2, leu2::hisG* |
| *FKY4153* | Mat a/alpha, *REC8-HA3::URA3, smc6-56* |
|  | *REC8-HA3::URA3, smc6-56* |
| *FKY4154* | Mat a/alpha, *REC8-HA3::URA3, SMC6-MYC13::KanMX, ura3* |
|  | *REC8-HA3::URA3, SMC6-MYC13::KanMX, ura3* |
| *FKY4171* | Mat a/alpha, *zip3∆::KanMX4, mms21-11::LEU2, leu2* |
|  | *zip3∆::KanMX4, mms21-11::LEU2, leu2* |
| *FKY4179* | Mat a/alpha, *zip1::LYS2, smc3-myc6::LEU2, mms21-11::LEU2* |
|  | *zip1::LYS2, smc3-myc6::LEU2, mms21-11::LEU2* |
| *FKY4201* | Mat a/alpha, *SMC6-MYC13::KanMX, rec8∆::KanMX4* |
|  | *SMC6-MYC13::KanMX, rec8∆::KanMX4* |
| *FKY4232* | Mat a/alpha, *SMC5-HA3::KanMX, SMC6-MYC13::KanMX* |
|  | *SMC5-HA3::KanMX, SMC6-MYC13::KanMX* |
| *FKY4236* | Mat a/alpha, *SMC6-HA3::KanMX, SPO22-MYC9::TRP1, trp1::hisG* |
|  | *SMC6-HA3::KanMX, SPO22-MYC9::TRP1, trp1::hisG* |
| *FKY4237* | Mat a/alpha, *his4∆(Sal1-Cla1)::URA3-∆(Sma1-Eco47III)-arg4-EcPal(1691),* |
|  | *arg4∆(eco47III-hpa1), smc6-56* |
|  | *leu2-RV::URA3-(Sma1-Eco47III)-ARG4, arg4∆(eco47III-hpa1), smc6-56* |
| *FKY4238* | Mat a/alpha, *his4∆(Sal1-Cla1)::URA3-∆(Sma1-Eco47III)-arg4-EcPal(1691),* |
|  | *arg4∆(eco47III-hpa1), mms21-11::LEU2* |
|  | *leu2-RV::URA3-(Sma1-Eco47III)-ARG4, arg4∆(eco47III-hpa1),* |
|  | *mms21-11::LEU2* |
| *FKY4357* | Mat a/alpha, *mms21-11::LEU2, zip1∆::KanMX4, leu2::hisG* |
|  | *mms21-11::LEU2, zip1∆::KanMX4, leu2::hisG* |
| *FKY4363* | Mat a/alpha, *smc6-56, spo11::URA3, spo13::hisG, ura3* |
|  | *smc6-56, spo11::URA3, spo13::hisG, ura3* |
| *FKY4377* | Mat a, *mus81∆::KanMX* |
| *FKY4405* | Mat alpha, *mus81∆::KanMX, leu2* |
| *FKY4413* | Mat a/alpha, *KanMX::pCLB2-3HA-MMS4* |
|  | *KanMX::pCLB2-3HA-MMS4* |
| *FKY4414* | Mat a/alpha, *mms21-11::LEU2, KanMX::pCLB2-3HA-MMS4, leu2::hisG* |
|  | *mms21-11::LEU2, KanMX::pCLB2-3HA-MMS4, leu2::hisG* |
| *FKY4437* | Mat a, *mms21-11::LEU2, yen1∆::KanMX4, leu2::hisG* |
| *FKY4454* | Mat a/alpha, *pGAL-NDT80::TRP1, ura3::pGPD1-GAL4(848).ER::URA3, smc6-56* |
|  | *pGAL-NDT80::TRP1, ura3::pGPD1-GAL4(848).ER::URA3, smc6-56* |
| *FKY4558* | Mat alpha, *yen1∆::KanMX4, leu2::hisG* |
| *FKY4573* | Mat a/alpha, *his4∆(Sal1-Cla1)::URA3-∆(Sma1-Eco47III)-arg4-EcPal(1691),* |
|  | *arg4∆(eco47III-hpa1), mms21-11::LEU2, KanMX::pCLB2-3HA-MMS4* |
|  | *leu2-RV::URA3-(Sma1-Eco47III)-ARG4, arg4∆(eco47III-hpa1),* |
|  | *mms21-11::LEU2, KanMX::pCLB2-3HA-MMS4* |
| *FKY4776* | Mat a/alpha, *his4∆(Sal1-Cla1)::URA3-∆(Sma1-Eco47III)-arg4-EcPal(1691),* |
|  | *arg4∆(eco47III-hpa1), KanMX::pCLB2-3HA-MMS4* |
|  | *leu2-RV::URA3-(Sma1-Eco47III)-ARG4, arg4∆(eco47III-hpa1),* |
|  | *KanMX::pCLB2-3HA-MMS4* |
| *FKY4793* | Mat a, *slx1∆::NatMX4, leu2::hisG* |
| *FKY4796* | Mat a, *slx4∆::NatMX4, leu2::hisG* |
| *FKY4803* | Mat a/alpha, *SGS1-MYC18::TRP1, mms21-11::LEU2, trp1::hisG, leu2::hisG*  *SGS1-MYC18::TRP1, mms21-11::LEU2, trp1::hisG, leu2::hisG* |
| *FKY4805* | Mat a/alpha, *smc6-56, zip3::KanMX*  *smc6-56, zip3::KanMX* |
| *FKY5028* | Mat a/alpha, *TOP3-HA3::KanMX6, smc6-56* |
|  | *TOP3-HA3::KanMX6, smc6-56* |
| *FKY5054* | Mat a/alpha, *TOP3-HA3::KanMX6* |
|  | *TOP3-HA3::KanMX6* |
| *FKY5202* | Mat alpha, *mph1∆::KanMX, leu2::hisG* |
| *FKY5247* | Mat a/alpha, *zip3Δ::KanMX, sgs1::KanMX::pCLB2-3HA-SGS1*  *zip3Δ::KanMX, sgs1::KanMX::pCLB2-3HA-SGS1* |
| *FKY5249* | Mat a/alpha, *his4∆(Sal1-Cla1)::URA3-∆(Sma1-Eco47III)-arg4-EcPal(1691),* |
|  | *arg4∆(eco47III-hpa1), sgs1::KanMX::pCLB2-3HA-SGS1* |
|  | *leu2-RV::URA3-(Sma1-Eco47III)-ARG4, arg4∆(eco47III-hpa1),* |
|  | *sgs1::KanMX::pCLB2-3HA-SGS1* |
| *FKY5250* | Mat a/alpha, *his4∆(Sal1-Cla1)::URA3-∆(Sma1-Eco47III)-arg4-EcPal(1691),* |
|  | *arg4∆(eco47III-hpa1), sgs1::KanMX::pCLB2-3HA-SGS1, smc6-56* |
|  | *leu2-RV::URA3-(Sma1-Eco47III)-ARG4, arg4∆(eco47III-hpa1),* |
|  | *sgs1::KanMX::pCLB2-3HA-SGS1, smc6-56* |
| *FKY5351* | Mat a/alpha, *SGS1-MYC18::TRP1, smc6-56, trp1::hisG*  *SGS1-MYC18::TRP1, smc6-56, trp1::hisG* |
| *FKY5478* | Mat a/alpha, *smc6-56, spo11::URA3, ura3* |
|  | *smc6-56, spo11::URA3, ura3* |
| *FKY5483* | Mat alpha, *srs2∆::NatMX4, leu2::hisG* |
| *FKY5607* | Mat a/alpha, *zip3∆::KanMX, KanMX::pCLB2-3HA-MMS4, slx1∆::NatMX4,*  *yen1∆::KanMX4*  *zip3∆::KanMX, KanMX::pCLB2-3HA-MMS4, slx1∆::NatMX4,*  *yen1∆::KanMX4* |
| *FKY5608* | Mat a/alpha, *nse4::KanMX6::pCLB2-HA3-NSE4*  *nse4::KanMX6::pCLB2-HA3-NSE4* |
| *FKY5609* | Mat a/alpha, *zip3∆::KanMX, nse4::KanMX6::pCLB2-HA3-NSE4*  *zip3∆::KanMX, nse4::KanMX6::pCLB2-HA3-NSE4* |
| *FKY5642* | Mat a/alpha, *smc6-56, mnd1∆::KanMX4*  *smc6-56, mnd1∆::KanMX4* |
| *FKY5662* | Mat a/alpha, *zip3∆::KanMX, KanMX::pCLB2-3HA-MMS4, slx1∆::NatMX4,*  *yen1∆::KanMX4, mlh3∆::hphMX4*  *zip3∆::KanMX, KanMX::pCLB2-3HA-MMS4, slx1∆::NatMX4,*  *yen1∆::KanMX4, mlh3∆::hphMX4* |
|  |  |
| *All strains used in this study are isogenic derivatives of SK1.* | |
