# Supplementary material for: Smc5/6-Mms21 Prevents and Eliminates Inappropriate Recombination Intermediates in Meiosis
Source: PLoS Genet. 2013 Dec 26;9(12):e1004067. doi: 10.1371/journal.pgen.1004067 (PMC3873250; doi:10.1371/journal.pgen.1004067)
Supplement: Table S2 — Antibodies used in this study. (DOCX) [file pgen.1004067.s012.docx]

Table S2. Antibodies used in this study.

| animal | conjugated to | specificity | Source | dilution used |
| --- | --- | --- | --- | --- |
| goat | CY3 | anti-mouse | Jackson Immuno, 115-165-146 | 1:400 cytology |
| mouse |  | anti-myc (9E10) | Kim Nasmyth, Wolfgang Zachariae | 1:35 cytology |
| mouse |  | anti-HA (16B12) | Ralf Hess, MMS-101R | 1:1500 cytology |
| rabbit |  | anti-Zip1 | Eurogentec,Wohlrab, FKab104 | 1:50 cytology |
| goat | Alexa488 | anti-rabbit | Molecular Probes, A-11034 | 1:300 cytology |
| mouse |  | anti-Rad51 | NeoMarkers, MS-988-P0 | 1:50 cytology |
| rabbit |  | anti-HA | Sigma, H6908 | 1:100 cytology |
| rabbit |  | anti-myc | Gramsch, CM-100 | 1:500 cytology |
| rabbit |  | anti-Hop1 | Eurogentec, Ferscha, FKab206 | 1:50 cytology |
| rat |  | anti-tubulin | Serotec, MCA78 | 1:200 cytology |
| rabbit | FITC | anti-rat | Sigma, F1763 | 1:100 cytology |
